# Supplementary figures and images for: Conformational changes in the Ebola virus membrane fusion machine induced by pH, Ca2+, and receptor binding
Source: PLoS Biol. 2020 Feb 10;18(2):e3000626. doi: 10.1371/journal.pbio.3000626 (PMC7034923; doi:10.1371/journal.pbio.3000626)

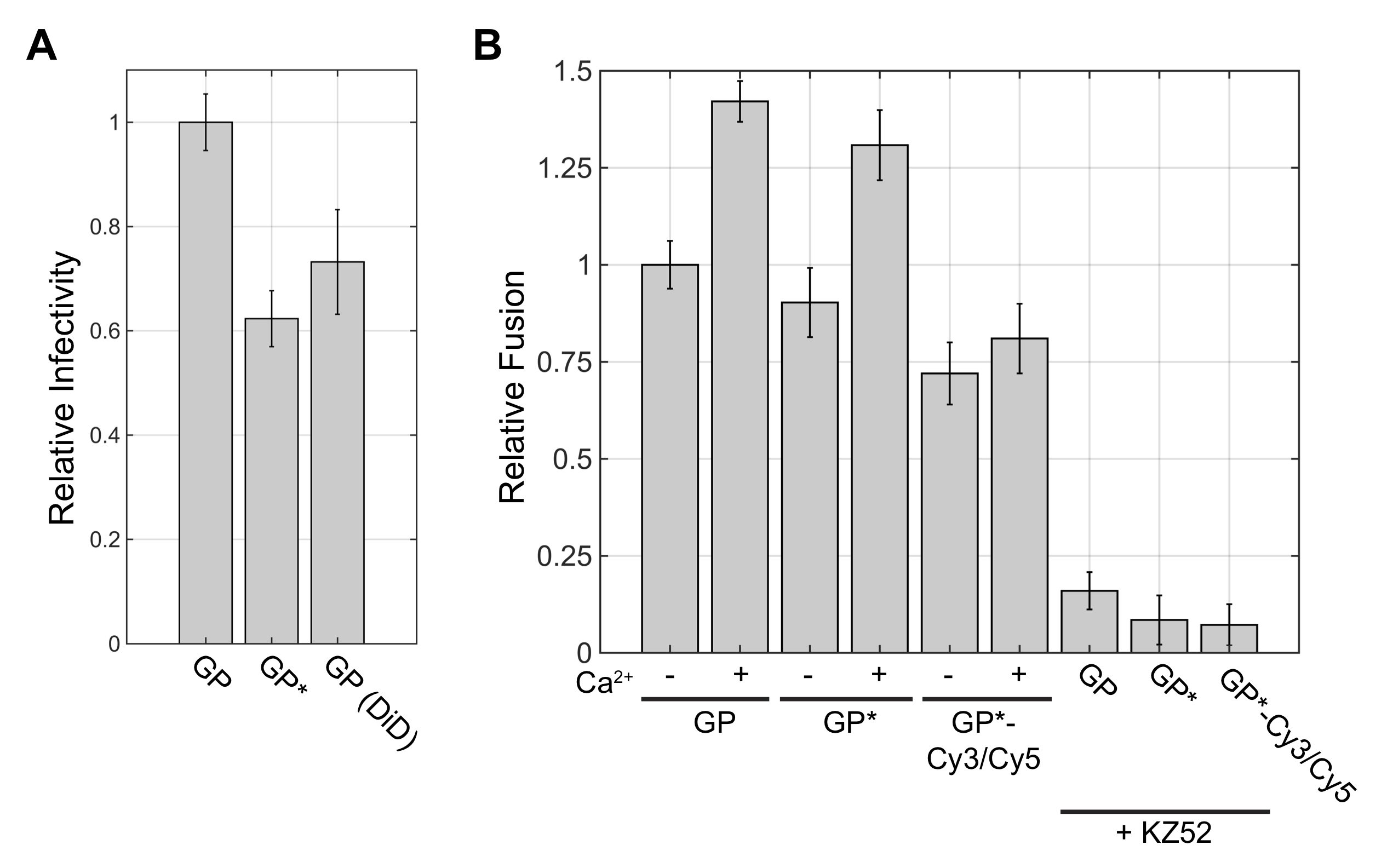

Supplement: S1 Fig — (A) Infectivity of GFP-expressing VSV particles pseudotyped with GP or GP* or labeled with the lipophilic fluorophore DiD. DiD was used in the infectivity measurements to evaluate the effect of a lipophilic fluorophore because it is spectrally distinct from GFP and chemically similar to the DiO fluorophore used in the lipid mixing assay. DiO excitation overlaps with that of GFP and thus could not be used in this assay. Infectivity is normalized to wild-type GP with an unlabeled virion. (B) Viral fusion was measured using a Blam-based virus entry assay (Materials and methods). HIV pseudovirions containing either GP, GP*, or GP*-Cy3/Cy5, and in the absence or presence of antibody KZ52. For the purpose of evaluating the functionality of GP* and GP*-Cy3-Cy5, pseudovirions were not formed with excess wild-type GP as done for smFRET imaging experiments. Pseudovirions were preincubated with 0.5 mM CaCl2 where indicated. Some enhancement of fusion was observed following the preincubation with CaCl2 indicating that the endosomal Ca2+ concentration in the target cells used here may be not be optimal for GP-mediated fusion. All GP variants were efficiently inhibited by KZ52, confirming that the TCO* residues do not perturb the global conformation of GP. Data are presented as the average of 3 independent measurements, with error bars reflecting the standard deviation. EBOV, Ebola virus; GFP, green fluorescent protein; GP, EBOV envelope glycoprotein; HIV, human immunodeficiency virus; smFRET, single-molecule Förster resonance energy transfer; VSV, vesicular stomatitis virus. (TIF) [file pbio.3000626.s001.tif]

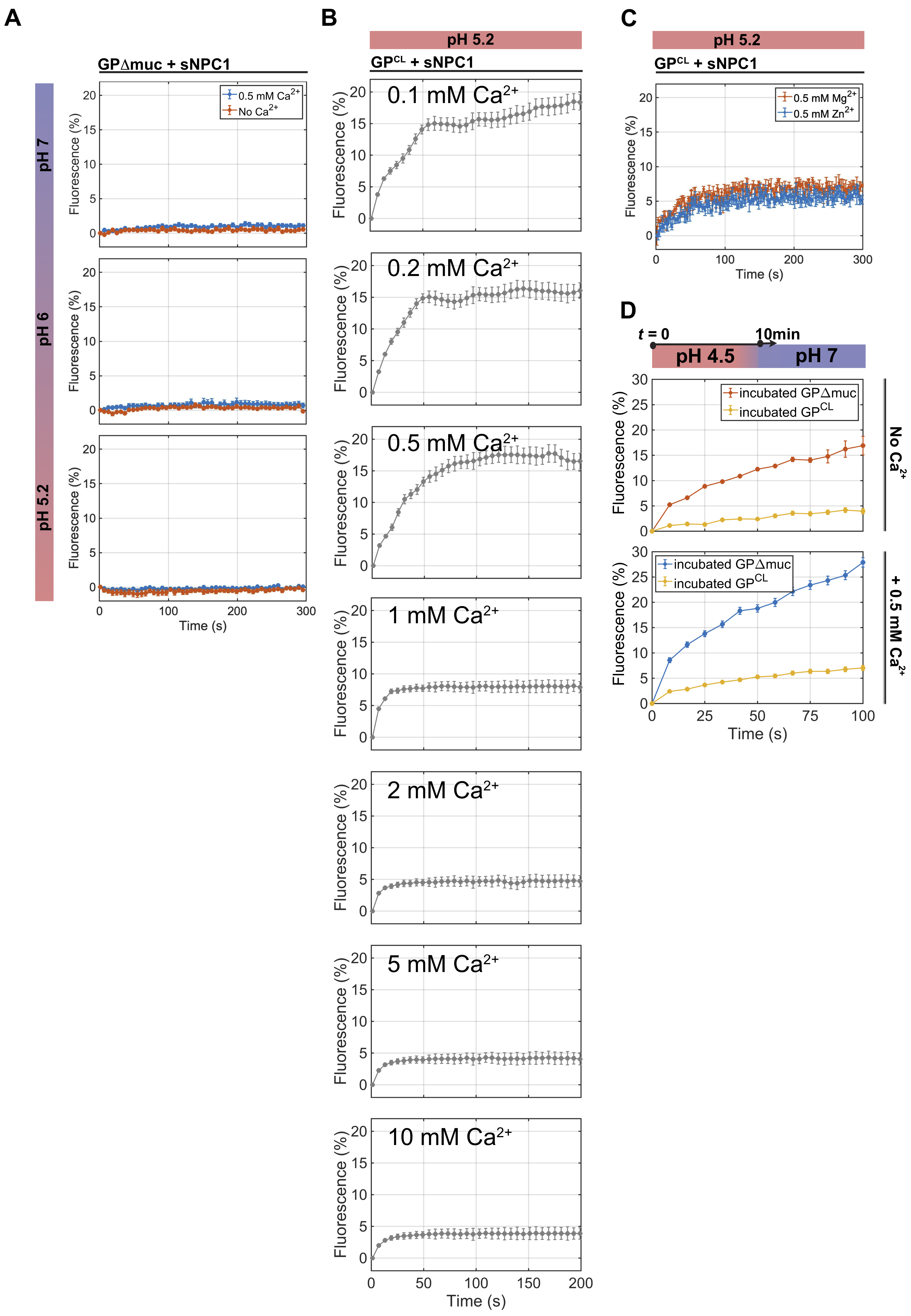

Supplement: S2 Fig — (A) As described in Fig 1, fluorescently labeled GP-containing virions were incubated with liposomes at the indicated pH and in the absence (red) or presence (blue) of Ca2+. No fluorescence dequenching was observed in the presence of NPC1 prior to removal of the glycan cap, regardless of the presence of Ca2+ or the pH. (B) In contrast to the acceleration observed in the presence of Ca2+, only slow dequenching was seen at pH 5.2 with NPC1 in the presence of 0.5 mM MgCl2 or ZnCl2. (C) Equivalent levels of dequenching were observed across a range of physiological Ca2+ concentrations (0.1–0.5 mM) at pH 5.2 with NPC1. However, Ca2+ concentrations of at least 1 mM led to a loss of dequenching. (D, top) Dequenching observed for pseudovirions containing GPΔmuc that were incubated at pH 4.5 for 10 min, followed by removal of the glycan cap with thermolysin. (Bottom) Dequenching observed for pseudovirions that were incubated after glycan cap removal. The same data are presented in the absence and presence of Ca2+, as indicated. In all panels, data are presented as the percentage of maximal dequenching seen upon addition of 1% triton X-100 and as an average of 3 independent measurements, with error bars reflecting the standard deviation. GP, EBOV envelope glycoprotein; GPΔmuc, GP with the mucin-like domain deleted; NPC1, Niemann-Pick C1. (TIF) [file pbio.3000626.s002.tif]

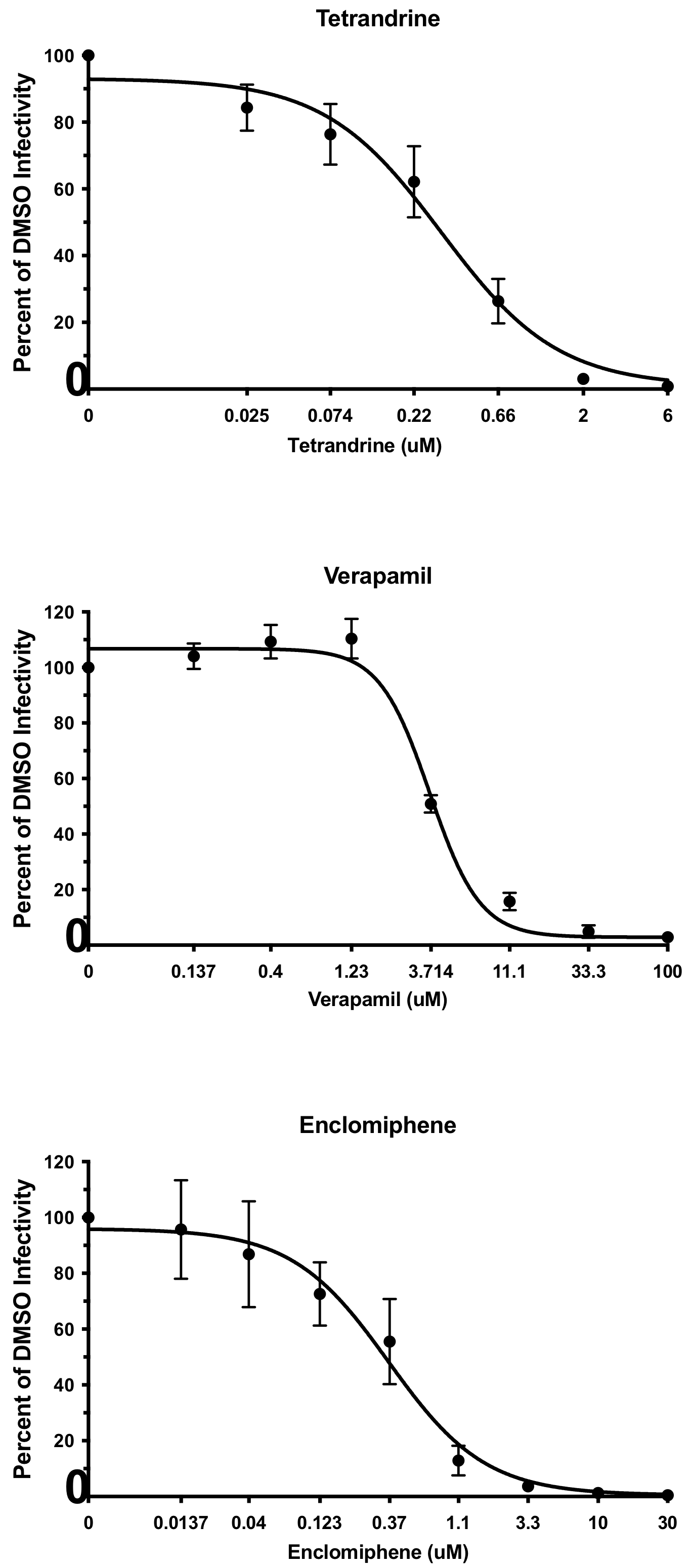

Supplement: S3 Fig — Pseudovirus infectivity was tested in the presence of compounds known to affect endosomal Ca2+ (Materials and methods). Infectivity is presented as a percentage of DMSO control. Data are presented as the average of 3 to 6 independent measurements, with error bars reflecting the standard errors. GP, EBOV envelope glycoprotein. (TIF) [file pbio.3000626.s003.tif]

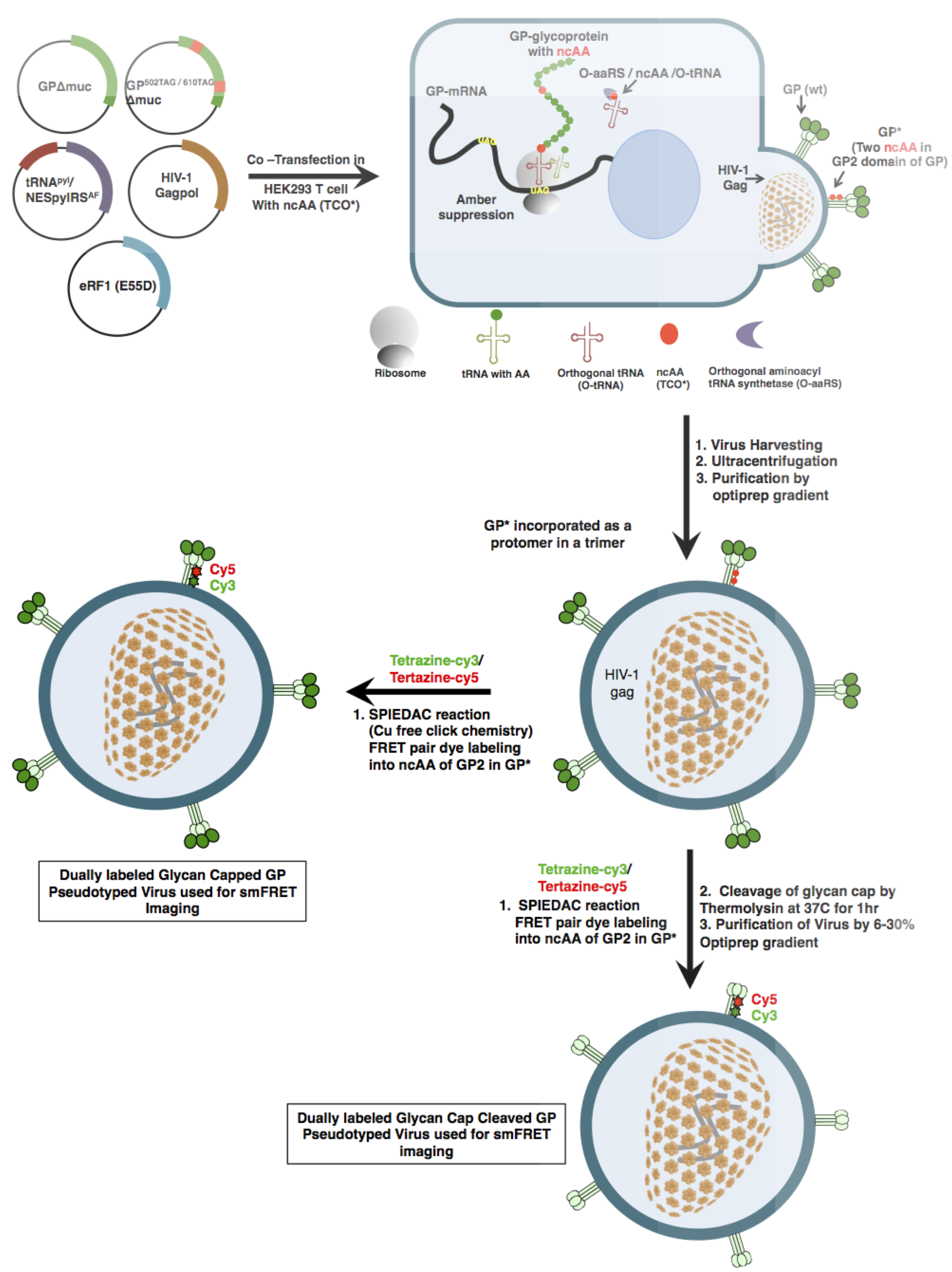

Supplement: S4 Fig — See Materials and methods for details. GP, EBOV envelope glycoprotein. (TIF) [file pbio.3000626.s004.tif]

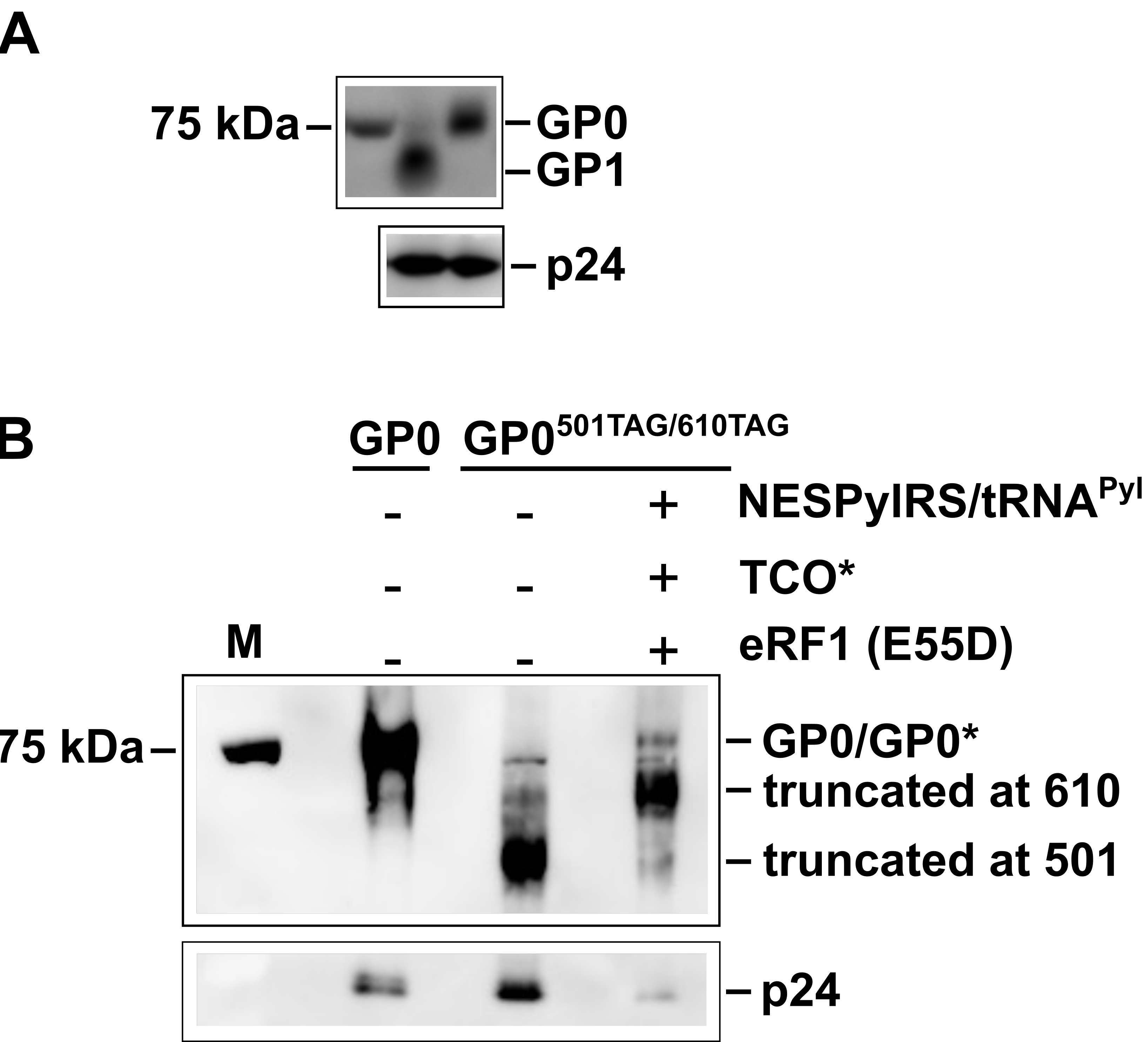

Supplement: S5 Fig — (A) Wild-type GPΔmuc and GP0 efficiently incorporate into virions formed with the HIV core. Western blot demonstrating efficient furin-mediated cleavage of GP0. The HIV capsid protein, p24, serves as a loading control. (B) Translation of full-length GP* requires the presence of NESPylRSAF/tRNAPyl, the TCO* ncAA, and the dominant negative E55D mutant of eRF1. eRF1, eukaryotic release factor 1; GP, EBOV envelope glycoprotein; GPΔmuc, GP with the mucin-like domain deleted; HIV, human immunodeficiency virus; TCO*, trans-cyclooct-2-ene-L-lysine. (TIF) [file pbio.3000626.s005.tif]

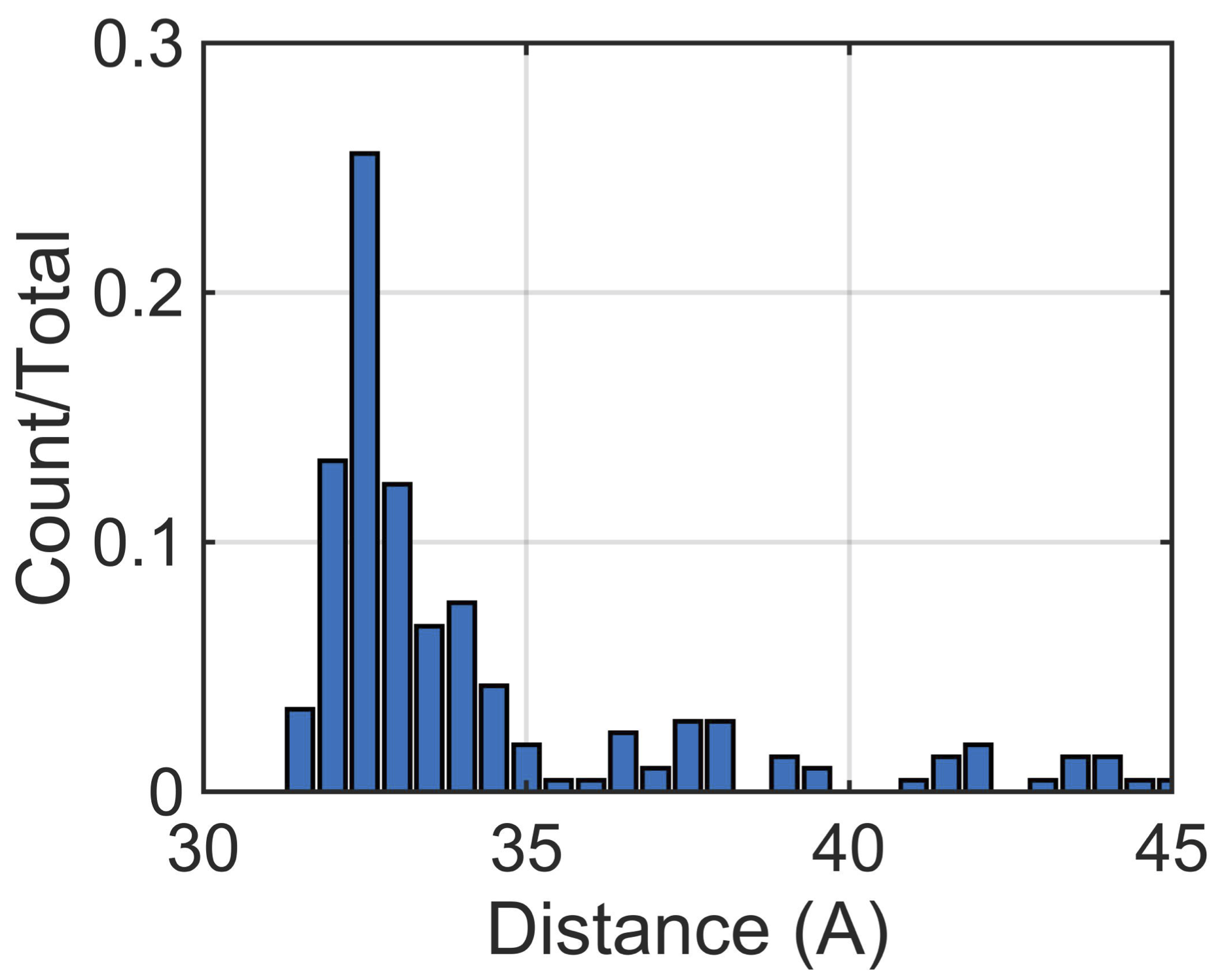

Supplement: S6 Fig — A molecular model of GP*-Cy3-Cy5 was generated based on the prefusion structure of GP (PDB accession code 5JQ3; Materials and methods). The histogram of distances between the centers of mass of the 2 fluorophores is shown. The average interfluorophore distance was 35 ± 4 Å, consistent with the 0.95-FRET state that predominates for GPΔmuc at pH 7. Models of the Cy3 and Cy5 fluorophores are shown with the calculated transition dipoles used to calculate the orientation factor κ2, which is 2/3 for freely tumbling fluorophores. The fluorophore orientations determined in the 50-ns trajectory led to κ 2 = 0.645 ± 0.320, indicating that on average the fluorophores are freely tumbling. Their relative rotational orientations vary considerably over time scales that are far shorter than our experimental time resolution of 40 ms. FRET, Förster resonance energy transfer; GP, EBOV envelope glycoprotein; GPΔmuc, GP with the mucin-like domain deleted; MD, molecular dynamics. (TIF) [file pbio.3000626.s006.tif]

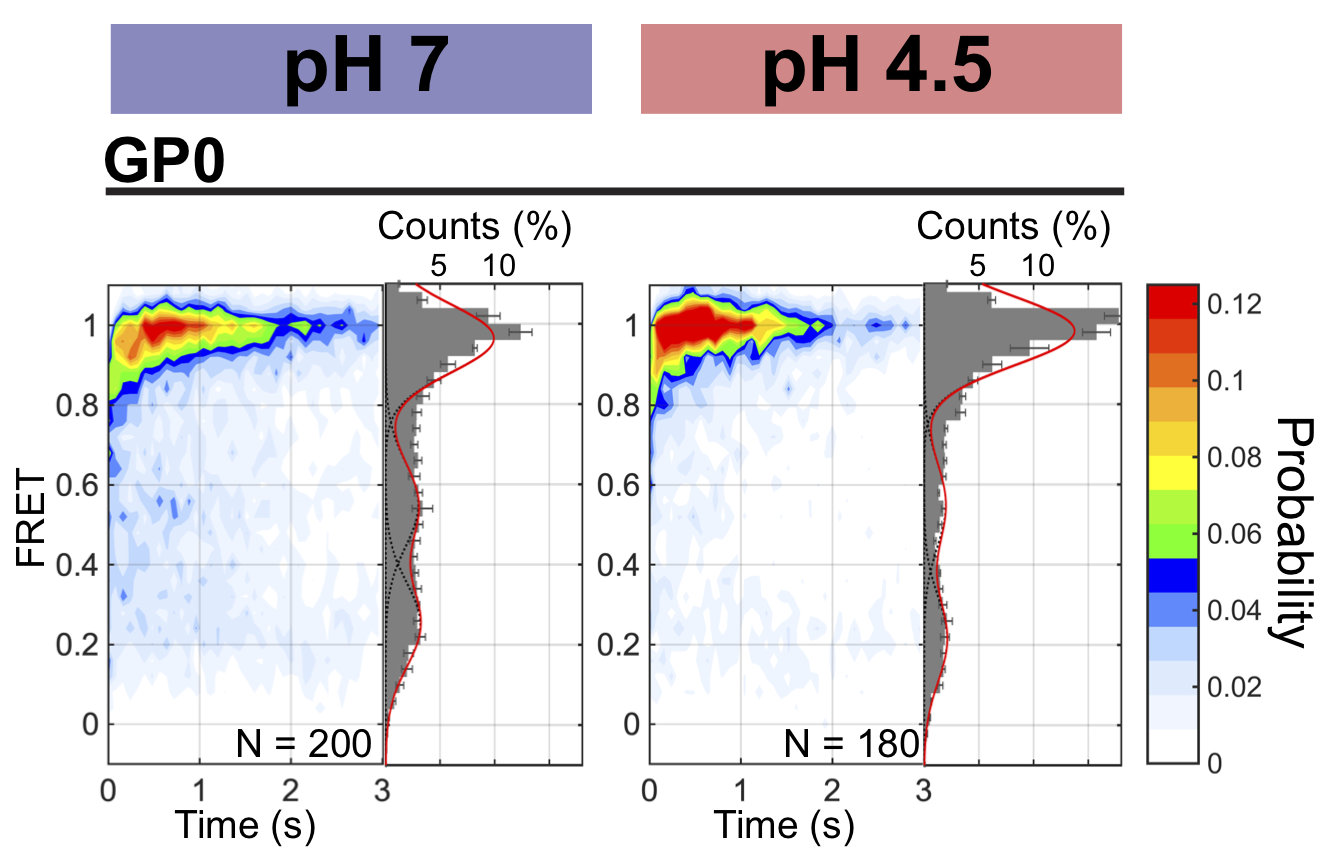

Supplement: S7 Fig — Contour plots and FRET histograms displaying the FRET distribution from the population of individual GP0 molecules at the indicated pH. Histograms are overlaid with Gaussian distributions as in Figs 2–4. N indicates the number of FRET traces compiled into each contour plot and histogram. FRET, Förster resonance energy transfer; GP, EBOV envelope glycoprotein. (TIF) [file pbio.3000626.s007.tif]

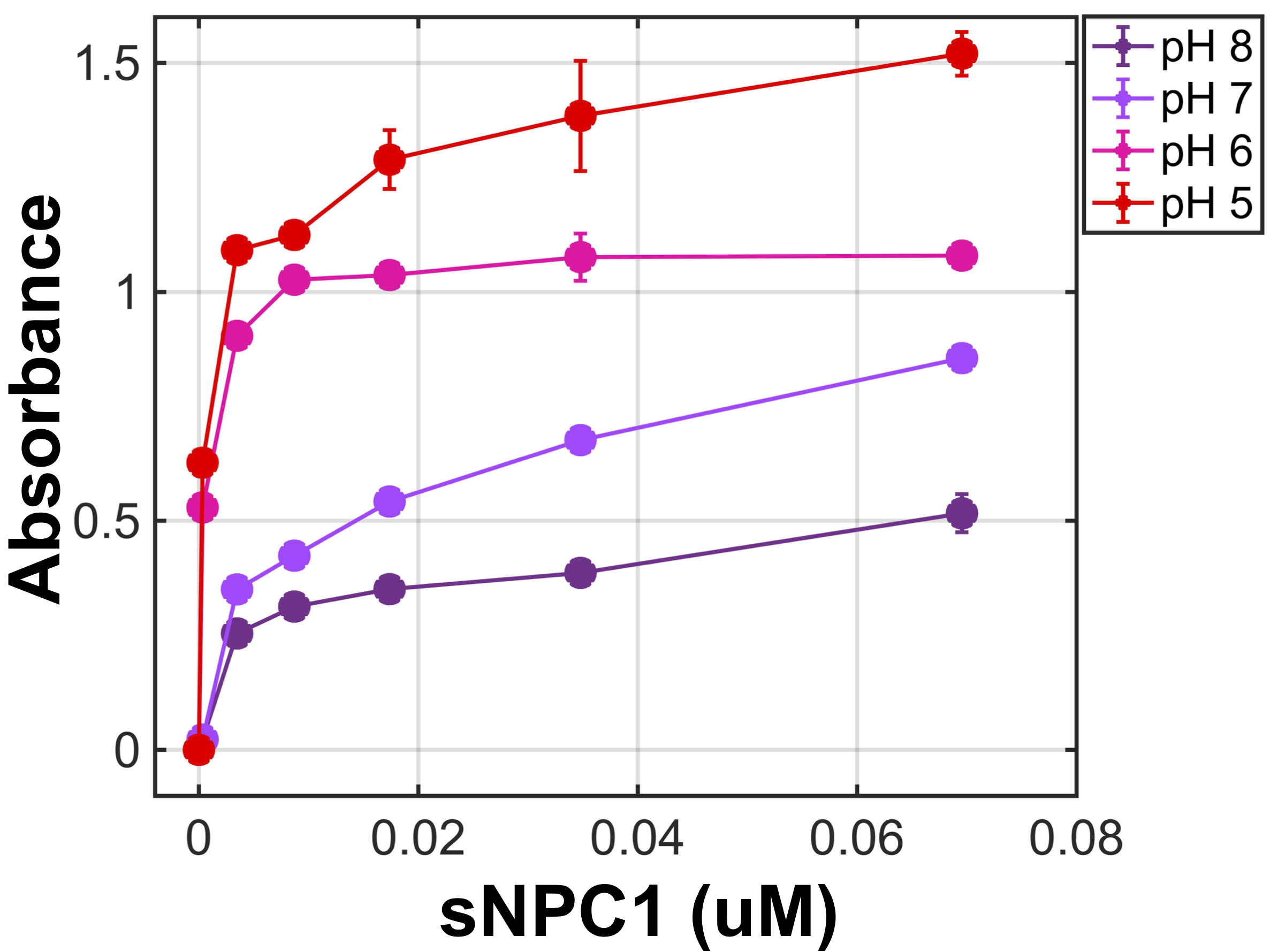

Supplement: S8 Fig — Pseudovirions containing GPCL were incubated with FLAG-tagged sNPC1-C across a range of pHs. Following incubation, excess sNPC1-C was removed with neutral pH buffer. The extent of binding was determined in an ELISA assay using an anti-FLAG antibody conjugated to horseradish peroxidase (Materials and methods). Greater binding is seen at acidic pH, which may be due to acidic pH facilitating transition of GP to a conformation optimal for NPC1 binding. Data are presented as the average of 3 independent measurements, with error bars reflecting the standard error. ELISA, enzyme-linked immunosorbent assay; GP, EBOV envelope glycoprotein; NPC1, Niemann-Pick C1; sNPC1-C, soluble domain C of NPC1. (TIF) [file pbio.3000626.s008.tif]

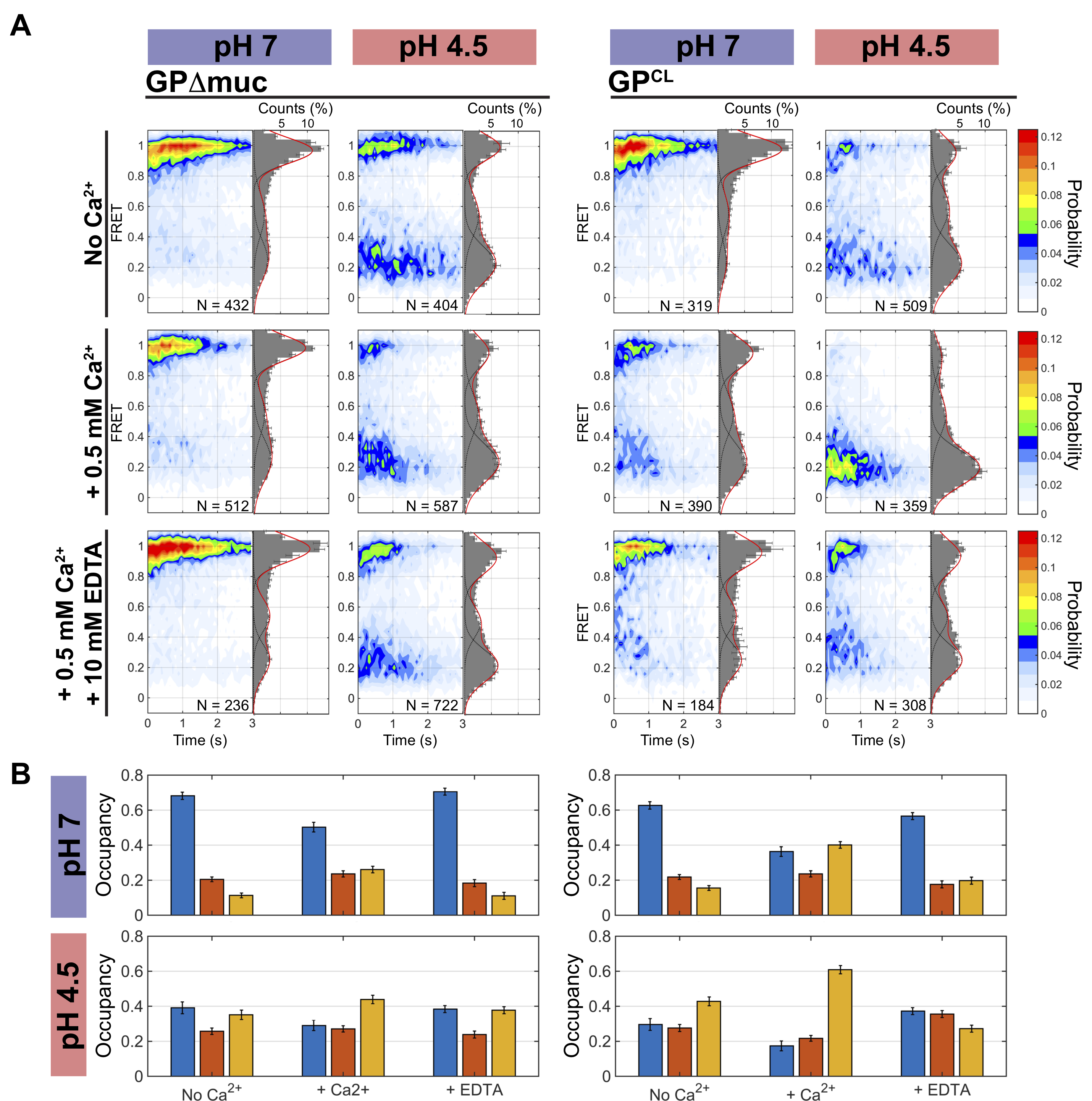

Supplement: S9 Fig — (A) Contour plots and FRET histograms for GPΔmuc and GPCL acquired under the indicated conditions. FRET data are displayed as in Figs 2–4. N indicates the number of FRET traces compiled into each contour plot and histogram. (B) Occupancies in the high- (blue), intermediate- (orange), and low-FRET (yellow) states determined through HMM analysis. Occupancies in the 3 FRET states are normalized such that their sum equals 100%. EDTA, ethylenediaminetetraacetic acid; FRET, Förster resonance energy transfer; GP, EBOV envelope glycoprotein; GPΔmuc, GP with the mucin-like domain deleted; HMM, hidden Markov modeling. (TIF) [file pbio.3000626.s009.tif]

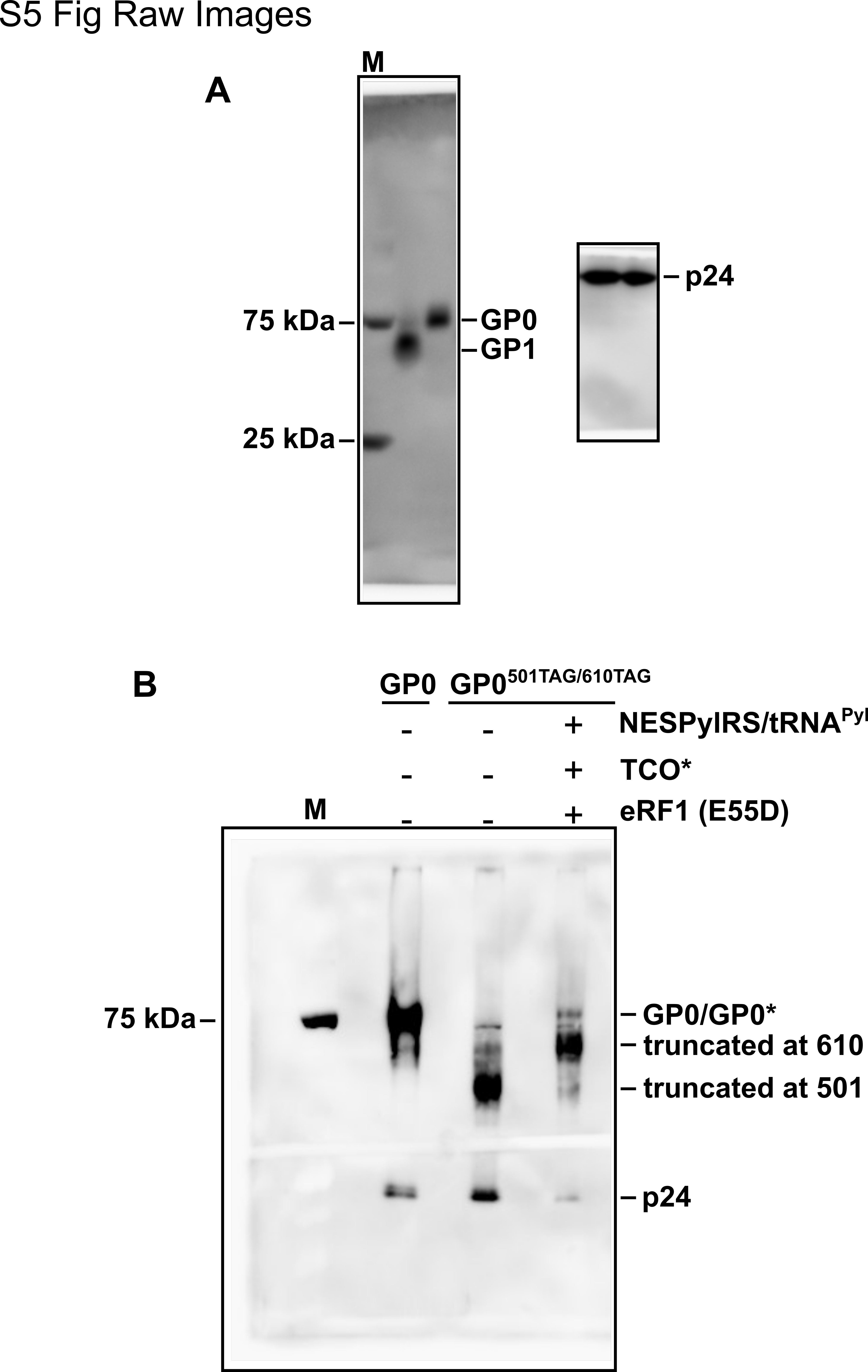

Supplement: S1 Raw Image — (TIF) [file pbio.3000626.s011.tif]
