# Supplementary material for: Conformational changes in the Ebola virus membrane fusion machine induced by pH, Ca2+, and receptor binding
Source: PLoS Biol. 2020 Feb 10;18(2):e3000626. doi: 10.1371/journal.pbio.3000626 (PMC7034923; doi:10.1371/journal.pbio.3000626)
Supplement: S1 Data — (PDF) [file pbio.3000626.s010.pdf]

FIG 1 DATA. Fluorescence dequenching data plotted in Fig 1. Four replicates for each experiment are provided (rep1-4), along with the mean and standard deviation. Columns are colored according to the plots in Fig 1.

pH7

	**Neither**						**sNPC1-C**						**sNPC1-C + Ca2+**					
**time**	**rep1**	**rep2**	**rep3**	**rep4**	**mean**	**error bars**	**rep1**	**rep2**	**rep3**	**rep4**	**mean**	**error bars**	**rep1**	**rep2**	**rep3**	**rep4**	**mean**	**error bars**
0	0	0	0	0	0.0000	0.0000	0	0	0	0	0.0000	0.0000	0	0	0	0	**0.0000**	**0.0000**
6	-0.0022	0.0001	0.0057	0.0103	0.0035	0.0056	-0.0035	-0.0046	0.0021	0.009	0.0008	0.0062	0.0113	0.0048	0.0218	0.023	**0.0152**	**0.0087**
12	-0.0112	-0.0092	0.0033	-0.0033	-0.0051	0.0065	-0.0142	-0.0116	-0.0009	-0.0035	-0.0076	0.0064	0.0111	0.017	0.0214	0.0207	**0.0176**	**0.0047**
18	-0.0076	-0.0032	0.0007	0.0076	-0.0006	0.0064	-0.0068	-0.0111	-0.0036	0.0001	-0.0054	0.0048	0.0178	0.0196	0.034	0.0259	**0.0243**	**0.0073**
24	0.0007	0.0017	0.0091	0.0139	0.0064	0.0063	-0.0061	-0.0061	-0.0025	-0.0044	-0.0048	0.0017	0.0217	0.0305	0.0385	0.04	**0.0327**	**0.0084**
30	-0.0062	-0.0063	0.0042	0.0078	-0.0001	0.0072	-0.0092	-0.013	-0.0029	-0.0015	-0.0067	0.0054	0.0219	0.0264	0.0334	0.0339	**0.0289**	**0.0058**
36	-0.0094	-0.0062	-0.0033	-0.0055	-0.0061	0.0025	-0.0097	-0.0123	0.0009	0.0019	-0.0048	0.0072	0.0261	0.033	0.0384	0.0479	**0.0364**	**0.0092**
42	-0.0086	-0.007	0.0004	0.0032	-0.0030	0.0057	-0.0033	-0.011	0.0075	0.0065	-0.0001	0.0088	0.0341	0.028	0.04	0.0468	**0.0372**	**0.0080**
48	-0.0044	0.0025	0.004	0.0108	0.0032	0.0062	-0.0001	0.0008	0.0102	0.0182	0.0073	0.0086	0.0304	0.0246	0.041	0.0423	**0.0346**	**0.0085**
54	-0.0101	-0.0073	-0.0001	0.0044	-0.0033	0.0066	-0.0069	-0.0103	0.0033	-0.0005	-0.0036	0.0061	0.0288	0.0261	0.0433	0.0359	**0.0335**	**0.0077**
60	-0.0104	-0.0111	-0.0059	-0.0049	-0.0081	0.0031	-0.0036	0.0005	0.0071	0.0157	0.0049	0.0084	0.0311	0.0346	0.0406	0.049	**0.0388**	**0.0078**
66	-0.0064	-0.0071	0.0022	0.0001	-0.0028	0.0046	0.004	0.0009	0.0114	0.0132	0.0074	0.0059	0.0331	0.0421	0.0526	0.0507	**0.0446**	**0.0089**
72	-0.0049	-0.0052	-0.0003	0.0039	-0.0016	0.0043	-0.0034	0.0006	0.0072	0.0121	0.0041	0.0069	0.0259	0.0297	0.0416	0.0466	**0.0360**	**0.0098**
78	-0.0148	-0.0124	-0.0027	-0.0035	-0.0084	0.0061	-0.0124	-0.0144	0.0017	0.009	-0.0040	0.0113	0.0288	0.032	0.046	0.0509	**0.0394**	**0.0107**
84	-0.0165	-0.0145	-0.0042	-0.0029	-0.0095	0.0070	-0.0086	-0.013	0.003	0.0024	-0.0041	0.0080	0.0206	0.0298	0.0394	0.0434	**0.0333**	**0.0102**
90	-0.0137	-0.0062	-0.0023	-0.0037	-0.0065	0.0051	0.0113	0.0074	0.0203	0.0176	0.0142	0.0059	0.033	0.0393	0.0489	0.0543	**0.0439**	**0.0095**
96	-0.0083	-0.0042	-0.0024	-0.0018	-0.0042	0.0029	0.0021	-0.004	0.007	0.0114	0.0041	0.0066	0.0264	0.029	0.0386	0.0408	**0.0337**	**0.0071**
102	-0.0089	-0.0104	0.0024	0.0044	-0.0031	0.0076	0.0013	0.0005	0.0053	0.0146	0.0054	0.0065	0.0348	0.0297	0.0489	0.0521	**0.0414**	**0.0108**
108	-0.0089	-0.012	-0.0059	0	-0.0067	0.0051	-0.0032	-0.0046	0.0034	0.0134	0.0023	0.0082	0.0269	0.0277	0.0364	0.0297	**0.0302**	**0.0043**
114	-0.0121	-0.0061	0.0016	0.0047	-0.0030	0.0076	0.0057	-0.0004	0.0112	0.012	0.0071	0.0057	0.0221	0.0268	0.0408	0.0332	**0.0307**	**0.0081**
120	-0.008	-0.0069	-0.0027	-0.0031	-0.0052	0.0027	0.0014	0	0.0113	0.0103	0.0058	0.0059	0.0256	0.0362	0.0439	0.0399	**0.0364**	**0.0079**
126	-0.0049	-0.004	-0.0007	-0.0013	-0.0027	0.0020	0.0041	0.0027	0.0109	0.012	0.0074	0.0047	0.0312	0.028	0.038	0.0435	**0.0352**	**0.0069**
132	-0.015	-0.0093	-0.0069	-0.0065	-0.0094	0.0039	0.0038	0.0015	0.0085	0.0062	0.0050	0.0030	0.0382	0.0396	0.0481	0.0521	**0.0445**	**0.0067**
138	-0.0058	-0.0012	0.0048	0.0055	0.0008	0.0053	0.0069	0.0016	0.014	0.0146	0.0093	0.0062	0.03	0.0262	0.0433	0.046	**0.0364**	**0.0097**
144	-0.0136	-0.0084	-0.005	-0.0043	-0.0078	0.0042	0.0047	0.0055	0.0164	0.0156	0.0106	0.0063	0.0352	0.0294	0.0443	0.0384	**0.0368**	**0.0062**
150	-0.0083	-0.0069	-0.0056	0.0031	-0.0044	0.0051	0.0078	0.0047	0.0159	0.0118	0.0101	0.0049	0.0318	0.0273	0.0436	0.042	**0.0362**	**0.0079**
156	-0.004	-0.0007	0.0065	0.0026	0.0011	0.0045	-0.0028	-0.007	0.0082	0.0032	0.0004	0.0067	0.0261	0.0318	0.0401	0.0406	**0.0347**	**0.0070**
162	-0.0109	-0.0061	-0.003	-0.0049	-0.0062	0.0034	0.0058	0.0005	0.0185	0.0224	0.0118	0.0103	0.0318	0.0357	0.0437	0.046	**0.0393**	**0.0067**
168	-0.004	-0.0088	0.0015	0.0026	-0.0022	0.0053	0.0069	0.0031	0.0104	0.0178	0.0096	0.0063	0.0277	0.032	0.0411	0.0444	**0.0363**	**0.0078**
174	-0.0057	-0.003	0.0002	0.0054	-0.0008	0.0048	-0.0013	-0.0017	0.0052	0.0103	0.0031	0.0057	0.0243	0.0294	0.0391	0.0468	**0.0349**	**0.0100**
180	-0.0035	-0.0074	0.003	0.0019	-0.0015	0.0049	-0.0012	0.0011	0.0086	0.015	0.0059	0.0074	0.0346	0.0381	0.0494	0.0467	**0.0422**	**0.0070**
186	-0.0132	-0.0099	-0.0071	-0.0055	-0.0089	0.0034	0.0048	0.0057	0.0068	0.0074	0.0062	0.0012	0.0275	0.0326	0.042	0.0482	**0.0376**	**0.0093**
192	-0.0021	0	0.0064	0.0037	0.0020	0.0038	0.0012	0.0038	0.0121	0.0176	0.0087	0.0076	0.0198	0.0276	0.0322	0.0296	**0.0273**	**0.0053**
198	-0.0163	-0.0111	-0.0035	-0.0067	-0.0094	0.0056	0.0055	0.0021	0.0108	0.0162	0.0087	0.0062	0.0285	0.0402	0.0433	0.0576	**0.0424**	**0.0120**
204	-0.015	-0.013	-0.0031	-0.003	-0.0085	0.0064	0.0108	0.0045	0.0204	0.0207	0.0141	0.0079	0.0329	0.0351	0.0375	0.0391	**0.0362**	**0.0027**
210	-0.0095	-0.0115	-0.0008	0.0029	-0.0047	0.0069	0.0097	-0.001	0.0158	0.0142	0.0097	0.0076	0.0313	0.04	0.0496	0.043	**0.0410**	**0.0076**
216	-0.0106	-0.0101	-0.0039	-0.0001	-0.0062	0.0051	0.0086	0.0005	0.0163	0.021	0.0116	0.0090	0.0369	0.0326	0.0482	0.0469	**0.0412**	**0.0076**
222	-0.0142	-0.013	-0.0043	-0.0074	-0.0097	0.0047	0.0105	0.0052	0.0169	0.0153	0.0120	0.0053	0.0216	0.0259	0.0349	0.0347	**0.0293**	**0.0066**
228	-0.0104	-0.0094	0.0004	0.0004	-0.0048	0.0060	0.007	0.0072	0.0166	0.0122	0.0108	0.0046	0.0334	0.0383	0.0533	0.0512	**0.0441**	**0.0097**
234	-0.0073	-0.0026	0.0013	-0.0017	-0.0026	0.0036	0.0141	0.0048	0.0193	0.0178	0.0140	0.0065	0.0361	0.0357	0.0375	0.0422	**0.0379**	**0.0030**
240	-0.013	-0.0103	-0.0064	-0.0032	-0.0082	0.0043	0.0065	-0.0027	0.0104	0.0194	0.0084	0.0092	0.0357	0.031	0.0439	0.0538	**0.0411**	**0.0100**
246	-0.0068	-0.0044	-0.0024	0.0002	-0.0034	0.0030	0.0048	0.0014	0.0126	0.0123	0.0078	0.0056	0.0354	0.0325	0.0426	0.0526	**0.0408**	**0.0090**
252	-0.0127	-0.0102	-0.0059	0.0011	-0.0069	0.0060	0.011	0.0035	0.0183	0.0166	0.0124	0.0067	0.0276	0.0341	0.0452	0.0384	**0.0363**	**0.0074**
258	-0.0132	-0.012	-0.0057	0.0009	-0.0075	0.0065	0.0068	0.0104	0.014	0.0125	0.0109	0.0031	0.0249	0.0324	0.0455	0.0486	**0.0379**	**0.0111**
264	-0.0148	-0.0123	-0.0021	-0.0026	-0.0080	0.0065	0.0042	0.0032	0.0126	0.0181	0.0095	0.0071	0.0337	0.0349	0.0408	0.0429	**0.0381**	**0.0045**
270	-0.0122	-0.012	0.0004	0.0028	-0.0053	0.0080	0.0139	0.0113	0.0238	0.0177	0.0167	0.0054	0.0231	0.0341	0.0398	0.0412	**0.0346**	**0.0082**
276	-0.0105	-0.0142	-0.0101	-0.0089	-0.0109	0.0023	0.001	0.004	0.0088	0.0188	0.0082	0.0078	0.0304	0.0382	0.045	0.0488	**0.0406**	**0.0081**
282	-0.0132	-0.0081	-0.004	-0.0037	-0.0073	0.0044	0.0102	0.0152	0.0202	0.0191	0.0162	0.0045	0.0381	0.037	0.0447	0.0524	**0.0431**	**0.0071**
288	-0.0137	-0.0162	-0.0095	-0.0008	-0.0101	0.0068	0.0125	0.0052	0.0219	0.0174	0.0143	0.0072	0.0404	0.0476	0.0565	0.0557	**0.0501**	**0.0076**
294	-0.0066	-0.0083	0.0024	0.0062	-0.0016	0.0070	0.0106	0.0137	0.019	0.0201	0.0159	0.0045	0.0361	0.0395	0.0494	0.0566	**0.0454**	**0.0094**

pH6

	Neither						sNPC1-C						sNPC1-C + Ca^{2+}					
time	rep1	rep2	rep3	rep4	mean	error bars	rep1	rep2	rep3	rep4	mean	error bars	rep1	rep2	rep3	rep4	mean	error bars
0	0	0	0	0	0.0000	0.0000	0	0	0	0	0.0000	0.0000	0	0	0	0	0.0000	0.0000
6	-0.0028	-0.0033	0.0052	0.0073	0.0016	0.0054	-0.012	-0.0075	-0.0016	0.0087	-0.0031	0.0089	0.0038	0.0059	0.0282	0.01	0.0120	0.0111
12	-0.0084	-0.0081	0.0036	0.0091	-0.0010	0.0087	-0.01	-0.0232	-0.0039	-0.0052	-0.0106	0.0088	0.0126	0.0283	0.0302	0.03	0.0253	0.0085
18	-0.0066	-0.008	0.0009	0.0002	-0.0034	0.0046	-0.015	-0.0067	-0.0028	0.0022	-0.0056	0.0073	0.0167	0.0281	0.0435	0.027	0.0288	0.0110
24	-0.01	-0.0112	-0.0066	-0.0039	-0.0079	0.0033	-0.0188	-0.0152	-0.0055	-0.0058	-0.0113	0.0067	0.0325	0.0368	0.0528	0.0388	0.0402	0.0088
30	-0.0148	-0.0134	-0.0054	0.0024	-0.0078	0.0080	-0.0133	-0.011	0.0024	-0.0006	-0.0056	0.0077	0.0328	0.0459	0.048	0.0498	0.0441	0.0077
36	-0.0137	-0.0142	-0.0086	-0.0108	-0.0118	0.0026	-0.0093	-0.0139	-0.006	0.0001	-0.0073	0.0059	0.0339	0.0397	0.0532	0.0513	0.0445	0.0093
42	-0.0205	-0.0191	-0.0062	0.0003	-0.0114	0.0101	0.0028	0.006	0.0133	0.0123	0.0086	0.0050	0.0349	0.0426	0.0605	0.0503	0.0471	0.0109
48	-0.0153	-0.0119	-0.0012	-0.0052	-0.0084	0.0064	-0.0037	0.0053	0.0119	0.0176	0.0078	0.0092	0.0506	0.0429	0.0665	0.0503	0.0526	0.0099
54	-0.0073	-0.0087	0.0017	0.0049	-0.0024	0.0067	-0.0084	-0.0043	0.0062	0.0029	-0.0009	0.0066	0.0399	0.0533	0.0704	0.0537	0.0543	0.0125
60	-0.0118	-0.0164	-0.009	-0.0038	-0.0103	0.0053	-0.004	-0.0035	0.0052	0.0122	0.0025	0.0077	0.0544	0.0576	0.0672	0.0595	0.0597	0.0054
66	-0.0163	-0.013	-0.0076	-0.0017	-0.0097	0.0064	0.0047	-0.0014	0.0102	0.0155	0.0073	0.0073	0.0415	0.0526	0.0742	0.054	0.0556	0.0136
72	-0.0106	-0.0071	-0.002	0.0011	-0.0047	0.0052	-0.0089	-0.0049	0.0062	0.0095	0.0005	0.0088	0.0464	0.0446	0.0712	0.0532	0.0539	0.0121
78	-0.0174	-0.013	-0.004	-0.001	-0.0089	0.0076	-0.0033	-0.0081	0.0112	0.0164	0.0041	0.0116	0.0508	0.0519	0.0669	0.054	0.0559	0.0075
84	-0.0066	-0.0071	-0.0002	0.0047	-0.0023	0.0056	0.0042	0.0027	0.0197	0.0163	0.0107	0.0085	0.04	0.0447	0.0667	0.0557	0.0518	0.0119
90	-0.009	-0.009	0.0009	-0.005	-0.0055	0.0047	-0.0019	-0.0063	0.002	0.0068	0.0002	0.0056	0.0619	0.0611	0.068	0.0604	0.0629	0.0035
96	-0.0103	-0.0101	-0.0017	-0.0044	-0.0066	0.0043	0.0075	0.0075	0.0129	0.0161	0.0110	0.0042	0.0504	0.0544	0.0658	0.0538	0.0561	0.0067
102	-0.0079	-0.005	0.0019	0.0109	0.0000	0.0084	0.0075	0.0058	0.0216	0.0287	0.0159	0.0111	0.0493	0.0553	0.0795	0.0587	0.0607	0.0131
108	-0.0132	-0.0128	-0.0062	0.0014	-0.0077	0.0069	0.0037	0.0104	0.0196	0.0248	0.0146	0.0094	0.053	0.0623	0.0758	0.0577	0.0622	0.0098
114	-0.0111	-0.0105	0.0004	-0.0026	-0.0060	0.0057	0.0024	0.0025	0.0198	0.0162	0.0102	0.0091	0.0513	0.0645	0.0814	0.0629	0.0650	0.0124
120	-0.01	-0.0064	-0.001	0.0014	-0.0040	0.0052	0.0047	0.0031	0.0127	0.0085	0.0073	0.0043	0.0551	0.0661	0.0784	0.0636	0.0658	0.0096
126	-0.0091	-0.009	-0.0046	-0.0043	-0.0068	0.0027	0.0092	0.0067	0.0147	0.0164	0.0118	0.0046	0.0578	0.0637	0.0682	0.0709	0.0652	0.0057
132	-0.0088	-0.0065	0.0021	-0.0004	-0.0034	0.0051	0.0137	0.0139	0.0168	0.0158	0.0151	0.0015	0.0707	0.0658	0.089	0.0723	0.0745	0.0101
138	-0.006	-0.0102	0	-0.0016	-0.0045	0.0046	0.007	0.005	0.0143	0.0205	0.0117	0.0071	0.0538	0.0562	0.0805	0.0595	0.0625	0.0122
144	-0.0029	-0.0073	0.0035	0.002	-0.0012	0.0049	0.0098	0.01	0.0188	0.0169	0.0139	0.0047	0.0621	0.0635	0.0748	0.0665	0.0667	0.0057
150	-0.0128	-0.0075	-0.0028	-0.0026	-0.0064	0.0048	-0.0089	0.0025	0.0048	0.0054	0.0010	0.0067	0.0544	0.0609	0.0775	0.0688	0.0654	0.0100
156	-0.0052	-0.0074	0.0017	0.0018	-0.0023	0.0047	0.0179	0.0096	0.0224	0.022	0.0180	0.0059	0.0566	0.0622	0.08	0.0652	0.0660	0.0100
162	-0.0081	-0.0095	0.0013	-0.0015	-0.0045	0.0052	0.0093	0.0052	0.0144	0.0219	0.0127	0.0072	0.0517	0.0597	0.0704	0.0576	0.0599	0.0078
168	-0.0134	-0.0106	-0.0046	0.0064	-0.0056	0.0088	0.0086	0.0072	0.0179	0.025	0.0147	0.0084	0.0572	0.0651	0.083	0.0676	0.0682	0.0108
174	-0.0066	-0.0098	-0.0024	-0.0038	-0.0057	0.0033	0.013	0.0182	0.0286	0.0295	0.0223	0.0081	0.0567	0.0664	0.0759	0.0703	0.0673	0.0081
180	-0.0089	-0.008	-0.0055	0.0051	-0.0043	0.0064	0.0163	0.0121	0.0245	0.0326	0.0214	0.0091	0.0628	0.0729	0.0874	0.075	0.0745	0.0101
186	-0.0105	-0.0102	-0.0029	-0.0023	-0.0065	0.0045	0.0109	0.013	0.0145	0.0146	0.0133	0.0017	0.0571	0.0625	0.0673	0.0623	0.0623	0.0042
192	-0.0136	-0.0098	-0.004	-0.0052	-0.0082	0.0044	0.0239	0.0163	0.035	0.0384	0.0284	0.0102	0.0569	0.0729	0.077	0.0719	0.0697	0.0088
198	-0.0092	-0.009	0.0001	-0.0013	-0.0049	0.0049	0.0108	0.0029	0.0144	0.0234	0.0129	0.0085	0.0657	0.0646	0.0762	0.0762	0.0707	0.0064
204	-0.0029	-0.0009	0.0049	0.0092	0.0026	0.0055	0.0168	0.0136	0.026	0.0311	0.0219	0.0081	0.0714	0.0704	0.0733	0.0667	0.0705	0.0028
210	-0.006	-0.0062	-0.0021	0.0059	-0.0021	0.0057	0.0094	0.0107	0.0235	0.024	0.0169	0.0079	0.0748	0.0763	0.0905	0.0707	0.0781	0.0086
216	-0.0109	-0.0132	-0.0043	-0.0004	-0.0072	0.0059	0.0118	0.0151	0.0246	0.0281	0.0199	0.0077	0.0558	0.0666	0.0818	0.0662	0.0676	0.0107
222	-0.0119	-0.0088	-0.0041	0.0004	-0.0061	0.0054	0.0186	0.0117	0.0207	0.0239	0.0187	0.0052	0.0707	0.0771	0.084	0.075	0.0767	0.0055
228	-0.0056	-0.0103	0.0011	0.0074	-0.0019	0.0077	0.014	0.0116	0.0226	0.021	0.0173	0.0053	0.0661	0.0752	0.096	0.0776	0.0787	0.0125
234	-0.0083	-0.0052	-0.0006	-0.0039	-0.0045	0.0032	0.0162	0.0098	0.0178	0.0197	0.0159	0.0043	0.0777	0.0779	0.0817	0.0796	0.0792	0.0019
240	-0.0062	-0.0099	-0.0016	-0.0014	-0.0048	0.0041	0.0151	0.0181	0.0227	0.0337	0.0224	0.0082	0.0622	0.061	0.0787	0.0649	0.0667	0.0082
246	-0.0157	-0.0139	-0.0084	-0.0099	-0.0120	0.0034	0.0173	0.0197	0.0296	0.024	0.0227	0.0054	0.0736	0.0741	0.0905	0.0796	0.0795	0.0079
252	-0.0038	-0.0051	-0.0006	-0.0011	-0.0027	0.0022	0.0015	0.0087	0.0172	0.018	0.0114	0.0078	0.079	0.0796	0.0922	0.0813	0.0830	0.0062
258	-0.009	-0.008	0.0005	0.0054	-0.0028	0.0069	0.0062	0.014	0.0221	0.0178	0.0150	0.0068	0.0662	0.0686	0.0897	0.0693	0.0735	0.0109
264	-0.0126	-0.0068	-0.0001	0.0036	-0.0040	0.0072	0.0134	0.0091	0.0279	0.0257	0.0190	0.0092	0.074	0.0776	0.0832	0.0834	0.0796	0.0046
270	-0.0087	-0.0112	-0.0019	0.005	-0.0042	0.0073	0.0145	0.0133	0.03	0.0266	0.0211	0.0084	0.0765	0.073	0.0854	0.0783	0.0783	0.0052
276	-0.0111	-0.0114	-0.0073	0.0004	-0.0074	0.0055	0.0194	0.0208	0.027	0.0332	0.0251	0.0063	0.0685	0.071	0.0841	0.0748	0.0746	0.0068
282	-0.0149	-0.0105	-0.0057	-0.0092	-0.0101	0.0038	0.0249	0.023	0.0364	0.0327	0.0293	0.0064	0.0745	0.0674	0.0885	0.077	0.0769	0.0088
288	-0.0174	-0.0173	-0.014	-0.0046	-0.0133	0.0060	0.0112	0.0224	0.0271	0.0248	0.0214	0.0070	0.0738	0.0717	0.0877	0.0711	0.0761	0.0078
294	-0.0158	-0.0127	-0.0087	-0.001	-0.0096	0.0064	0.0173	0.0189	0.035	0.0336	0.0262	0.0094	0.0729	0.0655	0.0889	0.0741	0.0754	0.0098

pH5.2

	Neither						sNPC1-C						sNPC1-C + Ca2+					
time	rep1	rep2	rep3	rep4	mean	error bars	rep1	rep2	rep3	rep4	mean	error bars	rep1	rep2	rep3	rep4	mean	error bars
0	0	0	0	0	0.0000	0.0000	0	0	0	0	0.0000	0.0000	0	0	0	0	0.0000	0.0000
6	-0.0016	0.0049	0.0081	-0.003	0.0021	0.0053	0.0162	0.0212	0.0221	0.0243	0.0210	0.0034	0.0151	0.0168	0.0152	0.0096	0.0142	0.0031
12	0.0046	0.0084	0.0119	-0.0002	0.0062	0.0052	0.0254	0.0326	0.0411	0.045	0.0360	0.0088	0.0164	0.02	0.0217	0.0176	0.0189	0.0024
18	-0.001	0.0073	0.0155	0.0059	0.0069	0.0068	0.0333	0.0445	0.0492	0.0524	0.0449	0.0084	0.0441	0.05	0.0515	0.0508	0.0491	0.0034
24	0.0109	0.0066	0.0113	0.0046	0.0084	0.0033	0.0259	0.038	0.0391	0.0412	0.0361	0.0069	0.0491	0.0514	0.0573	0.0515	0.0523	0.0035
30	0.0083	0.0146	0.0132	0.0043	0.0101	0.0047	0.0189	0.0341	0.0274	0.0311	0.0279	0.0066	0.0548	0.0635	0.0682	0.0612	0.0619	0.0056
36	0.0083	0.0183	0.0146	0.0109	0.0130	0.0044	0.0207	0.0383	0.0338	0.0324	0.0313	0.0075	0.0558	0.061	0.0704	0.0702	0.0644	0.0072
42	0.014	0.013	0.0159	0.0127	0.0139	0.0014	0.0242	0.0448	0.044	0.0491	0.0405	0.0111	0.0705	0.0757	0.0872	0.0859	0.0798	0.0081
48	0.0006	0.0146	0.0138	0.0113	0.0101	0.0065	0.0269	0.0449	0.0478	0.0528	0.0431	0.0113	0.0764	0.0799	0.0936	0.0919	0.0855	0.0086
54	-0.0026	0.0111	0.0116	-0.0035	0.0042	0.0083	0.0289	0.0399	0.0536	0.0539	0.0441	0.0120	0.086	0.0967	0.1088	0.1089	0.1001	0.0110
60	0.0059	0.0119	0.015	-0.0004	0.0081	0.0068	0.028	0.0509	0.0536	0.0514	0.0460	0.0120	0.1019	0.1111	0.1243	0.1246	0.1155	0.0110
66	0.0004	0.0135	0.0144	-0.0035	0.0062	0.0091	0.0305	0.0421	0.0509	0.0469	0.0426	0.0088	0.113	0.1243	0.1388	0.1422	0.1296	0.0135
72	0.0121	0.0103	0.0138	0.0065	0.0107	0.0031	0.0414	0.0523	0.0631	0.0655	0.0556	0.0111	0.1041	0.1128	0.128	0.1249	0.1175	0.0111
78	0.0033	0.0085	0.0149	-0.0019	0.0062	0.0072	0.0366	0.0542	0.0591	0.056	0.0515	0.0101	0.1113	0.1208	0.1334	0.1363	0.1255	0.0116
84	0.0092	0.0107	0.0097	0.0026	0.0081	0.0037	0.0425	0.0534	0.0598	0.0618	0.0544	0.0087	0.1112	0.1231	0.1355	0.1375	0.1268	0.0122
90	0.0066	0.0127	0.0095	0.0092	0.0095	0.0025	0.0445	0.0552	0.066	0.0694	0.0588	0.0113	0.1222	0.1347	0.1507	0.1492	0.1392	0.0134
96	0.001	0.0089	0.0116	0.0051	0.0067	0.0046	0.0499	0.0709	0.0759	0.0828	0.0699	0.0142	0.1199	0.1296	0.1446	0.1403	0.1336	0.0111
102	0.0102	0.0107	0.0128	0.0021	0.0090	0.0047	0.0443	0.0595	0.0689	0.0736	0.0616	0.0129	0.1244	0.1395	0.1528	0.1561	0.1432	0.0144
108	0.012	0.0107	0.0159	0.0034	0.0105	0.0052	0.0515	0.0662	0.0776	0.0829	0.0696	0.0139	0.1425	0.1485	0.1716	0.1695	0.1580	0.0147
114	0.0017	0.0037	0.0108	-0.0011	0.0038	0.0051	0.057	0.0728	0.0791	0.0894	0.0746	0.0136	0.1451	0.1543	0.1725	0.1741	0.1615	0.0141
120	0.0088	0.013	0.0133	0.0106	0.0114	0.0021	0.0553	0.0643	0.0831	0.0846	0.0718	0.0144	0.1573	0.1651	0.1894	0.1928	0.1762	0.0176
126	0.0031	0.0127	0.0138	0.0008	0.0076	0.0066	0.0516	0.0607	0.073	0.0825	0.0670	0.0136	0.1446	0.1534	0.1779	0.1738	0.1624	0.0160
132	-0.0034	0.0107	0.0109	0.0018	0.0050	0.0070	0.0438	0.0611	0.07	0.0749	0.0625	0.0137	0.1586	0.1705	0.1927	0.1894	0.1778	0.0161
138	0.0108	0.0102	0.0138	0.0087	0.0109	0.0021	0.0441	0.0661	0.0743	0.0752	0.0649	0.0145	0.1554	0.1693	0.192	0.1937	0.1776	0.0185
144	0.0069	0.006	0.0064	-0.0049	0.0036	0.0057	0.0476	0.0602	0.0721	0.0777	0.0644	0.0134	0.1662	0.1751	0.1973	0.1998	0.1846	0.0165
150	0.0002	0.0067	0.0119	0.004	0.0057	0.0049	0.0393	0.0486	0.0658	0.0681	0.0555	0.0138	0.1703	0.1814	0.2027	0.2092	0.1909	0.0182
156	0.0032	0.01	0.0057	0.006	0.0062	0.0028	0.0479	0.0618	0.0776	0.0792	0.0666	0.0147	0.1715	0.1839	0.2067	0.2102	0.1931	0.0185
162	0.0011	0.011	0.0147	0.0035	0.0076	0.0064	0.0592	0.0654	0.086	0.0879	0.0746	0.0145	0.1638	0.1762	0.199	0.2046	0.1859	0.0192
168	0.0013	0.0178	0.0135	0.0017	0.0086	0.0084	0.0549	0.0666	0.0858	0.0872	0.0736	0.0156	0.1656	0.1784	0.2001	0.1973	0.1854	0.0163
174	0.005	0.0134	0.013	0.0079	0.0098	0.0041	0.0505	0.0678	0.0708	0.0785	0.0669	0.0118	0.162	0.1766	0.1993	0.1978	0.1839	0.0179
180	0.0015	0.0105	0.0147	0.0106	0.0093	0.0056	0.0612	0.0689	0.0819	0.0904	0.0756	0.0131	0.174	0.1899	0.2132	0.2095	0.1967	0.0182
186	0.0049	0.0065	0.0079	0.0031	0.0056	0.0021	0.0613	0.0693	0.0891	0.0914	0.0778	0.0148	0.1678	0.1842	0.2058	0.2099	0.1919	0.0196
192	0.0015	0.0101	0.0087	0.007	0.0068	0.0038	0.0611	0.0691	0.0865	0.0919	0.0772	0.0145	0.1703	0.183	0.2093	0.2156	0.1946	0.0215
198	-0.0046	0.0061	0.0074	-0.0032	0.0014	0.0062	0.0531	0.0622	0.0815	0.0871	0.0710	0.0160	0.1724	0.1881	0.2116	0.2155	0.1969	0.0203
204	0.0056	0.0087	0.0112	0.0081	0.0084	0.0023	0.0599	0.0685	0.0881	0.0934	0.0775	0.0159	0.167	0.1804	0.2017	0.2013	0.1876	0.0170
210	-0.0003	0.0139	0.0118	0.0024	0.0070	0.0070	0.0668	0.0751	0.1023	0.1073	0.0879	0.0199	0.1731	0.1889	0.2139	0.2175	0.1984	0.0211
216	0.0022	0.0141	0.0147	0.0027	0.0084	0.0069	0.0615	0.0712	0.0871	0.0932	0.0783	0.0145	0.1777	0.1929	0.2132	0.2129	0.1992	0.0172
222	0.0031	0.0106	0.0093	-0.0035	0.0049	0.0065	0.0659	0.0703	0.0862	0.096	0.0796	0.0140	0.171	0.1817	0.2063	0.2058	0.1912	0.0177
228	-0.0003	0.0085	0.0078	0.0042	0.0051	0.0040	0.0658	0.0761	0.0953	0.1035	0.0852	0.0173	0.1792	0.1892	0.2159	0.2193	0.2009	0.0198
234	0.0022	0.0153	0.0125	0.0079	0.0095	0.0057	0.0654	0.0753	0.0914	0.0993	0.0829	0.0153	0.1788	0.1871	0.2139	0.2156	0.1989	0.0187
240	0.0082	0.0056	0.0135	0.0051	0.0081	0.0038	0.0555	0.067	0.0926	0.0946	0.0774	0.0193	0.1787	0.1942	0.2167	0.2192	0.2022	0.0193
246	0.0063	0.014	0.0108	0.007	0.0095	0.0036	0.0665	0.0838	0.1026	0.1099	0.0907	0.0195	0.1785	0.1958	0.2213	0.2249	0.2051	0.0220
252	-0.0006	0.0115	0.0158	-0.0007	0.0065	0.0084	0.063	0.0787	0.0924	0.0974	0.0829	0.0154	0.1881	0.2044	0.2324	0.235	0.2150	0.0226
258	0.0038	0.0107	0.0098	0.0066	0.0077	0.0032	0.0798	0.0841	0.1094	0.1165	0.0975	0.0182	0.1819	0.1974	0.2209	0.2267	0.2067	0.0208
264	0.0061	0.0112	0.0144	0.0067	0.0096	0.0039	0.0647	0.0845	0.0946	0.1039	0.0869	0.0168	0.1915	0.2052	0.2325	0.2319	0.2153	0.0203
270	-0.0036	0.0106	0.0116	0.004	0.0057	0.0070	0.0628	0.0724	0.0961	0.1036	0.0837	0.0193	0.1936	0.2075	0.2336	0.2385	0.2183	0.0214
276	-0.0052	0.0077	0.0108	0.0037	0.0043	0.0069	0.0658	0.0821	0.102	0.1099	0.0900	0.0199	0.1885	0.203	0.2309	0.2366	0.2148	0.0228
282	-0.0012	0.0024	0.0075	0.0037	0.0031	0.0036	0.0787	0.0793	0.1027	0.1153	0.0940	0.0181	0.1934	0.2067	0.2324	0.2381	0.2177	0.0212
288	0.0119	0.0127	0.0148	-0.0003	0.0098	0.0068	0.0716	0.0798	0.1028	0.1108	0.0913	0.0186	0.1924	0.2047	0.2325	0.2299	0.2149	0.0195
294	0.0078	0.0101	0.0102	-0.0015	0.0067	0.0055	0.073	0.0788	0.1076	0.1147	0.0935	0.0207	0.2009	0.2159	0.2397	0.2413	0.2245	0.0195

FIG 3 Data. FRET histograms and FRET state occupancy data. Mean probabilities per histogram per and errors were determined as described in Materials and methods. Columns are colored as in Figure 3.

GPΔmuc (no Ca2+)

FRET histograms

bins	pH7	error	pH6	error	pH5.2	error	pH4.5	error
-0.1	0.0004	0.0001	0.0005	0.0001	0.0001	0.0001	0.0008	0.0003
-0.06	0.0003	0.0001	0.0008	0.0001	0.0016	0.0004	0.0008	0.0003
-0.02	0.0008	0.0001	0.0013	0.0001	0.0032	0.0007	0.0039	0.0012
0.02	0.0044	0.0007	0.0045	0.0001	0.0071	0.0015	0.008	0.0006
0.06	0.007	0.0002	0.0065	0.0006	0.0118	0.0023	0.0196	0.0025
0.1	0.0116	0.0012	0.0129	0.0008	0.0237	0.005	0.0375	0.0036
0.14	0.0186	0.0004	0.0179	0.0004	0.0337	0.005	0.0459	0.0043
0.18	0.0239	0.0016	0.0192	0.0011	0.0368	0.004	0.0526	0.0031
0.22	0.0253	0.0014	0.0231	0.0011	0.0365	0.0042	0.0599	0.0072
0.26	0.0277	0.0012	0.0255	0.0006	0.0382	0.004	0.0529	0.005
0.3	0.0291	0.002	0.0248	0.0011	0.0393	0.0047	0.0514	0.005
0.34	0.0289	0.0022	0.0257	0.0011	0.0318	0.0038	0.047	0.0052
0.38	0.026	0.0034	0.0252	0.001	0.0291	0.004	0.0393	0.004
0.42	0.0271	0.0024	0.0244	0.0004	0.0268	0.0054	0.0336	0.0057
0.46	0.0249	0.0018	0.0257	0.0015	0.0256	0.005	0.0298	0.0036
0.5	0.0246	0.0019	0.025	0.0015	0.0223	0.0053	0.0298	0.0041
0.54	0.0205	0.0025	0.0226	0.0014	0.0197	0.0043	0.0272	0.004
0.58	0.021	0.0019	0.0229	0.0008	0.0175	0.0046	0.024	0.0011
0.62	0.0215	0.0012	0.0228	0.0011	0.0206	0.0061	0.0214	0.0019
0.66	0.022	0.0016	0.0232	0.0013	0.0181	0.0039	0.0172	0.0027
0.7	0.0275	0.0016	0.0216	0.0017	0.0202	0.0045	0.0139	0.0009
0.74	0.0294	0.0026	0.0217	0.0006	0.0192	0.0032	0.0149	0.0014
0.78	0.0317	0.0011	0.0255	0.0014	0.0253	0.0038	0.0167	0.0005
0.82	0.0379	0.0031	0.0304	0.0006	0.0237	0.0039	0.0215	0.0041
0.86	0.048	0.0036	0.0385	0.0009	0.0286	0.0048	0.0225	0.0038
0.9	0.0652	0.0074	0.0551	0.003	0.043	0.0048	0.0294	0.0041
0.94	0.0863	0.007	0.0795	0.005	0.0597	0.0025	0.0482	0.0074
0.98	0.1254	0.005	0.1298	0.0056	0.0999	0.0032	0.069	0.008
1.02	0.1066	0.0089	0.1248	0.0035	0.1077	0.0037	0.0693	0.0149
1.06	0.0416	0.0025	0.0643	0.0015	0.0577	0.0023	0.0441	0.0035
1.1	0.0187	0.0015	0.0277	0.0008	0.0351	0.0017	0.0275	0.0005
1.14	0.0086	0.0009	0.014	0.0004	0.0178	0.0007	0.012	0.0015
1.18	0.0074	0.0012	0.0126	0.0004	0.0184	0.0022	0.0088	0.0016

FRET state occupancies

	High FRET	error	Int. FRET	error	Low FRET	error
**pH7**	0.6821	0.021	0.2047	0.0139	0.1132	0.0137
**pH6**	0.6078	0.0176	0.2227	0.0116	0.1695	0.0107
**pH5.2**	0.545	0.0297	0.1923	0.0181	0.2627	0.02
**pH4.5**	0.3912	0.0341	0.2575	0.0185	0.3513	0.0267

GPΔmuc + 0.5 mM Ca2+

FRET histograms

bins	pH7	error	pH6	error	pH5.2	error	pH4.5	error
-0.1	0.0007	0.0002	0.0005	0.0004	0.0001	0.0001	0.0004	0.0002
-0.06	0.0009	0.0001	0.0012	0.0002	0.0007	0.0003	0.001	0.0004
-0.02	0.002	0	0.003	0.0004	0.0036	0.0006	0.0028	0.0007
0.02	0.0077	0.001	0.0091	0.0008	0.0107	0.0019	0.0089	0.0019
0.06	0.0102	0.0011	0.017	0.0009	0.0205	0.0004	0.0165	0.0016
0.1	0.016	0.0013	0.028	0.0008	0.0366	0.0024	0.0368	0.0062
0.14	0.0247	0.0008	0.0386	0.0028	0.0555	0.0039	0.0465	0.0058
0.18	0.0275	0.0011	0.0411	0.0013	0.0631	0.0043	0.058	0.0041
0.22	0.0329	0.0016	0.0426	0.0035	0.0653	0.004	0.0639	0.001
0.26	0.031	0.003	0.0405	0.0031	0.0677	0.0039	0.057	0.0028
0.3	0.033	0.0023	0.0408	0.0043	0.0614	0.0024	0.0536	0.0039
0.34	0.0342	0.0021	0.0364	0.0039	0.0573	0.0041	0.0449	0.0048
0.38	0.0361	0.0041	0.0301	0.0035	0.0514	0.0037	0.0409	0.0068
0.42	0.0293	0.0032	0.0291	0.0022	0.0429	0.0037	0.035	0.0054
0.46	0.0281	0.0049	0.0296	0.003	0.035	0.0029	0.0312	0.0039
0.5	0.024	0.003	0.025	0.0006	0.0306	0.0049	0.0269	0.0024
0.54	0.0206	0.0034	0.025	0.0023	0.0274	0.0036	0.0232	0.0017
0.58	0.0227	0.0038	0.0204	0.001	0.0255	0.0027	0.0229	0.0022
0.62	0.0219	0.0038	0.0228	0.0013	0.0247	0.0038	0.0194	0.0025
0.66	0.0219	0.003	0.0193	0.0016	0.0256	0.0037	0.02	0.002
0.7	0.0235	0.0034	0.0228	0.0009	0.0247	0.0037	0.0192	0.0034
0.74	0.0252	0.0014	0.0236	0.0017	0.0218	0.003	0.0227	0.0031
0.78	0.0299	0.0009	0.0268	0.0007	0.0204	0.0033	0.0216	0.0016
0.82	0.0293	0.0039	0.0307	0.0019	0.0219	0.0022	0.0268	0.0043
0.86	0.0347	0.0036	0.0425	0.0043	0.027	0.0032	0.0337	0.0037
0.9	0.0445	0.0033	0.0536	0.0045	0.0357	0.0049	0.0447	0.0031
0.94	0.0719	0.0059	0.0732	0.0051	0.0404	0.0068	0.0646	0.0036
0.98	0.1105	0.0031	0.0952	0.0035	0.0376	0.0052	0.0679	0.0087
1.02	0.0958	0.0042	0.0747	0.0046	0.0286	0.0034	0.0432	0.003
1.06	0.0523	0.0047	0.0319	0.0036	0.0165	0.0015	0.0202	0.001
1.1	0.0253	0.003	0.0132	0.0013	0.0101	0.0009	0.0133	0.0026
1.14	0.0134	0.0018	0.0052	0.001	0.0056	0.0011	0.0064	0.0015
1.18	0.0184	0.0058	0.0065	0.0014	0.0041	0.0015	0.0059	0.0012

FRET state occupancies

	High FRET	error	Int. FRET	error	Low FRET	error
**pH7**	0.5531	0.0276	0.2361	0.0176	0.2109	0.0188
**pH6**	0.4814	0.0229	0.2292	0.0125	0.2894	0.0174
**pH5.2**	0.378	0.0237	0.2348	0.0147	0.3872	0.021
**pH4.5**	0.3101	0.0288	0.2509	0.018	0.439	0.0239

GP_CL (no Ca2+)

FRET histograms

bins	pH7	error	pH6	error	pH5.2	error	pH4.5	error
-0.1	0.0008	0.0004	0.0041	0.0011	0.0008	0.0005	0	0
-0.06	0.0002	0.0002	0.0011	0.0009	0.0005	0.0002	0.0003	0.0001
-0.02	0.0014	0.0002	0.0026	0.0016	0.0009	0.0002	0.0013	0.0003
0.02	0.0023	0.0008	0.008	0.002	0.0046	0.001	0.0064	0.0002
0.06	0.0063	0.0024	0.008	0.0011	0.0117	0.0019	0.0136	0.0011
0.1	0.0101	0.0016	0.0158	0.0025	0.023	0.0019	0.03	0.0031
0.14	0.0149	0.0017	0.021	0.0045	0.0401	0.0034	0.0412	0.0012
0.18	0.0171	0.0007	0.0245	0.0039	0.0393	0.0037	0.0529	0.0016
0.22	0.0185	0.0035	0.0306	0.0067	0.0418	0.0025	0.0555	0.0011
0.26	0.0168	0.0003	0.0286	0.0041	0.0422	0.005	0.057	0.002
0.3	0.0152	0.0007	0.0308	0.0033	0.0439	0.0044	0.0494	0.0014
0.34	0.0183	0.0002	0.0257	0.0036	0.0402	0.0058	0.0549	0.0006
0.38	0.0166	0.0021	0.0251	0.0057	0.037	0.0006	0.0501	0.0018
0.42	0.0197	0.0013	0.0251	0.0042	0.0347	0.003	0.0422	0.0039
0.46	0.0222	0.0025	0.0259	0.0035	0.0313	0.0029	0.0372	0.0008
0.5	0.0218	0.001	0.0268	0.0032	0.0336	0.0032	0.0366	0.0014
0.54	0.0227	0.0022	0.0179	0.0036	0.0288	0.0033	0.029	0.0026
0.58	0.0243	0.0027	0.0228	0.0024	0.0294	0.0035	0.0277	0.0013
0.62	0.0243	0.0016	0.021	0.0012	0.0265	0.0032	0.0238	0.0007
0.66	0.0263	0.002	0.0225	0.0026	0.0267	0.0009	0.0215	0.0018
0.7	0.0315	0.0042	0.0204	0.0033	0.0268	0.0037	0.0205	0.0035
0.74	0.0289	0.0046	0.0211	0.0023	0.029	0.0024	0.0224	0.0028
0.78	0.0353	0.0055	0.023	0.004	0.0273	0.0029	0.0211	0.0029
0.82	0.0429	0.0054	0.0325	0.0018	0.0331	0.0005	0.0184	0.0022
0.86	0.057	0.0035	0.0394	0.0027	0.0425	0.0051	0.0289	0.0029
0.9	0.0638	0.0069	0.0557	0.0035	0.0486	0.0059	0.0308	0.0029
0.94	0.0931	0.0064	0.0802	0.0041	0.0541	0.0068	0.0369	0.0058
0.98	0.1323	0.0155	0.1093	0.0037	0.0735	0.0127	0.0615	0.0086
1.02	0.1258	0.0167	0.1109	0.0119	0.0706	0.0105	0.0614	0.0087
1.06	0.056	0.0053	0.0645	0.006	0.0308	0.0048	0.0359	0.0017
1.1	0.0205	0.005	0.0266	0.0027	0.0153	0.0021	0.0184	0.002
1.14	0.0073	0.0021	0.0138	0.0024	0.0069	0.0009	0.0061	0.0005
1.18	0.0059	0.0018	0.0147	0.0033	0.0043	0.0001	0.0074	0.0021

FRET state occupancies

	High FRET	error	Int. FRET	error	Low FRET	error
**pH7**	0.6265	0.028	0.2181	0.0207	0.1554	0.0158
**pH6**	0.5572	0.0329	0.2356	0.0217	0.2071	0.0201
**pH5.2**	0.4458	0.0337	0.2815	0.0232	0.2727	0.0247
**pH4.5**	0.3159	0.0335	0.2759	0.0201	0.4082	0.0252

GP_CL + 0.5 mM Ca2+

FRET histograms

bins	pH7	error	pH6	error	pH5.2	error	pH4.5	error
-0.1	0.0022	0.0003	0.0016	0.0005	0.0018	0.0006	0.0053	0.0002
-0.06	0.0013	0.0001	0.001	0.0002	0.0016	0.0003	0.0044	0.0012
-0.02	0.0033	0.0003	0.0035	0.0003	0.004	0.0004	0.0091	0.0023
0.02	0.0119	0.0003	0.0096	0.0013	0.011	0.0016	0.0234	0.0039
0.06	0.0174	0.0006	0.0192	0.0009	0.0267	0.003	0.0433	0.0043
0.1	0.035	0.0043	0.0346	0.0031	0.0356	0.003	0.0598	0.0036
0.14	0.0435	0.0054	0.0436	0.0036	0.0555	0.0074	0.0836	0.0097
0.18	0.0538	0.0076	0.0508	0.0029	0.0597	0.0052	0.0946	0.009
0.22	0.0521	0.0043	0.0477	0.0044	0.0619	0.0063	0.0815	0.0052
0.26	0.0527	0.0046	0.0457	0.0052	0.0595	0.007	0.0814	0.0074
0.3	0.0526	0.0032	0.042	0.0046	0.0582	0.0078	0.0626	0.0073
0.34	0.0533	0.0041	0.0431	0.0025	0.0512	0.0095	0.0524	0.0029
0.38	0.0421	0.0018	0.0398	0.0025	0.0425	0.0065	0.0401	0.0013
0.42	0.039	0.004	0.0404	0.0049	0.0354	0.0053	0.0384	0.0019
0.46	0.0349	0.0021	0.032	0.0017	0.0317	0.0034	0.0349	0.0011
0.5	0.0346	0.0014	0.028	0.0014	0.0329	0.0043	0.0278	0.0019
0.54	0.0324	0.0034	0.0267	0.002	0.0247	0.002	0.0256	0.0023
0.58	0.0279	0.003	0.0252	0.0014	0.0289	0.0037	0.0243	0.0019
0.62	0.0292	0.0014	0.025	0.0022	0.0225	0.0018	0.0198	0.0037
0.66	0.0308	0.0041	0.0225	0.001	0.0245	0.0043	0.0188	0.0027
0.7	0.0291	0.0038	0.0221	0.0016	0.0214	0.0017	0.0147	0.0008
0.74	0.023	0.0016	0.0234	0.002	0.0239	0.0019	0.0212	0.0024
0.78	0.0246	0.0017	0.0265	0.0026	0.0235	0.0014	0.0171	0.004
0.82	0.0254	0.0031	0.0323	0.0013	0.0238	0.0024	0.0146	0.0029
0.86	0.0275	0.0027	0.033	0.0018	0.028	0.0025	0.0149	0.0038
0.9	0.0337	0.0008	0.0492	0.0011	0.0308	0.0041	0.018	0.0045
0.94	0.0463	0.0027	0.0541	0.001	0.0412	0.0065	0.0217	0.0057
0.98	0.0607	0.0056	0.073	0.0083	0.0539	0.0089	0.019	0.0076
1.02	0.0473	0.005	0.0528	0.0045	0.0458	0.0061	0.011	0.0037
1.06	0.018	0.0025	0.0305	0.0037	0.0206	0.0019	0.0072	0.0026
1.1	0.0085	0.0011	0.0126	0.0029	0.0082	0.0023	0.004	0.0011
1.14	0.0025	0.0005	0.0035	0.0014	0.005	0.0018	0.0025	0.0007
1.18	0.0031	0.0016	0.005	0.0019	0.004	0.002	0.003	0.0012

FRET state occupancies

	High FRET	error	Int. FRET	error	Low FRET	error
**pH7**	0.4001	0.024	0.2694	0.0161	0.3305	0.0245
**pH6**	0.3445	0.0287	0.2808	0.0171	0.3747	0.0232
**pH5.2**	0.307	0.0254	0.2802	0.015	0.4128	0.023
**pH4.5**	0.2039	0.0282	0.287	0.0165	0.5091	0.0238

GP_CL + sNPC1-C (no Ca2+)

FRET histograms

bins	pH7	error	pH6	error	pH5.2	error	pH4.5	error
-0.1	0.0009	0.0002	0.001	0.0005	0.0019	0.0005	0.0002	0.0001
-0.06	0.0005	0.0002	0.001	0.0003	0.0014	0.0001	0.0005	0.0002
-0.02	0.0038	0.0001	0.0013	0.0004	0.0028	0.0001	0.0029	0.0008
0.02	0.0076	0.0012	0.0056	0.0007	0.0095	0.0015	0.0075	0.0012
0.06	0.0115	0.0037	0.0099	0.001	0.013	0.001	0.0147	0.0028
0.1	0.022	0.0027	0.0183	0.0005	0.0253	0.0025	0.0275	0.0031
0.14	0.0288	0.0042	0.0231	0.0036	0.0335	0.0063	0.0409	0.0049
0.18	0.0349	0.004	0.0324	0.0037	0.0486	0.0056	0.0499	0.0057
0.22	0.0429	0.0055	0.0335	0.0043	0.056	0.007	0.0455	0.0015
0.26	0.0459	0.0077	0.0381	0.0044	0.0456	0.0077	0.0532	0.0053
0.3	0.0385	0.0059	0.0385	0.0039	0.0572	0.0041	0.0512	0.0034
0.34	0.0453	0.0094	0.0425	0.0029	0.0527	0.0052	0.0524	0.0024
0.38	0.042	0.0077	0.0405	0.004	0.0474	0.0039	0.0475	0.001
0.42	0.0461	0.0079	0.0418	0.005	0.04	0.0055	0.0399	0.0019
0.46	0.0386	0.0075	0.0434	0.0052	0.0381	0.0067	0.0381	0.0024
0.5	0.038	0.0064	0.0415	0.0039	0.0398	0.0055	0.0385	0.0039
0.54	0.0376	0.0069	0.0438	0.0079	0.0318	0.0066	0.0368	0.0057
0.58	0.0349	0.0083	0.0407	0.0069	0.0377	0.0073	0.0304	0.0037
0.62	0.0299	0.0068	0.0388	0.0059	0.0281	0.0064	0.0277	0.0054
0.66	0.0288	0.0058	0.0372	0.0055	0.0281	0.0023	0.0245	0.003
0.7	0.0271	0.0049	0.0322	0.0048	0.0353	0.005	0.0244	0.0033
0.74	0.0271	0.0059	0.0327	0.0032	0.0277	0.0045	0.0231	0.0038
0.78	0.0317	0.0062	0.0322	0.0053	0.0325	0.0033	0.0209	0.0031
0.82	0.0344	0.0051	0.0314	0.0038	0.0342	0.0011	0.0229	0.0029
0.86	0.0312	0.0032	0.0378	0.0084	0.0391	0.0036	0.0267	0.0024
0.9	0.0449	0.0061	0.047	0.0093	0.0467	0.002	0.0378	0.0021
0.94	0.0505	0.006	0.0573	0.0117	0.0374	0.0022	0.0581	0.0029
0.98	0.0679	0.0047	0.0657	0.0085	0.0444	0.0018	0.0773	0.0119
1.02	0.0518	0.0067	0.045	0.0061	0.0309	0.0039	0.0465	0.0073
1.06	0.0273	0.0061	0.0207	0.001	0.0181	0.0025	0.0175	0.0027
1.1	0.0133	0.0032	0.0119	0.0009	0.01	0.0017	0.0064	0.0022
1.14	0.0065	0.0024	0.0062	0.0012	0.0026	0.0011	0.0043	0.0027
1.18	0.008	0.002	0.0068	0.0021	0.0025	0.0004	0.0043	0.0025

FRET state occupancies

	High FRET	error	Int. FRET	error	Low FRET	error
**pH7**	0.3472	0.02	0.2973	0.0167	0.3555	0.0188
**pH6**	0.302	0.0264	0.2986	0.0202	0.3994	0.0236
**pH5.2**	0.283	0.0277	0.2819	0.0226	0.4351	0.0273
**pH4.5**	0.2234	0.0223	0.2418	0.0227	0.5348	0.0272

GP_CL + sNPC1-C + 0.5 mM Ca2+

FRET histograms

bins	pH7	error	pH6	error	pH5.2	error	pH4.5	error
-0.1	0.0015	0.0002	0.0013	0.0003	0.002	0.0009	0.0021	0.0002
-0.06	0.0023	0.0004	0.0014	0.0002	0.002	0.0003	0.0034	0.0009
-0.02	0.0038	0.0005	0.0037	0.0006	0.0045	0.001	0.0049	0.0006
0.02	0.01	0.0008	0.0093	0.0016	0.0138	0.0008	0.0159	0.0014
0.06	0.0217	0.0037	0.019	0.0013	0.023	0.0017	0.0296	0.0006
0.1	0.0306	0.0047	0.031	0.0002	0.0403	0.001	0.0495	0.003
0.14	0.0428	0.0064	0.0463	0.003	0.0588	0.0019	0.0632	0.0069
0.18	0.0529	0.0089	0.0543	0.0025	0.0638	0.0049	0.0705	0.0044
0.22	0.0561	0.0068	0.0548	0.0011	0.0651	0.0013	0.0736	0.0024
0.26	0.0547	0.009	0.0599	0.0003	0.0741	0.003	0.0775	0.0068
0.3	0.0498	0.0063	0.0593	0.0026	0.066	0.0014	0.0719	0.0029
0.34	0.0512	0.0065	0.054	0.0005	0.0509	0.0036	0.0659	0.004
0.38	0.044	0.0059	0.0507	0.001	0.0468	0.0011	0.0589	0.0056
0.42	0.0418	0.0031	0.0469	0.0013	0.0318	0.0015	0.0481	0.0019
0.46	0.0369	0.0011	0.0387	0.0038	0.034	0.0015	0.0429	0.0021
0.5	0.0329	0.0009	0.0398	0.0015	0.0274	0.0004	0.0329	0.0035
0.54	0.0296	0.0027	0.0354	0.0026	0.0203	0.0017	0.0331	0.0024
0.58	0.0262	0.0021	0.0316	0.0034	0.0244	0.0024	0.0267	0.0026
0.62	0.0272	0.0039	0.0307	0.0046	0.0208	0.0023	0.0231	0.0006
0.66	0.0232	0.0023	0.031	0.0052	0.0192	0.0011	0.0221	0.0015
0.7	0.0252	0.0027	0.0299	0.0045	0.0202	0.0011	0.0214	0.002
0.74	0.0264	0.0035	0.03	0.0046	0.0239	0.0008	0.0184	0.0007
0.78	0.0256	0.0046	0.0245	0.0035	0.0304	0.001	0.0169	0.0009
0.82	0.0263	0.004	0.0291	0.0048	0.0354	0.0034	0.0175	0.0018
0.86	0.0304	0.0029	0.0265	0.0036	0.0376	0.0028	0.0157	0.0012
0.9	0.0375	0.0038	0.0285	0.0037	0.0403	0.0035	0.0173	0.0013
0.94	0.0465	0.0052	0.0373	0.0067	0.0455	0.0062	0.0208	0.0012
0.98	0.0617	0.0064	0.0498	0.0087	0.0409	0.0024	0.0284	0.0063
1.02	0.0466	0.0039	0.0298	0.0051	0.0253	0.0068	0.0177	0.0023
1.06	0.0207	0.0014	0.0105	0.0026	0.0073	0.0013	0.0071	0.0009
1.1	0.0072	0.0004	0.0037	0.0008	0.002	0.0004	0.0018	0.0004
1.14	0.0041	0.0008	0.001	0.0006	0.0013	0.0003	0.0007	0.0004
1.18	0.0026	0.0009	0.0003	0.0001	0.0008	0.0002	0.0008	0.0004

FRET state occupancies

	High FRET	error	Int. FRET	error	Low FRET	error
**pH7**	0.3073	0.0214	0.2933	0.018	0.3994	0.0214
**pH6**	0.2857	0.0218	0.2988	0.0158	0.4155	0.0197
**pH5.2**	0.2529	0.0284	0.2665	0.0206	0.4806	0.0289
**pH4.5**	0.1968	0.0212	0.2657	0.0206	0.5375	0.0259

FIG 4 Data. FRET histograms and FRET state occupancy data. Columns are colored as in Figure 4.

GPΔmuc (no Ca2+)

FRET histograms

bins	pH7	error	pH4.5	error	-->pH7	error
-0.1	Repeat from Fig 3.		Repeat from Fig 3.		0.0005	0.0003
-0.06					0.0005	0.0003
-0.02					0.0011	0.0006
0.02					0.0017	0.0004
0.06					0.0049	0.0013
0.1					0.0088	0.0024
0.14					0.0126	0.0038
0.18					0.0143	0.0042
0.22					0.0155	0.0047
0.26					0.015	0.0053
0.3					0.0201	0.0036
0.34					0.015	0.0044
0.38					0.0155	0.003
0.42					0.013	0.0029
0.46					0.0149	0.0027
0.5					0.0156	0.0033
0.54					0.0162	0.0034
0.58					0.0169	0.0038
0.62					0.0178	0.0033
0.66					0.0169	0.0036
0.7					0.0227	0.0038
0.74					0.0286	0.0052
0.78					0.0297	0.0021
0.82					0.0375	0.0035
0.86					0.0528	0.0028
0.9					0.0698	0.0055
0.94					0.1122	0.003
0.98					0.1611	0.0156
1.02					0.1354	0.015
1.06					0.0657	0.0061
1.1					0.0257	0.004
1.14					0.0128	0.0017
1.18					0.0095	0.0014

FRET state occupancies

	High FRET	error	Low FRET	error
**pH7**	0.6821	0.021	0.1132	0.0137
**pH4.5**	0.3912	0.0341	0.3513	0.0267
**-->pH7**	0.7635	0.0242	0.0977	0.0151

GPΔmuc + 0.5 mM Ca2+

FRET histograms

bins	pH7	error	pH4.5	error	-->pH7	error
-0.1	Repeat from Fig 3.		Repeat from Fig 3.		0.0012	0.0005
-0.06					0.0011	0.0003
-0.02					0.0017	0.0002
0.02					0.0032	0.0007
0.06					0.0067	0.0017
0.1					0.0115	0.0018
0.14					0.0154	0.0024
0.18					0.0216	0.0012
0.22					0.0232	0.0013
0.26					0.0258	0.002
0.3					0.0287	0.0021
0.34					0.0265	0.0033
0.38					0.0278	0.0017
0.42					0.0255	0.002
0.46					0.0263	0.0019
0.5					0.0232	0.002
0.54					0.0249	0.0012
0.58					0.0223	0.0036
0.62					0.0226	0.0023
0.66					0.0279	0.0035
0.7					0.0296	0.0051
0.74					0.0287	0.0025
0.78					0.0368	0.0035
0.82					0.0445	0.0039
0.86					0.0502	0.0045
0.9					0.0661	0.0019
0.94					0.0825	0.005
0.98					0.1265	0.0009
1.02					0.1031	0.0047
1.06					0.0369	0.006
1.1					0.0143	0.0015
1.14					0.0076	0.0006
1.18					0.0061	0.0008

FRET state occupancies

	High FRET	error	Low FRET	error
**pH7**	0.5531	0.0276	0.2109	0.0188
**pH4.5**	0.3101	0.0288	0.439	0.0239
**-->pH7**	0.6534	0.0233	0.1552	0.0156

GP_CL (no Ca2+)

FRET histograms

bins	pH7	error	pH4.5	error	-->pH7	error
-0.1	Repeat from Fig 3.		Repeat from Fig 3.		0.0047	0.0017
-0.06					0.0025	0.0002
-0.02					0.0049	0.0005
0.02					0.0135	0.0021
0.06					0.0194	0.0015
0.1					0.0356	0.0013
0.14					0.049	0.0044
0.18					0.0547	0.0039
0.22					0.0574	0.006
0.26					0.0554	0.0058
0.3					0.0521	0.0039
0.34					0.0486	0.0043
0.38					0.0398	0.0065
0.42					0.0386	0.0038
0.46					0.0303	0.0025
0.5					0.0309	0.0047
0.54					0.0272	0.0036
0.58					0.0251	0.0028
0.62					0.0277	0.0044
0.66					0.0261	0.0006
0.7					0.0241	0.0025
0.74					0.0232	0.0006
0.78					0.0262	0.0022
0.82					0.0266	0.0013
0.86					0.0322	0.0025
0.9					0.0365	0.006
0.94					0.0408	0.0076
0.98					0.0563	0.0128
1.02					0.0489	0.007
1.06					0.0228	0.0039
1.1					0.0098	0.0015
1.14					0.0043	0.0006
1.18					0.0049	0.0013

FRET state occupancies

	High FRET	error	Low FRET	error
pH7	0.6265	0.028	0.2109	0.0188
pH4.5	0.2059	0.0335	0.4082	0.0239
-->pH7	0.3721	0.0333	0.3281	0.0252

GP_CL + 0.5 mM Ca2+

FRET histograms

bins	pH7	error	pH4.5	error	-->pH7	error
-0.1	Repeat from Fig 3.		Repeat from Fig 3.		0.0061	0.0015
-0.06					0.0023	0.0003
-0.02					0.006	0.0014
0.02					0.0126	0.0012
0.06					0.0233	0.0009
0.1					0.0372	0.0029
0.14					0.0496	0.0029
0.18					0.0556	0.0045
0.22					0.0535	0.0008
0.26					0.0515	0.0026
0.3					0.0489	0.0004
0.34					0.048	0.0033
0.38					0.0407	0.0008
0.42					0.0333	0.0028
0.46					0.0373	0.0022
0.5					0.0324	0.0027
0.54					0.0309	0.0029
0.58					0.0302	0.0033
0.62					0.03	0.0013
0.66					0.0266	0.0048
0.7					0.0266	0.003
0.74					0.0252	0.0036
0.78					0.0233	0.0025
0.82					0.0323	0.0047
0.86					0.0286	0.004
0.9					0.0363	0.0021
0.94					0.0387	0.0056
0.98					0.055	0.0104
1.02					0.0421	0.0093
1.06					0.016	0.0039
1.1					0.0079	0.0009
1.14					0.0051	0.0006
1.18					0.0068	0.0029

FRET state occupancies

	High FRET	error	Low FRET	error
**pH7**	0.4001	0.024	0.3305	0.0245
**pH4.5**	0.2031	0.0282	0.5091	0.0238
**-->pH7**	0.3235	0.0217	0.3826	0.0237

GP_CL + sNPC1-C (no Ca2+)

FRET histograms

bins	pH7	error	pH4.5	error	-->pH7	error
-0.1	Repeat from Fig 3.		Repeat from Fig 3.		0.0026	0.0004
-0.06					0.0034	0.0001
-0.02					0.0063	0.0007
0.02					0.019	0.0014
0.06					0.0274	0.0028
0.1					0.0474	0.005
0.14					0.0683	0.005
0.18					0.0765	0.0084
0.22					0.0793	0.0116
0.26					0.0744	0.0068
0.3					0.0625	0.0045
0.34					0.0644	0.0017
0.38					0.0561	0.0006
0.42					0.0503	0.002
0.46					0.0378	0.0021
0.5					0.034	0.0007
0.54					0.031	0.0017
0.58					0.0247	0.0015
0.62					0.0231	0.0014
0.66					0.021	0.0028
0.7					0.0168	0.0019
0.74					0.0171	0.0034
0.78					0.0172	0.0024
0.82					0.0149	0.0012
0.86					0.0176	0.0025
0.9					0.0214	0.004
0.94					0.0248	0.0012
0.98					0.032	0.0015
1.02					0.0228	0.0035
1.06					0.0041	0.0008
1.1					0.0008	0.0001
1.14					0.0005	0.0001
1.18					0.0003	0

FRET state occupancies

	High FRET	error	Low FRET	error
**pH7**	0.3472	0.024	0.3555	0.0188
**pH4.5**	0.2234	0.0282	0.5348	0.0272
**-->pH7**	0.2657	0.0217	0.4235	0.0273

GP_CL + sNPC1-C + 0.5 mM Ca2+

FRET histograms

bins	pH7	error	pH4.5	error	-->pH7	error
-0.1	Repeat from Fig 3.		Repeat from Fig 3.		0.0028	0.0007
-0.06					0.0047	0.0004
-0.02					0.0087	0.0005
0.02					0.0214	0.0008
0.06					0.0367	0.0015
0.1					0.0535	0.0016
0.14					0.0762	0.0055
0.18					0.0863	0.0027
0.22					0.0793	0.0039
0.26					0.0766	0.0033
0.3					0.0669	0.0045
0.34					0.0595	0.0022
0.38					0.0528	0.0018
0.42					0.0474	0.0011
0.46					0.0375	0.0016
0.5					0.0306	0.0024
0.54					0.0282	0.0017
0.58					0.024	0.0018
0.62					0.0191	0.0009
0.66					0.0184	0.0016
0.7					0.0206	0.0015
0.74					0.0171	0.0013
0.78					0.0181	0.001
0.82					0.0192	0.0008
0.86					0.0176	0.0033
0.9					0.0165	0.0014
0.94					0.0192	0.0023
0.98					0.0175	0.0022
1.02					0.0108	0.001
1.06					0.0064	0.0022
1.1					0.0032	0.0012
1.14					0.0023	0.0009
1.18					0.0009	0.0003

FRET state occupancies

	High FRET	error	Low FRET	error
**pH7**	0.3073	0.0214	0.3575	0.0168
**pH4.5**	0.1968	0.0212	0.5388	0.0291
**-->pH7**	0.1758	0.0184	0.5528	0.0218

S1 FIG Data. Infectivity and fusion data. The data displayed in S1 Fig are presented as the average of three measurements (rep1-3) with error bars reflecting the standard deviation.

A. Relative Infectivity of GP* and GP (DiD) are expressed as a fraction of wild-type GP

	**GP***	**GP (DiD)**
rep1	0.6510	0.7016
rep2	0.6948	0.7097
rep3	0.5106	0.8648
**mean**	**0.6188**	**0.7587**
**stdev**	**0.0962**	**0.0920**

B. Relative Fusion expressed as a fraction of wild-type GP without Ca2+

	**GP + Ca2+**	**GP***	**GP* + Ca2+**	**GP*-Cy3/Cy5**	**GP*-Cy3/Cy5 + Ca2+**	**GP + KZ52**	**GP* + KZ52**	**GP*-Cy3/Cy5 + KZ52**
rep1	1.4303	0.8856	1.2125	0.6511	0.8939	0.1231	0.0870	0.0429
rep2	1.4207	0.8375	1.3826	0.7874	0.7530	0.2118	0.0319	0.0707
rep3	1.5277	0.9851	1.3265	0.7021	0.8823	0.1779	0.1284	0.1105
**relative mean**	**1.4596**	**0.9027**	**1.3072**	**0.7135**	**0.8431**	**0.1709**	**0.0824**	**0.0747**
**relative stdev**	**0.0592**	**0.0753**	**0.0867**	**0.0689**	**0.0782**	**0.0448**	**0.0484**	**0.0340**

S2 FIG DATA. Fluorescence dequenching data plotted in Fig 1. Three replicates for each experiment are provided (rep1-3), along with the mean and standard deviation. Columns are colored according to the plots in Fig 1.

A. GPDmuc + sNPC1-C

pH7

	**0.5 mM Ca2+**					**No Ca2+**				
**time**	**rep1**	**rep2**	**rep3**	**mean**	**error bars**	**rep1**	**rep2**	**rep3**	**mean**	**error bars**
0	0.0000	0.0000	0.0000	**0.0000**	**0.0000**	0.0000	0.0000	0.0000	0.0000	0.0000
6	0.0026	0.0043	0.0042	**0.0045**	**0.0011**	-0.0011	-0.0019	-0.0021	-0.0021	-0.0011
12	0.0021	0.0031	0.0020	**0.0027**	**0.0007**	0.0021	0.0013	0.0008	0.0011	0.0006
18	0.0033	0.0039	0.0037	**0.0035**	**0.0009**	0.0067	0.0068	0.0050	0.0047	0.0024
24	0.0015	0.0031	0.0035	**0.0039**	**0.0010**	0.0008	0.0003	0.0003	0.0004	0.0002
30	0.0040	0.0030	0.0045	**0.0046**	**0.0011**	0.0037	0.0010	0.0023	0.0029	0.0015
36	0.0068	0.0094	0.0073	**0.0071**	**0.0018**	0.0033	0.0077	0.0062	0.0046	0.0023
42	0.0058	0.0048	0.0027	**0.0049**	**0.0012**	0.0058	0.0065	0.0064	0.0044	0.0022
48	0.0031	0.0039	0.0043	**0.0049**	**0.0012**	0.0052	0.0015	0.0008	0.0051	0.0025
54	0.0087	0.0060	0.0064	**0.0076**	**0.0019**	0.0041	0.0073	0.0056	0.0040	0.0020
60	0.0066	0.0054	0.0043	**0.0062**	**0.0016**	0.0074	0.0092	0.0037	0.0048	0.0024
66	0.0071	0.0068	0.0096	**0.0082**	**0.0021**	0.0029	0.0066	0.0027	0.0043	0.0021
72	0.0089	0.0096	0.0075	**0.0085**	**0.0021**	0.0113	0.0078	0.0037	0.0054	0.0027
78	0.0129	0.0086	0.0065	**0.0084**	**0.0021**	0.0038	0.0060	0.0064	0.0047	0.0024
84	0.0067	0.0102	0.0089	**0.0100**	**0.0025**	0.0043	0.0032	0.0013	0.0027	0.0014
90	0.0071	0.0095	0.0064	**0.0108**	**0.0027**	0.0071	0.0016	0.0087	0.0067	0.0034
96	0.0106	0.0135	0.0108	**0.0091**	**0.0023**	0.0045	0.0038	0.0019	0.0035	0.0018
102	0.0081	0.0119	0.0095	**0.0088**	**0.0022**	0.0088	0.0081	0.0035	0.0054	0.0027
108	0.0047	0.0101	0.0107	**0.0105**	**0.0026**	0.0022	0.0099	0.0086	0.0066	0.0033
114	0.0141	0.0118	0.0159	**0.0123**	**0.0031**	0.0038	0.0042	0.0050	0.0037	0.0019
120	0.0072	0.0174	0.0145	**0.0108**	**0.0027**	0.0034	0.0077	0.0051	0.0043	0.0022
126	0.0090	0.0102	0.0075	**0.0096**	**0.0024**	0.0020	0.0025	0.0057	0.0033	0.0016
132	0.0086	0.0114	0.0139	**0.0112**	**0.0028**	0.0015	0.0010	0.0035	0.0022	0.0011
138	0.0121	0.0151	0.0090	**0.0098**	**0.0025**	0.0053	0.0097	0.0040	0.0046	0.0023
144	0.0075	0.0025	0.0073	**0.0079**	**0.0020**	0.0046	0.0035	0.0043	0.0035	0.0018
150	0.0092	0.0089	0.0074	**0.0089**	**0.0022**	0.0010	0.0052	0.0064	0.0047	0.0023
156	0.0055	0.0073	0.0126	**0.0081**	**0.0020**	0.0027	0.0022	0.0027	0.0027	0.0013
162	0.0084	0.0109	0.0078	**0.0109**	**0.0027**	-0.0001	0.0036	0.0026	0.0022	0.0011
168	0.0107	0.0099	0.0086	**0.0083**	**0.0021**	0.0078	0.0081	0.0070	0.0061	0.0031
174	0.0111	0.0154	0.0129	**0.0113**	**0.0028**	0.0066	0.0017	0.0037	0.0038	0.0019
180	0.0100	0.0104	0.0139	**0.0100**	**0.0025**	0.0081	0.0052	0.0069	0.0053	0.0026
186	0.0109	0.0104	0.0111	**0.0097**	**0.0024**	0.0014	0.0089	0.0117	0.0076	0.0038
192	0.0116	0.0090	0.0130	**0.0119**	**0.0030**	-0.0004	0.0030	0.0013	0.0034	0.0017
198	0.0049	0.0068	0.0104	**0.0082**	**0.0021**	0.0020	0.0055	0.0065	0.0042	0.0021
204	0.0089	0.0115	0.0102	**0.0108**	**0.0027**	0.0053	0.0044	0.0113	0.0060	0.0030
210	0.0145	0.0150	0.0118	**0.0135**	**0.0034**	0.0047	0.0059	0.0041	0.0049	0.0025
216	0.0059	0.0158	0.0109	**0.0126**	**0.0032**	0.0051	0.0016	0.0041	0.0041	0.0020
222	0.0108	0.0125	0.0073	**0.0101**	**0.0025**	0.0038	0.0062	0.0092	0.0069	0.0034
228	0.0158	0.0143	0.0124	**0.0121**	**0.0030**	0.0108	0.0042	0.0069	0.0055	0.0027
234	0.0108	0.0154	0.0132	**0.0118**	**0.0030**	0.0045	0.0061	0.0141	0.0059	0.0029
240	0.0098	0.0145	0.0099	**0.0087**	**0.0022**	0.0027	0.0017	0.0037	0.0024	0.0012
246	0.0161	0.0123	0.0180	**0.0124**	**0.0031**	0.0083	0.0014	0.0038	0.0056	0.0028
252	0.0089	0.0069	0.0091	**0.0092**	**0.0023**	0.0030	0.0034	0.0040	0.0038	0.0019
258	0.0063	0.0146	0.0078	**0.0111**	**0.0028**	0.0050	0.0085	0.0086	0.0070	0.0035
264	0.0120	0.0138	0.0126	**0.0112**	**0.0028**	0.0012	0.0025	0.0009	0.0021	0.0011
270	0.0115	0.0083	0.0104	**0.0105**	**0.0026**	0.0057	0.0036	0.0055	0.0040	0.0020
276	0.0137	0.0090	0.0077	**0.0111**	**0.0028**	0.0017	0.0047	0.0029	0.0034	0.0017
282	0.0076	0.0111	0.0085	**0.0092**	**0.0023**	0.0033	0.0029	0.0045	0.0034	0.0017
288	0.0120	0.0064	0.0091	**0.0113**	**0.0028**	0.0097	0.0074	0.0052	0.0051	0.0026
294	0.0090	0.0075	0.0142	**0.0110**	**0.0027**	0.0036	0.0059	0.0029	0.0057	0.0028

pH6

	0.5 mM Ca^{2+}					No Ca^{2+}				
**time**	**rep1**	**rep2**	**rep3**	**mean**	**error bars**	**rep1**	**rep2**	**rep3**	**mean**	**error bars**
0	0.0000	0.0000	0.0000	**0.0000**	**0.0000**	0.0000	0.0000	0.0000	0.0000	0.0000
6	0.0018	0.0008	0.0014	**0.0011**	**0.0006**	-0.0014	-0.0033	-0.0018	-0.0019	-0.0010
12	0.0045	0.0012	0.0029	**0.0039**	**0.0019**	-0.0006	-0.0025	-0.0032	-0.0024	-0.0012
18	0.0056	0.0016	0.0034	**0.0037**	**0.0019**	-0.0040	-0.0069	-0.0062	-0.0053	-0.0026
24	0.0024	0.0024	0.0025	**0.0046**	**0.0023**	-0.0032	-0.0022	-0.0025	-0.0027	-0.0013
30	0.0072	0.0051	0.0060	**0.0051**	**0.0025**	-0.0023	-0.0023	-0.0007	-0.0025	-0.0012
36	0.0022	0.0029	0.0044	**0.0031**	**0.0015**	-0.0044	-0.0018	-0.0048	-0.0045	-0.0022
42	0.0023	0.0040	0.0096	**0.0064**	**0.0032**	0.0002	0.0006	0.0005	0.0004	0.0002
48	0.0121	0.0037	0.0080	**0.0065**	**0.0033**	0.0003	0.0010	0.0007	0.0005	0.0002
54	0.0023	0.0081	0.0065	**0.0061**	**0.0030**	0.0017	0.0000	0.0004	0.0012	0.0006
60	0.0031	0.0079	0.0103	**0.0063**	**0.0031**	0.0016	0.0021	0.0009	0.0025	0.0012
66	0.0064	0.0038	0.0031	**0.0069**	**0.0035**	0.0045	0.0025	0.0031	0.0034	0.0017
72	0.0022	0.0070	0.0068	**0.0058**	**0.0029**	0.0024	0.0032	0.0056	0.0033	0.0017
78	0.0031	0.0071	0.0024	**0.0051**	**0.0026**	0.0003	0.0018	0.0026	0.0035	0.0017
84	0.0122	-0.0004	0.0061	**0.0070**	**0.0035**	0.0018	0.0007	0.0011	0.0014	0.0007
90	0.0097	0.0071	0.0072	**0.0071**	**0.0035**	0.0057	0.0016	0.0064	0.0042	0.0021
96	0.0031	0.0082	0.0032	**0.0041**	**0.0021**	0.0040	0.0044	0.0034	0.0034	0.0017
102	0.0096	0.0114	0.0059	**0.0071**	**0.0035**	0.0024	0.0010	0.0051	0.0048	0.0024
108	0.0127	0.0090	0.0019	**0.0073**	**0.0037**	0.0067	0.0067	0.0047	0.0049	0.0025
114	0.0054	0.0065	0.0093	**0.0070**	**0.0035**	0.0047	0.0028	0.0020	0.0026	0.0013
120	0.0095	0.0095	0.0019	**0.0070**	**0.0035**	0.0049	0.0032	0.0062	0.0046	0.0023
126	0.0019	0.0136	0.0115	**0.0081**	**0.0041**	0.0070	0.0060	0.0044	0.0039	0.0020
132	0.0130	0.0191	0.0207	**0.0108**	**0.0054**	0.0015	0.0031	0.0055	0.0046	0.0023
138	0.0041	0.0068	0.0081	**0.0067**	**0.0033**	0.0069	0.0122	0.0096	0.0072	0.0036
144	0.0076	0.0079	0.0079	**0.0072**	**0.0036**	0.0017	0.0078	0.0079	0.0059	0.0030
150	0.0178	-0.0043	0.0115	**0.0115**	**0.0058**	0.0050	0.0028	0.0030	0.0027	0.0013
156	0.0088	0.0077	0.0108	**0.0101**	**0.0050**	0.0010	0.0036	0.0012	0.0047	0.0023
162	0.0140	0.0095	0.0112	**0.0111**	**0.0055**	0.0047	0.0031	0.0018	0.0050	0.0025
168	0.0009	0.0024	0.0043	**0.0053**	**0.0027**	0.0045	0.0026	0.0074	0.0045	0.0023
174	0.0073	0.0037	0.0033	**0.0080**	**0.0040**	0.0075	0.0038	0.0040	0.0035	0.0018
180	0.0129	0.0105	0.0166	**0.0098**	**0.0049**	0.0000	0.0039	0.0015	0.0022	0.0011
186	0.0081	0.0026	-0.0007	**0.0063**	**0.0032**	0.0048	0.0036	0.0047	0.0040	0.0020
192	0.0093	0.0077	0.0111	**0.0087**	**0.0044**	0.0028	0.0031	0.0014	0.0029	0.0015
198	0.0172	0.0106	0.0127	**0.0118**	**0.0059**	0.0060	0.0015	0.0054	0.0057	0.0028
204	0.0060	0.0150	0.0096	**0.0094**	**0.0047**	0.0038	0.0030	0.0041	0.0035	0.0018
210	0.0070	0.0074	0.0183	**0.0096**	**0.0048**	0.0063	0.0009	0.0012	0.0043	0.0022
216	0.0114	0.0110	0.0106	**0.0091**	**0.0045**	0.0027	0.0048	0.0046	0.0039	0.0020
222	0.0104	0.0105	0.0085	**0.0085**	**0.0042**	0.0026	0.0040	0.0063	0.0031	0.0016
228	0.0172	0.0081	0.0053	**0.0093**	**0.0046**	0.0009	0.0011	0.0002	0.0019	0.0009
234	0.0064	0.0129	0.0113	**0.0081**	**0.0040**	0.0045	0.0060	0.0035	0.0043	0.0021
240	0.0140	0.0079	0.0095	**0.0089**	**0.0044**	0.0057	0.0032	0.0041	0.0037	0.0018
246	0.0008	0.0122	0.0078	**0.0083**	**0.0041**	0.0048	0.0042	0.0037	0.0032	0.0016
252	0.0076	0.0111	0.0064	**0.0074**	**0.0037**	0.0079	0.0025	0.0036	0.0046	0.0023
258	0.0090	0.0047	0.0054	**0.0074**	**0.0037**	0.0030	0.0021	0.0018	0.0026	0.0013
264	0.0091	0.0064	0.0016	**0.0072**	**0.0036**	0.0041	0.0023	0.0036	0.0030	0.0015
270	0.0057	0.0193	0.0098	**0.0079**	**0.0039**	0.0043	0.0037	0.0058	0.0047	0.0024
276	0.0083	0.0060	0.0007	**0.0075**	**0.0038**	0.0040	0.0000	0.0029	0.0035	0.0017
282	0.0087	-0.0044	0.0044	**0.0081**	**0.0041**	0.0014	0.0032	0.0019	0.0030	0.0015
288	0.0024	0.0078	0.0133	**0.0087**	**0.0043**	0.0057	0.0094	0.0107	0.0058	0.0029
294	0.0090	0.0055	0.0013	**0.0071**	**0.0036**	-0.0023	-0.0003	-0.0028	-0.0016	-0.0008

pH5.2

	0.5 mM Ca2+					No Ca2+				
**time**	**rep1**	**rep2**	**rep3**	**mean**	**error bars**	**rep1**	**rep2**	**rep3**	**mean**	**error bars**
0	0.0000	0.0000	0.0000	**0.0000**	**0.0000**	0.0000	0.0000	0.0000	0.0000	0.0000
6	-0.0016	-0.0040	-0.0009	**-0.0021**	**-0.0011**	-0.0011	-0.0060	-0.0044	-0.0048	-0.0024
12	-0.0034	-0.0018	-0.0055	**-0.0043**	**-0.0022**	-0.0066	-0.0043	-0.0078	-0.0058	-0.0029
18	-0.0035	-0.0020	-0.0009	**-0.0030**	**-0.0015**	-0.0056	-0.0095	-0.0107	-0.0080	-0.0040
24	0.0014	-0.0022	-0.0012	**-0.0041**	**-0.0021**	-0.0073	-0.0042	-0.0087	-0.0078	-0.0039
30	-0.0036	-0.0042	0.0000	**-0.0027**	**-0.0014**	-0.0117	0.0007	-0.0080	-0.0076	-0.0038
36	-0.0005	-0.0032	-0.0062	**-0.0042**	**-0.0021**	-0.0100	-0.0110	-0.0076	-0.0093	-0.0047
42	-0.0071	-0.0076	-0.0035	**-0.0044**	**-0.0022**	-0.0088	-0.0054	-0.0085	-0.0075	-0.0037
48	-0.0018	-0.0013	-0.0034	**-0.0026**	**-0.0013**	-0.0147	-0.0089	-0.0043	-0.0093	-0.0047
54	-0.0075	-0.0013	-0.0050	**-0.0054**	**-0.0027**	-0.0096	-0.0123	-0.0088	-0.0101	-0.0051
60	-0.0022	-0.0038	-0.0046	**-0.0029**	**-0.0015**	-0.0174	-0.0074	-0.0075	-0.0095	-0.0047
66	-0.0024	-0.0019	-0.0012	**-0.0015**	**-0.0007**	-0.0158	-0.0100	-0.0111	-0.0087	-0.0044
72	-0.0003	-0.0002	-0.0006	**-0.0004**	**-0.0002**	-0.0093	-0.0037	-0.0051	-0.0056	-0.0028
78	-0.0012	-0.0001	-0.0013	**-0.0022**	**-0.0011**	-0.0109	-0.0046	-0.0085	-0.0076	-0.0038
84	-0.0072	-0.0047	-0.0034	**-0.0042**	**-0.0021**	-0.0035	-0.0105	-0.0177	-0.0083	-0.0042
90	-0.0015	-0.0016	-0.0016	**-0.0013**	**-0.0007**	-0.0072	-0.0063	-0.0144	-0.0078	-0.0039
96	-0.0029	-0.0068	-0.0049	**-0.0032**	**-0.0016**	-0.0043	-0.0065	-0.0079	-0.0081	-0.0040
102	0.0006	-0.0014	-0.0026	**-0.0027**	**-0.0013**	-0.0049	-0.0017	-0.0019	-0.0037	-0.0019
108	-0.0038	-0.0026	-0.0030	**-0.0030**	**-0.0015**	-0.0088	-0.0070	-0.0012	-0.0077	-0.0038
114	-0.0019	-0.0008	-0.0048	**-0.0023**	**-0.0012**	-0.0007	-0.0006	-0.0049	-0.0048	-0.0024
120	-0.0030	-0.0072	-0.0018	**-0.0033**	**-0.0017**	-0.0088	-0.0095	-0.0034	-0.0072	-0.0036
126	-0.0012	-0.0005	-0.0008	**-0.0014**	**-0.0007**	0.0000	-0.0092	-0.0068	-0.0071	-0.0036
132	-0.0037	-0.0035	-0.0014	**-0.0025**	**-0.0013**	-0.0052	-0.0042	-0.0045	-0.0053	-0.0026
138	-0.0043	-0.0031	-0.0057	**-0.0040**	**-0.0020**	-0.0101	-0.0048	-0.0065	-0.0052	-0.0026
144	-0.0014	-0.0041	-0.0005	**-0.0023**	**-0.0012**	0.0004	-0.0012	-0.0028	-0.0046	-0.0023
150	-0.0003	-0.0001	-0.0002	**-0.0003**	**-0.0001**	-0.0076	-0.0084	-0.0160	-0.0071	-0.0036
156	-0.0013	-0.0051	-0.0026	**-0.0029**	**-0.0014**	-0.0007	-0.0008	-0.0053	-0.0061	-0.0031
162	-0.0016	-0.0004	-0.0007	**-0.0014**	**-0.0007**	-0.0031	-0.0026	-0.0080	-0.0054	-0.0027
168	-0.0008	-0.0006	-0.0009	**-0.0007**	**-0.0003**	-0.0051	-0.0080	-0.0071	-0.0064	-0.0032
174	-0.0019	-0.0007	-0.0006	**-0.0012**	**-0.0006**	-0.0009	-0.0065	-0.0041	-0.0047	-0.0024
180	-0.0010	-0.0008	-0.0009	**-0.0008**	**-0.0004**	-0.0034	-0.0048	-0.0062	-0.0052	-0.0026
186	-0.0015	-0.0015	-0.0034	**-0.0028**	**-0.0014**	-0.0085	-0.0057	-0.0007	-0.0054	-0.0027
192	-0.0020	-0.0009	-0.0018	**-0.0011**	**-0.0006**	-0.0073	-0.0037	-0.0099	-0.0051	-0.0026
198	-0.0031	-0.0028	-0.0033	**-0.0019**	**-0.0009**	-0.0083	-0.0084	-0.0053	-0.0064	-0.0032
204	-0.0028	-0.0005	-0.0014	**-0.0024**	**-0.0012**	-0.0026	-0.0028	-0.0016	-0.0045	-0.0022
210	-0.0011	-0.0003	-0.0009	**-0.0012**	**-0.0006**	-0.0019	-0.0052	-0.0023	-0.0033	-0.0016
216	-0.0013	-0.0005	-0.0008	**-0.0008**	**-0.0004**	-0.0054	-0.0099	-0.0042	-0.0055	-0.0028
222	-0.0021	-0.0035	-0.0016	**-0.0018**	**-0.0009**	-0.0058	-0.0087	-0.0071	-0.0065	-0.0032
228	0.0005	0.0001	0.0002	**0.0004**	**0.0002**	-0.0059	-0.0039	-0.0044	-0.0048	-0.0024
234	-0.0013	-0.0014	-0.0009	**-0.0011**	**-0.0006**	-0.0046	-0.0051	-0.0025	-0.0025	-0.0013
240	-0.0025	-0.0015	-0.0029	**-0.0022**	**-0.0011**	-0.0034	-0.0052	-0.0039	-0.0055	-0.0027
246	-0.0004	-0.0006	-0.0006	**-0.0005**	**-0.0003**	-0.0028	-0.0001	-0.0010	-0.0043	-0.0022
252	0.0014	0.0004	0.0009	**0.0008**	**0.0004**	-0.0037	-0.0026	-0.0063	-0.0038	-0.0019
258	0.0003	0.0023	0.0031	**0.0022**	**0.0011**	-0.0002	-0.0015	-0.0035	-0.0028	-0.0014
264	-0.0016	-0.0031	-0.0056	**-0.0035**	**-0.0017**	-0.0065	-0.0041	-0.0026	-0.0053	-0.0026
270	-0.0024	-0.0013	-0.0023	**-0.0013**	**-0.0007**	0.0003	-0.0011	-0.0018	-0.0015	-0.0007
276	-0.0008	-0.0005	-0.0008	**-0.0009**	**-0.0004**	-0.0020	-0.0007	-0.0032	-0.0021	-0.0011
282	-0.0002	-0.0011	-0.0013	**-0.0018**	**-0.0009**	-0.0041	-0.0027	-0.0031	-0.0030	-0.0015
288	0.0023	0.0015	0.0028	**0.0014**	**0.0007**	-0.0008	-0.0008	-0.0011	-0.0008	-0.0004
294	0.0008	0.0007	0.0006	**0.0006**	**0.0003**	-0.0034	-0.0075	-0.0018	-0.0042	-0.0021

B. Ca2+ titration

time	0.1 mM Ca2+				
	rep1	rep2	rep3	mean	error
0	0.0000	0.0000	0.0000	**0.0000**	**0.0000**
6	0.0307	0.0312	0.0313	**0.0319**	**0.0016**
12	0.0507	0.0466	0.0508	**0.0467**	**0.0022**
18	0.0704	0.0620	0.0696	**0.0606**	**0.0051**
24	0.0895	0.0798	0.0837	**0.0846**	**0.0058**
30	0.1102	0.1060	0.0982	**0.1050**	**0.0072**
36	0.1048	0.1020	0.1107	**0.1130**	**0.0070**
42	0.1121	0.1169	0.1241	**0.1205**	**0.0079**
48	0.1412	0.1345	0.1335	**0.1330**	**0.0069**
54	0.1362	0.1406	0.1316	**0.1411**	**0.0092**
60	0.1448	0.1369	0.1442	**0.1451**	**0.0110**
66	0.1677	0.1598	0.1555	**0.1512**	**0.0099**
72	0.1701	0.1470	0.1556	**0.1576**	**0.0104**
78	0.1760	0.1690	0.1508	**0.1613**	**0.0115**
84	0.1513	0.1608	0.1548	**0.1633**	**0.0100**
90	0.1734	0.1688	0.1549	**0.1642**	**0.0110**
96	0.1508	0.1733	0.1711	**0.1657**	**0.0132**
102	0.1476	0.1653	0.1647	**0.1685**	**0.0104**
108	0.1740	0.1512	0.1617	**0.1713**	**0.0105**
114	0.1680	0.1803	0.1595	**0.1739**	**0.0122**
120	0.1688	0.1665	0.1663	**0.1753**	**0.0119**
126	0.1882	0.1755	0.1703	**0.1752**	**0.0137**
132	0.1769	0.1717	0.1684	**0.1755**	**0.0123**
138	0.1908	0.1774	0.1720	**0.1753**	**0.0140**
144	0.1753	0.1689	0.1851	**0.1741**	**0.0119**
150	0.1788	0.1798	0.1659	**0.1743**	**0.0135**
156	0.1963	0.1706	0.1628	**0.1733**	**0.0143**
162	0.1636	0.1914	0.1736	**0.1732**	**0.0129**
168	0.1928	0.2104	0.1429	**0.1776**	**0.0139**
174	0.1785	0.1797	0.1690	**0.1773**	**0.0138**
180	0.1757	0.1635	0.1856	**0.1708**	**0.0133**
186	0.1620	0.1635	0.1677	**0.1664**	**0.0147**
192	0.1467	0.1610	0.1471	**0.1648**	**0.0126**
198	0.1621	0.1852	0.1603	**0.1654**	**0.0125**

time	0.25 mM Ca2+				
	rep1	rep2	rep3	mean	error
0	0.0000	0.0000	0.0000	**0.0000**	**0.0000**
6	0.0337	0.0304	0.0315	**0.0325**	**0.0016**
12	0.0594	0.0567	0.0571	**0.0602**	**0.0022**
18	0.0819	0.0819	0.0848	**0.0801**	**0.0051**
24	0.0975	0.0909	0.0952	**0.0953**	**0.0058**
30	0.1049	0.1163	0.0991	**0.1101**	**0.0072**
36	0.1315	0.1290	0.1309	**0.1255**	**0.0070**
42	0.1354	0.1415	0.1396	**0.1399**	**0.0079**
48	0.1379	0.1518	0.1515	**0.1484**	**0.0069**
54	0.1439	0.1621	0.1345	**0.1507**	**0.0092**
60	0.1443	0.1446	0.1410	**0.1489**	**0.0110**
66	0.1398	0.1511	0.1378	**0.1464**	**0.0099**
72	0.1449	0.1398	0.1428	**0.1442**	**0.0104**
78	0.1653	0.1367	0.1532	**0.1430**	**0.0115**
84	0.1448	0.1447	0.1254	**0.1444**	**0.0100**
90	0.1601	0.1506	0.1441	**0.1489**	**0.0110**
96	0.1538	0.1519	0.1776	**0.1533**	**0.0132**
102	0.1434	0.1491	0.1577	**0.1550**	**0.0104**
108	0.1736	0.1568	0.1658	**0.1541**	**0.0105**
114	0.1629	0.1625	0.1695	**0.1538**	**0.0122**
120	0.1817	0.1562	0.1510	**0.1544**	**0.0119**
126	0.1592	0.1525	0.1249	**0.1566**	**0.0137**
132	0.1588	0.1623	0.1557	**0.1603**	**0.0123**
138	0.1596	0.1799	0.1638	**0.1626**	**0.0140**
144	0.1633	0.1558	0.1571	**0.1637**	**0.0119**
150	0.1612	0.1643	0.1678	**0.1626**	**0.0135**
156	0.1844	0.1804	0.1439	**0.1610**	**0.0143**
162	0.1825	0.1470	0.1931	**0.1598**	**0.0129**
168	0.1624	0.1536	0.1315	**0.1597**	**0.0139**
174	0.1528	0.1797	0.1526	**0.1583**	**0.0138**
180	0.1509	0.1395	0.1413	**0.1569**	**0.0133**
186	0.1473	0.1611	0.1254	**0.1558**	**0.0147**
192	0.1660	0.1515	0.1677	**0.1576**	**0.0126**
198	0.1605	0.1550	0.1668	**0.1605**	**0.0125**

time	0.5 mM Ca2+				
	rep1	rep2	rep3	mean	error
0	0.0000	0.0000	0.0000	**0.0000**	**0.0000**
6	0.0367	0.0359	0.0369	**0.0378**	**0.0016**
12	0.0617	0.0619	0.0618	**0.0629**	**0.0022**
18	0.0726	0.0740	0.0774	**0.0752**	**0.0051**
24	0.0909	0.0824	0.0951	**0.0845**	**0.0058**
30	0.1012	0.1037	0.0878	**0.0949**	**0.0072**
36	0.0956	0.1107	0.1108	**0.1084**	**0.0070**
42	0.1125	0.1142	0.1287	**0.1259**	**0.0079**
48	0.1479	0.1501	0.1343	**0.1407**	**0.0069**
54	0.1539	0.1540	0.1481	**0.1484**	**0.0092**
60	0.1388	0.1382	0.1585	**0.1504**	**0.0110**
66	0.1328	0.1465	0.1508	**0.1494**	**0.0099**
72	0.1603	0.1453	0.1345	**0.1490**	**0.0104**
78	0.1520	0.1397	0.1685	**0.1480**	**0.0115**
84	0.1371	0.1475	0.1662	**0.1458**	**0.0100**
90	0.1594	0.1476	0.1531	**0.1478**	**0.0110**
96	0.1855	0.1722	0.1361	**0.1537**	**0.0132**
102	0.1588	0.1680	0.1409	**0.1569**	**0.0104**
108	0.1595	0.1558	0.1595	**0.1565**	**0.0105**
114	0.1457	0.1333	0.1425	**0.1555**	**0.0122**
120	0.1786	0.1548	0.1450	**0.1563**	**0.0119**
126	0.1787	0.1712	0.1467	**0.1586**	**0.0137**
132	0.1751	0.1755	0.1522	**0.1618**	**0.0123**
138	0.1721	0.1593	0.1820	**0.1645**	**0.0140**
144	0.1505	0.1606	0.1752	**0.1655**	**0.0119**
150	0.1678	0.1767	0.1924	**0.1689**	**0.0135**
156	0.1688	0.1592	0.1972	**0.1749**	**0.0143**
162	0.1711	0.1809	0.1693	**0.1768**	**0.0129**
168	0.1547	0.1634	0.1949	**0.1766**	**0.0139**
174	0.1719	0.1756	0.1818	**0.1777**	**0.0138**
180	0.1942	0.1806	0.1751	**0.1786**	**0.0133**
186	0.1690	0.1902	0.1508	**0.1816**	**0.0147**
192	0.1700	0.1940	0.1904	**0.1848**	**0.0126**
198	0.1678	0.1905	0.1896	**0.1838**	**0.0125**

time	1 mM Ca^{2+} rep1	rep2	rep3	mean	error
0	0.0000	0.0000	0.0000	**0.0000**	**0.0000**
6	0.0443	0.0434	0.0451	**0.0449**	**0.0011**
12	0.0599	0.0603	0.0615	**0.0608**	**0.0016**
18	0.0722	0.0711	0.0687	**0.0724**	**0.0036**
24	0.0701	0.0693	0.0710	**0.0735**	**0.0041**
30	0.0851	0.0768	0.0732	**0.0760**	**0.0052**
36	0.0759	0.0778	0.0779	**0.0766**	**0.0050**
42	0.0798	0.0841	0.0761	**0.0780**	**0.0056**
48	0.0790	0.0741	0.0680	**0.0772**	**0.0049**
54	0.0754	0.0885	0.0769	**0.0792**	**0.0066**
60	0.0872	0.0839	0.0745	**0.0792**	**0.0079**
66	0.0940	0.0867	0.0869	**0.0805**	**0.0070**
72	0.0738	0.0758	0.0853	**0.0805**	**0.0074**
78	0.0897	0.0796	0.0939	**0.0798**	**0.0082**
84	0.0781	0.0842	0.0791	**0.0791**	**0.0071**
90	0.0878	0.0766	0.0789	**0.0791**	**0.0079**
96	0.0645	0.0816	0.0796	**0.0788**	**0.0094**
102	0.0844	0.0830	0.0755	**0.0805**	**0.0074**
108	0.0856	0.1002	0.0757	**0.0806**	**0.0075**
114	0.0874	0.0742	0.0713	**0.0792**	**0.0087**
120	0.0703	0.0847	0.0801	**0.0798**	**0.0085**
126	0.0884	0.0780	0.0683	**0.0799**	**0.0098**
132	0.0777	0.0788	0.0849	**0.0795**	**0.0088**
138	0.0682	0.0619	0.0849	**0.0803**	**0.0100**
144	0.0733	0.0757	0.0845	**0.0799**	**0.0085**
150	0.0802	0.0816	0.0914	**0.0800**	**0.0096**
156	0.0913	0.0804	0.0749	**0.0804**	**0.0102**
162	0.0693	0.0832	0.1017	**0.0803**	**0.0092**
168	0.0912	0.0831	0.0699	**0.0808**	**0.0100**
174	0.0681	0.0750	0.0432	**0.0803**	**0.0099**
180	0.0841	0.0919	0.0811	**0.0801**	**0.0095**
186	0.0766	0.0943	0.0755	**0.0800**	**0.0105**
192	0.0760	0.0895	0.0812	**0.0812**	**0.0090**
198	0.0733	0.0866	0.0747	**0.0790**	**0.0089**

time	2 mM Ca^{2+} rep1	rep2	rep3	mean	error
0	0.0023	-0.0010	-0.0009	**0.0000**	**0.0015**
6	0.0276	0.0287	0.0297	**0.0283**	**0.0011**
12	0.0373	0.0379	0.0387	**0.0367**	**0.0016**
18	0.0389	0.0428	0.0389	**0.0393**	**0.0036**
24	0.0503	0.0427	0.0394	**0.0416**	**0.0041**
30	0.0443	0.0400	0.0359	**0.0438**	**0.0052**
36	0.0455	0.0449	0.0470	**0.0437**	**0.0050**
42	0.0406	0.0408	0.0426	**0.0454**	**0.0056**
48	0.0402	0.0584	0.0470	**0.0451**	**0.0049**
54	0.0413	0.0484	0.0536	**0.0456**	**0.0066**
60	0.0508	0.0527	0.0493	**0.0455**	**0.0079**
66	0.0360	0.0412	0.0541	**0.0466**	**0.0070**
72	0.0453	0.0487	0.0509	**0.0468**	**0.0074**
78	0.0391	0.0491	0.0439	**0.0475**	**0.0082**
84	0.0356	0.0489	0.0401	**0.0461**	**0.0071**
90	0.0436	0.0370	0.0428	**0.0476**	**0.0079**
96	0.0498	0.0605	0.0350	**0.0456**	**0.0094**
102	0.0495	0.0508	0.0547	**0.0469**	**0.0074**
108	0.0567	0.0389	0.0477	**0.0472**	**0.0075**
114	0.0575	0.0515	0.0589	**0.0467**	**0.0087**
120	0.0587	0.0683	0.0261	**0.0484**	**0.0085**
126	0.0430	0.0430	0.0323	**0.0463**	**0.0098**
132	0.0394	0.0566	0.0446	**0.0440**	**0.0088**
138	0.0536	0.0603	0.0411	**0.0440**	**0.0100**
144	0.0423	0.0346	0.0435	**0.0458**	**0.0085**
150	0.0410	0.0458	0.0457	**0.0479**	**0.0096**
156	0.0405	0.0362	0.0441	**0.0479**	**0.0102**
162	0.0474	0.0390	0.0506	**0.0473**	**0.0092**
168	0.0457	0.0446	0.0562	**0.0471**	**0.0100**
174	0.0700	0.0556	0.0536	**0.0472**	**0.0099**
180	0.0478	0.0490	0.0533	**0.0478**	**0.0095**
186	0.0414	0.0315	0.0518	**0.0473**	**0.0105**
192	0.0417	0.0516	0.0406	**0.0480**	**0.0090**
198	0.0360	0.0346	0.0497	**0.0472**	**0.0089**

time	5 mM Ca^{2+} rep1	rep2	rep3	mean	error
0	0.0000	0.0000	0.0000	**0.0000**	**0.0000**
6	0.0228	0.0229	0.0253	**0.0227**	**0.0011**
12	0.0304	0.0314	0.0306	**0.0317**	**0.0016**
18	0.0376	0.0359	0.0338	**0.0350**	**0.0036**
24	0.0401	0.0328	0.0375	**0.0370**	**0.0041**
30	0.0390	0.0461	0.0407	**0.0380**	**0.0052**
36	0.0394	0.0326	0.0319	**0.0393**	**0.0050**
42	0.0431	0.0358	0.0384	**0.0397**	**0.0056**
48	0.0349	0.0401	0.0403	**0.0392**	**0.0049**
54	0.0519	0.0524	0.0474	**0.0405**	**0.0066**
60	0.0494	0.0317	0.0509	**0.0409**	**0.0079**
66	0.0342	0.0295	0.0631	**0.0408**	**0.0070**
72	0.0433	0.0360	0.0410	**0.0409**	**0.0074**
78	0.0416	0.0239	0.0351	**0.0410**	**0.0082**
84	0.0444	0.0409	0.0288	**0.0406**	**0.0071**
90	0.0384	0.0411	0.0429	**0.0423**	**0.0079**
96	0.0246	0.0429	0.0549	**0.0405**	**0.0094**
102	0.0289	0.0440	0.0508	**0.0407**	**0.0074**
108	0.0459	0.0475	0.0395	**0.0430**	**0.0075**
114	0.0411	0.0327	0.0416	**0.0427**	**0.0087**
120	0.0365	0.0356	0.0535	**0.0412**	**0.0085**
126	0.0396	0.0469	0.0444	**0.0408**	**0.0098**
132	0.0322	0.0245	0.0285	**0.0412**	**0.0088**
138	0.0440	0.0382	0.0488	**0.0412**	**0.0100**
144	0.0376	0.0296	0.0416	**0.0408**	**0.0085**
150	0.0528	0.0447	0.0404	**0.0422**	**0.0096**
156	0.0381	0.0554	0.0518	**0.0424**	**0.0102**
162	0.0382	0.0297	0.0337	**0.0421**	**0.0092**
168	0.0464	0.0322	0.0392	**0.0430**	**0.0100**
174	0.0407	0.0354	0.0357	**0.0419**	**0.0099**
180	0.0396	0.0365	0.0291	**0.0418**	**0.0095**
186	0.0498	0.0323	0.0529	**0.0420**	**0.0105**
192	0.0387	0.0391	0.0535	**0.0418**	**0.0090**
198	0.0428	0.0368	0.0331	**0.0404**	**0.0089**

time	10 mM Ca2+				
	rep1	rep2	rep3	mean	error
0	0.0000	0.0000	0.0000	**0.0000**	**0.0000**
6	0.0199	0.0193	0.0203	**0.0199**	**0.0011**
12	0.0291	0.0274	0.0303	**0.0281**	**0.0016**
18	0.0304	0.0349	0.0352	**0.0317**	**0.0036**
24	0.0339	0.0333	0.0373	**0.0337**	**0.0041**
30	0.0255	0.0350	0.0352	**0.0349**	**0.0052**
36	0.0372	0.0310	0.0284	**0.0354**	**0.0050**
42	0.0217	0.0396	0.0425	**0.0365**	**0.0056**
48	0.0345	0.0485	0.0336	**0.0367**	**0.0049**
54	0.0293	0.0369	0.0386	**0.0379**	**0.0066**
60	0.0432	0.0388	0.0318	**0.0378**	**0.0079**
66	0.0279	0.0345	0.0291	**0.0387**	**0.0070**
72	0.0440	0.0378	0.0249	**0.0386**	**0.0074**
78	0.0410	0.0304	0.0491	**0.0380**	**0.0082**
84	0.0380	0.0393	0.0320	**0.0377**	**0.0071**
90	0.0427	0.0391	0.0403	**0.0386**	**0.0079**
96	0.0345	0.0436	0.0145	**0.0375**	**0.0094**
102	0.0397	0.0463	0.0370	**0.0384**	**0.0074**
108	0.0436	0.0348	0.0405	**0.0392**	**0.0075**
114	0.0336	0.0486	0.0427	**0.0386**	**0.0087**
120	0.0504	0.0384	0.0383	**0.0388**	**0.0085**
126	0.0415	0.0527	0.0150	**0.0385**	**0.0098**
132	0.0236	0.0496	0.0551	**0.0384**	**0.0088**
138	0.0369	0.0525	0.0607	**0.0390**	**0.0100**
144	0.0395	0.0341	0.0339	**0.0379**	**0.0085**
150	0.0363	0.0464	0.0371	**0.0387**	**0.0096**
156	0.0601	0.0358	0.0319	**0.0392**	**0.0102**
162	0.0438	0.0410	0.0431	**0.0388**	**0.0092**
168	0.0178	0.0372	0.0204	**0.0387**	**0.0100**
174	0.0409	0.0466	0.0507	**0.0390**	**0.0099**
180	0.0460	0.0435	0.0462	**0.0389**	**0.0095**
186	0.0382	0.0384	0.0495	**0.0388**	**0.0105**
192	0.0485	0.0345	0.0226	**0.0388**	**0.0090**
198	0.0183	0.0243	0.0298	**0.0388**	**0.0089**

C. Alternative cations

pH5.2

	0.5 mM Zn2+					0.5 mM Mg2+				
time	rep1	rep2	rep3	mean	error bars	rep1	rep2	rep3	mean	error bars
0	0.0000	0.0000	0.0000	**0.0000**	**0.0000**	0.0000	0.0000	0.0000	0.0000	0.0000
2	0.0001	-0.0032	0.0010	**0.0005**	**0.0068**	0.0258	0.0174	0.0274	0.0226	0.0036
4	0.0132	0.0194	-0.0007	**0.0081**	**0.0099**	0.0145	0.0193	0.0158	0.0181	0.0050
6	0.0083	0.0077	0.0140	**0.0106**	**0.0040**	0.0257	0.0150	0.0168	0.0221	0.0050
8	0.0363	0.0300	0.0335	**0.0170**	**0.0123**	0.0278	0.0203	0.0211	0.0231	0.0029
10	0.0126	0.0063	0.0152	**0.0111**	**0.0060**	0.0287	0.0229	0.0292	0.0283	0.0067
12	0.0262	0.0214	0.0231	**0.0194**	**0.0039**	0.0376	0.0285	0.0422	0.0279	0.0083
14	0.0052	0.0215	0.0159	**0.0171**	**0.0077**	0.0034	0.0144	0.0088	0.0171	0.0095
16	0.0212	0.0280	0.0099	**0.0160**	**0.0095**	0.0291	0.0146	0.0102	0.0259	0.0089
18	0.0406	0.0186	0.0274	**0.0271**	**0.0090**	0.0303	0.0238	0.0280	0.0321	0.0046
20	0.0273	0.0361	0.0200	**0.0238**	**0.0116**	0.0255	0.0188	0.0322	0.0288	0.0060
22	0.0088	0.0174	0.0135	**0.0122**	**0.0037**	0.0325	0.0301	0.0281	0.0317	0.0025
24	0.0231	0.0260	0.0234	**0.0256**	**0.0013**	0.0284	0.0291	0.0161	0.0302	0.0065
26	0.0298	0.0339	0.0231	**0.0244**	**0.0069**	0.0428	0.0399	0.0328	0.0385	0.0033
28	0.0184	0.0268	0.0199	**0.0219**	**0.0039**	0.0510	0.0375	0.0291	0.0385	0.0063
30	0.0190	0.0165	0.0154	**0.0236**	**0.0056**	0.0425	0.0484	0.0400	0.0471	0.0049
32	0.0281	0.0235	0.0321	**0.0260**	**0.0077**	0.0459	0.0477	0.0462	0.0481	0.0047
34	0.0310	0.0292	0.0108	**0.0317**	**0.0121**	0.0454	0.0361	0.0408	0.0434	0.0102
36	0.0284	0.0306	0.0292	**0.0303**	**0.0025**	0.0447	0.0080	0.0126	0.0418	0.0152
38	0.0388	0.0270	0.0333	**0.0340**	**0.0038**	0.0499	0.0543	0.0548	0.0540	0.0039
40	0.0282	0.0220	0.0282	**0.0274**	**0.0075**	0.0334	0.0595	0.0572	0.0428	0.0128
42	0.0489	0.0179	0.0259	**0.0299**	**0.0114**	0.0380	0.0667	0.0364	0.0394	0.0082
44	0.0189	0.0269	0.0197	**0.0242**	**0.0037**	0.0458	0.0356	0.0194	0.0362	0.0097
46	0.0351	0.0496	0.0458	**0.0406**	**0.0064**	0.0601	0.0500	0.0539	0.0536	0.0034
48	0.0232	0.0398	0.0241	**0.0303**	**0.0094**	0.0685	0.0525	0.0491	0.0444	0.0125
50	0.0482	0.0617	0.0650	**0.0474**	**0.0078**	0.0654	0.0657	0.0668	0.0655	0.0007
52	0.0375	0.0429	0.0570	**0.0433**	**0.0131**	0.0416	0.0439	0.0417	0.0459	0.0093
54	0.0512	0.0443	0.0445	**0.0462**	**0.0082**	0.0541	0.0566	0.0650	0.0584	0.0106
56	0.0442	0.0281	0.0510	**0.0527**	**0.0108**	0.0585	0.0475	0.0788	0.0573	0.0130
58	0.0408	0.0420	0.0455	**0.0423**	**0.0013**	0.0470	0.0506	0.0533	0.0527	0.0066
60	0.0446	0.0622	0.0309	**0.0442**	**0.0119**	0.0377	0.0434	0.0410	0.0520	0.0085
62	0.0497	0.0309	0.0398	**0.0416**	**0.0064**	0.0278	0.0746	0.0466	0.0538	0.0098
64	0.0468	0.0481	0.0452	**0.0457**	**0.0038**	0.0586	0.0558	0.0523	0.0557	0.0035
66	0.0339	0.0331	0.0360	**0.0402**	**0.0058**	0.0590	0.0599	0.0567	0.0591	0.0021
68	0.0376	0.0395	0.0379	**0.0419**	**0.0042**	0.0720	0.0720	0.0673	0.0677	0.0036
70	0.0447	0.0380	0.0380	**0.0361**	**0.0069**	0.0607	0.0458	0.0604	0.0557	0.0082
72	0.0417	0.0531	0.0536	**0.0503**	**0.0102**	0.0553	0.0670	0.0497	0.0562	0.0067
74	0.0328	0.0302	0.0228	**0.0421**	**0.0100**	0.0751	0.0559	0.0600	0.0557	0.0096
76	0.0583	0.0403	0.0504	**0.0499**	**0.0096**	0.0790	0.0744	0.0482	0.0598	0.0132
78	0.0345	0.0346	0.0327	**0.0343**	**0.0026**	0.0520	0.0520	0.0661	0.0585	0.0071
80	0.0342	0.0336	0.0462	**0.0359**	**0.0076**	0.0487	0.0451	0.0513	0.0504	0.0119
82	0.0451	0.0476	0.0509	**0.0481**	**0.0026**	0.0715	0.0596	0.0643	0.0612	0.0099
84	0.0542	0.0470	0.0559	**0.0522**	**0.0048**	0.0430	0.0537	0.0511	0.0580	0.0065
86	0.0244	0.0369	0.0486	**0.0397**	**0.0057**	0.0427	0.0421	0.0439	0.0463	0.0055
88	0.0548	0.0569	0.0505	**0.0512**	**0.0072**	0.0662	0.0719	0.0758	0.0686	0.0050
90	0.0458	0.0480	0.0513	**0.0466**	**0.0021**	0.0420	0.0747	0.0700	0.0572	0.0099
92	0.0318	0.0345	0.0314	**0.0322**	**0.0015**	0.0494	0.0622	0.0507	0.0486	0.0086
94	0.0504	0.0545	0.0487	**0.0520**	**0.0037**	0.0575	0.0687	0.0745	0.0628	0.0052
96	0.0480	0.0328	0.0285	**0.0425**	**0.0058**	0.0354	0.0329	0.0567	0.0440	0.0111
98	0.0474	0.0508	0.0573	**0.0485**	**0.0068**	0.0656	0.0548	0.0759	0.0658	0.0081
100	0.0478	0.0558	0.0490	**0.0466**	**0.0096**	0.0608	0.0545	0.0566	0.0623	0.0099
102	0.0561	0.0383	0.0479	**0.0488**	**0.0108**	0.0463	0.0679	0.0364	0.0538	0.0151
104	0.0388	0.0414	0.0598	**0.0431**	**0.0073**	0.0679	0.0735	0.0540	0.0630	0.0105
106	0.0440	0.0516	0.0498	**0.0515**	**0.0053**	0.0648	0.0533	0.0669	0.0628	0.0058
108	0.0577	0.0737	0.0638	**0.0534**	**0.0113**	0.0505	0.0688	0.0653	0.0612	0.0095
110	0.0492	0.0397	0.0677	**0.0573**	**0.0123**	0.0634	0.0531	0.0802	0.0674	0.0097
112	0.0452	0.0471	0.0553	**0.0521**	**0.0070**	0.0712	0.0825	0.0701	0.0701	0.0074
114	0.0409	0.0617	0.0519	**0.0512**	**0.0068**	0.0450	0.0852	0.0623	0.0656	0.0116

116	0.0480	0.0596	0.0596	**0.0506**	**0.0112**	0.0714	0.0669	0.0706	0.0689	0.0027
118	0.0411	0.0458	0.0432	**0.0420**	**0.0034**	0.0733	0.0753	0.0696	0.0641	0.0110
120	0.0611	0.0566	0.0542	**0.0552**	**0.0053**	0.0668	0.0687	0.0704	0.0672	0.0105
122	0.0490	0.0408	0.0454	**0.0433**	**0.0032**	0.0604	0.0702	0.0579	0.0660	0.0076
124	0.0812	0.0491	0.0629	**0.0545**	**0.0132**	0.0607	0.0738	0.0740	0.0727	0.0063
126	0.0602	0.0531	0.0568	**0.0568**	**0.0107**	0.0668	0.0843	0.0782	0.0643	0.0113
128	0.0489	0.0555	0.0499	**0.0465**	**0.0039**	0.0819	0.0667	0.0731	0.0646	0.0093
130	0.0635	0.0467	0.0682	**0.0658**	**0.0097**	0.0689	0.0722	0.0585	0.0742	0.0090
132	0.0607	0.0505	0.0680	**0.0582**	**0.0097**	0.0684	0.0810	0.0518	0.0674	0.0147
134	0.0559	0.0589	0.0423	**0.0498**	**0.0155**	0.0600	0.0544	0.0534	0.0625	0.0112
136	0.0457	0.0465	0.0442	**0.0482**	**0.0036**	0.0619	0.0599	0.0615	0.0597	0.0024
138	0.0496	0.0581	0.0378	**0.0531**	**0.0068**	0.0704	0.0745	0.0504	0.0674	0.0075
140	0.0409	0.0495	0.0509	**0.0493**	**0.0048**	0.0676	0.0726	0.0756	0.0736	0.0059
142	0.0516	0.0653	0.0720	**0.0604**	**0.0092**	0.0750	0.0629	0.0632	0.0673	0.0068
144	0.0557	0.0513	0.0529	**0.0544**	**0.0020**	0.0598	0.0695	0.0637	0.0626	0.0100
146	0.0443	0.0448	0.0445	**0.0450**	**0.0009**	0.0748	0.0551	0.0674	0.0631	0.0104
148	0.0410	0.0851	0.0492	**0.0545**	**0.0160**	0.0774	0.0724	0.0772	0.0765	0.0025
150	0.0583	0.0645	0.0361	**0.0605**	**0.0141**	0.0864	0.0470	0.0546	0.0631	0.0134
152	0.0469	0.0620	0.0609	**0.0576**	**0.0093**	0.0538	0.0713	0.0576	0.0621	0.0081
154	0.0484	0.0645	0.0561	**0.0566**	**0.0070**	0.0727	0.0553	0.0540	0.0672	0.0073
156	0.0548	0.0545	0.0480	**0.0534**	**0.0098**	0.0734	0.0707	0.0686	0.0667	0.0067
158	0.0558	0.0660	0.0574	**0.0668**	**0.0060**	0.0807	0.0696	0.0705	0.0687	0.0123
160	0.0501	0.0533	0.0532	**0.0515**	**0.0084**	0.0602	0.0646	0.0591	0.0645	0.0049
162	0.0368	0.0543	0.0386	**0.0507**	**0.0100**	0.0654	0.0687	0.0736	0.0725	0.0079
164	0.0487	0.0511	0.0578	**0.0470**	**0.0068**	0.0768	0.0742	0.0743	0.0734	0.0021
166	0.0440	0.0653	0.0579	**0.0591**	**0.0074**	0.0759	0.0857	0.0764	0.0678	0.0148
168	0.0547	0.0543	0.0549	**0.0548**	**0.0008**	0.0665	0.0651	0.0680	0.0663	0.0028
170	0.0455	0.0431	0.0436	**0.0472**	**0.0066**	0.0315	0.0559	0.0687	0.0554	0.0142
172	0.0504	0.0499	0.0522	**0.0481**	**0.0037**	0.0599	0.0696	0.0647	0.0627	0.0121
174	0.0582	0.0548	0.0483	**0.0558**	**0.0066**	0.0743	0.0671	0.0816	0.0704	0.0067
176	0.0530	0.0515	0.0528	**0.0522**	**0.0024**	0.0813	0.0806	0.0771	0.0802	0.0026
178	0.0514	0.0474	0.0475	**0.0489**	**0.0029**	0.0707	0.0581	0.0697	0.0726	0.0100
180	0.0570	0.0625	0.0841	**0.0600**	**0.0118**	0.0605	0.0660	0.0671	0.0663	0.0047
182	0.0447	0.0674	0.0514	**0.0536**	**0.0103**	0.0639	0.0820	0.0797	0.0680	0.0085
184	0.0676	0.0599	0.0545	**0.0594**	**0.0092**	0.0714	0.0685	0.0692	0.0665	0.0035
186	0.0575	0.0724	0.0761	**0.0607**	**0.0122**	0.0651	0.0716	0.0614	0.0705	0.0081
188	0.0616	0.0550	0.0518	**0.0574**	**0.0043**	0.0650	0.0705	0.0569	0.0636	0.0061
190	0.0551	0.0558	0.0398	**0.0530**	**0.0053**	0.0709	0.0682	0.0726	0.0713	0.0021
192	0.0587	0.0749	0.0571	**0.0585**	**0.0078**	0.0785	0.0780	0.0665	0.0719	0.0053
194	0.0494	0.0455	0.0371	**0.0561**	**0.0105**	0.0616	0.0655	0.0636	0.0662	0.0065
196	0.0537	0.0577	0.0513	**0.0562**	**0.0051**	0.0669	0.0638	0.0687	0.0677	0.0015
198	0.0589	0.0649	0.0493	**0.0560**	**0.0113**	0.0618	0.0661	0.0641	0.0629	0.0046
200	0.0560	0.0569	0.0501	**0.0506**	**0.0111**	0.0394	0.0597	0.0679	0.0620	0.0076
202	0.0853	0.0541	0.0690	**0.0664**	**0.0096**	0.0838	0.0749	0.0804	0.0770	0.0065
204	0.0467	0.0479	0.0544	**0.0519**	**0.0056**	0.0667	0.0765	0.0913	0.0634	0.0142
206	0.0649	0.0572	0.0666	**0.0558**	**0.0082**	0.0717	0.0913	0.0790	0.0776	0.0065
208	0.0415	0.0582	0.0468	**0.0537**	**0.0131**	0.0620	0.0618	0.0720	0.0707	0.0110
210	0.0425	0.0284	0.0538	**0.0481**	**0.0075**	0.0744	0.0749	0.0758	0.0741	0.0021
212	0.0697	0.0617	0.0668	**0.0588**	**0.0058**	0.0726	0.0594	0.0626	0.0692	0.0095
214	0.0525	0.0456	0.0475	**0.0521**	**0.0053**	0.0529	0.0539	0.0590	0.0571	0.0054
216	0.0440	0.0685	0.0481	**0.0543**	**0.0129**	0.0764	0.0750	0.0732	0.0756	0.0023
218	0.0497	0.0460	0.0490	**0.0495**	**0.0027**	0.0625	0.0727	0.0647	0.0661	0.0111
220	0.0362	0.0495	0.0707	**0.0597**	**0.0145**	0.0829	0.0805	0.0817	0.0814	0.0011
222	0.0715	0.0590	0.0551	**0.0541**	**0.0102**	0.0789	0.0648	0.0704	0.0708	0.0070
224	0.0593	0.0566	0.0319	**0.0510**	**0.0078**	0.0608	0.0620	0.0598	0.0660	0.0043
226	0.0468	0.0492	0.0491	**0.0513**	**0.0025**	0.0775	0.0821	0.0799	0.0808	0.0035
228	0.0543	0.0608	0.0590	**0.0657**	**0.0071**	0.0750	0.0788	0.0731	0.0700	0.0086
230	0.0688	0.0472	0.0388	**0.0549**	**0.0126**	0.0745	0.0744	0.0864	0.0687	0.0075
232	0.0511	0.0481	0.0584	**0.0556**	**0.0109**	0.0745	0.0616	0.0718	0.0701	0.0057
234	0.0550	0.0488	0.0591	**0.0562**	**0.0050**	0.0718	0.0742	0.0731	0.0661	0.0085
236	0.0531	0.0512	0.0493	**0.0559**	**0.0077**	0.0672	0.0718	0.0747	0.0716	0.0089
238	0.0610	0.0543	0.0435	**0.0551**	**0.0074**	0.0662	0.0733	0.0591	0.0654	0.0039
240	0.0599	0.0518	0.0538	**0.0529**	**0.0032**	0.0737	0.0762	0.0627	0.0672	0.0076

242	0.0594	0.0372	0.0709	**0.0546**	**0.0133**	0.0615	0.0557	0.0464	0.0566	0.0099
244	0.0575	0.0571	0.0620	**0.0565**	**0.0041**	0.0645	0.0725	0.0619	0.0640	0.0051
246	0.0606	0.0547	0.0601	**0.0601**	**0.0031**	0.0813	0.0668	0.0684	0.0761	0.0083
248	0.0480	0.0481	0.0462	**0.0457**	**0.0019**	0.0461	0.0432	0.0504	0.0643	0.0147
250	0.0601	0.0712	0.0666	**0.0669**	**0.0040**	0.0653	0.0685	0.0603	0.0715	0.0068
252	0.0563	0.0609	0.0572	**0.0554**	**0.0043**	0.0643	0.0627	0.0626	0.0673	0.0058
254	0.0140	0.0364	0.0454	**0.0544**	**0.0108**	0.0668	0.0794	0.0695	0.0695	0.0084
256	0.0383	0.0513	0.0513	**0.0499**	**0.0090**	0.0658	0.0735	0.0676	0.0630	0.0066
258	0.0480	0.0664	0.0470	**0.0548**	**0.0099**	0.0707	0.0749	0.0800	0.0702	0.0044
260	0.0571	0.0538	0.0515	**0.0605**	**0.0100**	0.0645	0.0831	0.0605	0.0716	0.0136
262	0.0611	0.0451	0.0571	**0.0605**	**0.0110**	0.0630	0.0655	0.0681	0.0684	0.0043
264	0.0711	0.0710	0.0479	**0.0642**	**0.0084**	0.0547	0.0588	0.0764	0.0771	0.0114
266	0.0678	0.0601	0.0682	**0.0642**	**0.0089**	0.0776	0.0792	0.0762	0.0723	0.0087
268	0.0558	0.0519	0.0543	**0.0544**	**0.0081**	0.0687	0.0767	0.0990	0.0707	0.0114
270	0.0610	0.0616	0.0543	**0.0580**	**0.0050**	0.0922	0.0694	0.0859	0.0701	0.0112
272	0.0415	0.0493	0.0617	**0.0529**	**0.0086**	0.0544	0.0667	0.0641	0.0623	0.0127
274	0.0506	0.0508	0.0480	**0.0495**	**0.0034**	0.0774	0.0521	0.0756	0.0676	0.0128
276	0.0489	0.0641	0.0524	**0.0530**	**0.0054**	0.0620	0.0633	0.0520	0.0606	0.0084
278	0.0570	0.0548	0.0580	**0.0573**	**0.0092**	0.0681	0.0630	0.0666	0.0698	0.0044
280	0.0487	0.0412	0.0571	**0.0520**	**0.0103**	0.0635	0.0681	0.0719	0.0707	0.0074
282	0.0668	0.0601	0.0552	**0.0591**	**0.0053**	0.0750	0.0595	0.0705	0.0681	0.0081
284	0.0558	0.0596	0.0626	**0.0556**	**0.0039**	0.0808	0.0798	0.0608	0.0727	0.0075
286	0.0579	0.0663	0.0633	**0.0657**	**0.0055**	0.0784	0.0735	0.0820	0.0744	0.0055
288	0.0637	0.0615	0.0571	**0.0605**	**0.0058**	0.0713	0.0670	0.0691	0.0682	0.0034
290	0.0332	0.0420	0.0511	**0.0601**	**0.0149**	0.0790	0.0446	0.0660	0.0731	0.0116
292	0.0615	0.0745	0.0586	**0.0639**	**0.0090**	0.0659	0.0628	0.0584	0.0675	0.0041
294	0.0544	0.0582	0.0534	**0.0518**	**0.0071**	0.0764	0.0613	0.0799	0.0677	0.0104
296	0.0520	0.0489	0.0554	**0.0516**	**0.0044**	0.0647	0.0773	0.0757	0.0710	0.0066
298	0.0623	0.0552	0.0541	**0.0522**	**0.0062**	0.0739	0.0708	0.0725	0.0728	0.0019

C. GP-mediated lipid mixing after incubation

No Ca2+

	Incubated GPΔmuc					Incubated GP_CL				
time	rep1	rep2	rep3	mean	error bars	rep1	rep2	rep3	mean	error bars
0	0.0000	0.0000	0.0000	**0.0000**	**0.0000**	0.0005	0.0004	0.001	0.0000	0.0006
8	0.0523	0.0524	0.0524	**0.0524**	**0.0001**	0.014	0.0123	0.0101	0.0113	0.0022
17	0.0661	0.0662	0.0661	**0.0662**	**0.0001**	0.0137	0.0155	0.0133	0.0143	0.0022
25	0.0887	0.0888	0.0887	**0.0887**	**0.0001**	0.0124	0.0167	0.0131	0.0134	0.0023
33	0.0984	0.0985	0.0989	**0.0979**	**0.0009**	0.0179	0.0162	0.0231	0.0224	0.0028
42	0.1083	0.1081	0.1092	**0.1088**	**0.0009**	0.0228	0.0231	0.0239	0.0244	0.0033
50	0.1216	0.1238	0.1214	**0.1225**	**0.0010**	0.024	0.0204	0.0238	0.0239	0.0034
58	0.1303	0.1310	0.1271	**0.1288**	**0.0014**	0.0222	0.0269	0.0289	0.0303	0.0037
67	0.1471	0.1442	0.1424	**0.1419**	**0.0044**	0.0367	0.0367	0.0408	0.0356	0.0042
75	0.1394	0.1396	0.1445	**0.1405**	**0.0046**	0.0416	0.0364	0.0311	0.0345	0.0042
83	0.1395	0.1400	0.1875	**0.1478**	**0.0128**	0.0399	0.0431	0.0426	0.0376	0.0044
92	0.1880	0.1799	0.1778	**0.1623**	**0.0162**	0.0423	0.0349	0.0357	0.0418	0.0047
100	0.1687	0.1373	0.1657	**0.1692**	**0.0184**	0.0376	0.0421	0.0365	0.0395	0.0051

0.5 mM Ca2+

	Incubated GPΔmuc					Incubated GP_CL				
time	rep1	rep2	rep3	mean	error bars	rep1	rep2	rep3	mean	error bars
0	0.0000	0.0000	0.0000	**0.0000**	**0.0000**	0.0001	0.0004	-0.0004	0.0000	0.0006
8	0.0905	0.0877	0.0793	**0.0856**	**0.0049**	0.0216	0.0233	0.0199	0.0239	0.0022
17	0.1167	0.1199	0.1128	**0.1164**	**0.0053**	0.0295	0.0261	0.0249	0.0284	0.0022
25	0.1339	0.1399	0.1318	**0.1380**	**0.0059**	0.0369	0.0378	0.0362	0.0368	0.0023
33	0.1522	0.1652	0.1544	**0.1566**	**0.0065**	0.0438	0.0444	0.0442	0.0423	0.0028
42	0.1863	0.1798	0.1726	**0.1832**	**0.0069**	0.0461	0.0477	0.0450	0.0468	0.0033
50	0.1844	0.1838	0.1894	**0.1879**	**0.0073**	0.0579	0.0502	0.0487	0.0527	0.0034
58	0.1957	0.2079	0.1832	**0.1998**	**0.0081**	0.0522	0.0519	0.0497	0.0545	0.0037
67	0.2247	0.2171	0.2294	**0.2215**	**0.0082**	0.0548	0.0673	0.0604	0.0601	0.0042
75	0.2346	0.2480	0.2305	**0.2340**	**0.0085**	0.0597	0.0680	0.0595	0.0635	0.0042
83	0.2404	0.2472	0.2521	**0.2431**	**0.0086**	0.0703	0.0622	0.0601	0.0635	0.0044
92	0.2428	0.2301	0.2518	**0.2535**	**0.0088**	0.0739	0.0650	0.0677	0.0676	0.0047
100	0.2812	0.2929	0.2765	**0.2789**	**0.0092**	0.0740	0.0718	0.0670	0.0703	0.0051

S3 FIG Data. Infectivity in the presence of enclomiphene, tetrandrine, and verapimil. Data are presented as the mean of 3-6 replicates, with errorbars reflecting the standard error.

Tetrandrine

**concentration**	rep1	rep2	rep3	rep4	rep5	rep6	**mean**	**error**
0.0000E+00	100.0000	100.0000	100.0000	100.0000	100.0000	100.0000	**100.0000**	**0.0000**
2.4691E-08	102.0994	105.1765	78.9063	74.8148	84.4720	60.4790	**84.3247**	**6.9262**
7.4074E-08	105.5249	102.8235	62.5000	63.7037	70.8075	52.6946	**76.3424**	**9.1179**
2.2222E-07	81.5470	105.6471	46.7969	52.5185	50.5590	35.9281	**62.1661**	**10.6750**
6.6667E-07	27.7348	57.8824	14.5313	17.5556	15.2174	25.1497	**26.3452**	**6.6798**
2.0000E-06	3.9779	7.2941	1.0156	1.1852	0.5590	4.1916	**3.0372**	**1.0645**
6.0000E-06	0.5414	1.1765	0.5938	0.4667	0.5466	1.6168	**0.8236**	**0.1906**

Verapamil

**concentration**	rep1	rep2	rep3	rep4	rep5	**mean**	**error**
0	100	100	100	100	100	**100.0000**	**0.0000**
1.372E-07	112.707182	94.8235294	112.5	108.888889	91.3043478	**104.0448**	**4.5681**
4.115E-07	130.38674	107.529412	110.15625	105.925926	92.5465839	**109.3090**	**6.0851**
1.235E-06	132.596685	115.764706	109.375	105.185185	88.8198758	**110.3483**	**7.1253**
3.704E-06	51.6022099	51.5294118	60.390625	50.5185185	40.4347826	**50.8951**	**3.1672**
1.111E-05	10.718232	28	15	13.1851852	11.8012422	**15.7409**	**3.1473**
3.333E-05	1.43646409	13.6470588	4.140625	3.40740741	1.80124224	**4.8866**	**2.2463**
1.000E-04	1.04972376	4.47058824	4.140625	1.92592593	2.73291926	**2.8640**	**0.6481**

Verapamil

**concentration**	rep1	rep2	rep3	**mean**	**error**
0	100	100	100	**100.0000**	**0.0000**
1.372E-08	81.7679558	130.823529	74.5856354	**95.7257**	**13.6879**
4.115E-08	76.6850829	123.529412	60.2209945	**86.8118**	**14.6895**
1.235E-07	67.8453039	94.1176471	55.801105	**72.5880**	**8.7625**
3.704E-07	45.0828729	85.6470588	35.9116022	**55.5472**	**11.8366**
1.111E-06	8.06629834	23.5294118	7.18232044	**12.9260**	**4.1114**
3.333E-06	0.91712707	7.29411765	2.70718232	**3.6395**	**1.4709**
1.000E-05	0.34254144	1.88235294	1.60220995	**1.2757**	**0.3668**
3.000E-05	0.27624309	0.20988235	0.99447514	**0.4935**	**0.1946**

S6 FIG Data. Histogram of inter-fluorophore distances from MD simulation.

Distance (A)	Count/Total	Distance (A)	Count/Total
1	0	16	0.0047
1.5	0	16.5	0.0237
2	0	17	0.0095
2.5	0	17.5	0.0284
3	0	18	0.0284
3.5	0	18.5	0
4	0	19	0.0142
4.5	0	19.5	0.0095
5	0	20	0
5.5	0	20.5	0
6	0	21	0.0047
6.5	0	21.5	0.0142
7	0	22	0.019
7.5	0	22.5	0
8	0	23	0.0047
8.5	0	23.5	0.0142
9	0	24	0.0142
9.5	0	24.5	0.0047
10	0	25	0.0047
10.5	0	25.5	0.0095
11	0	26	0.0142
11.5	0.0332	26.5	0.0047
12	0.1327	27	0
12.5	0.2559	27.5	0
13	0.1232	28	0
13.5	0.0664	28.5	0.0047
14	0.0758	29	0.0095
14.5	0.0427	29.5	0.0047
15	0.019	30	0
15.5	0.0047		

S7 FIG Data. FRET histograms and FRET state occupancy data. Columns are colored as in S7 Fig.

GP0

FRET histograms

bins	pH7	error	pH4.5	error
-0.1	0.0003	0.0002	0.0004	0.0004
-0.06	0.0006	0.0002	0.0002	0.0002
-0.02	0.0012	0.0005	0.0025	0.0005
0.02	0.0024	0.0009	0.0051	0.0005
0.06	0.008	0.0013	0.0062	0.0005
0.1	0.013	0.0036	0.0145	0.0026
0.14	0.0196	0.0049	0.0176	0.0014
0.18	0.021	0.0044	0.0174	0.0023
0.22	0.0318	0.0046	0.019	0.004
0.26	0.029	0.0033	0.0207	0.005
0.3	0.03	0.0013	0.017	0.0037
0.34	0.0287	0.004	0.0147	0.0022
0.38	0.0276	0.0026	0.0143	0.0007
0.42	0.0259	0.003	0.0107	0.0019
0.46	0.0269	0.0047	0.0098	0.0011
0.5	0.0305	0.0036	0.0143	0.0018
0.54	0.0339	0.0095	0.0176	0.0019
0.58	0.0301	0.0035	0.0132	0.0008
0.62	0.0263	0.0052	0.0176	0.0022
0.66	0.0298	0.0035	0.019	0.0014
0.7	0.0261	0.0033	0.0183	0.0017
0.74	0.0282	0.0032	0.0199	0.0015
0.78	0.0285	0.0042	0.0332	0.0046
0.82	0.0343	0.0058	0.035	0.0029
0.86	0.0444	0.0064	0.0446	0.0043
0.9	0.0572	0.0068	0.0622	0.0088
0.94	0.0819	0.0021	0.0964	0.0176
0.98	0.1238	0.0105	0.1575	0.0127
1.02	0.0943	0.0108	0.1778	0.0131
1.06	0.0335	0.0046	0.0616	0.0036
1.1	0.0122	0.0006	0.0207	0.0005
1.14	0.0062	0.0017	0.01	0.0022
1.18	0.0129	0.0062	0.0107	0.0044

S8 FIG DATA. sNPC1-C ELISA data. Presented in S8 Fig as the average of 3 replicates (rep1-3), with error bars derived from the standard deviation.

**pH5**					
**sNPC1-C (mM)**	rep1	rep2	rep3	**average**	**error**
0	0	0	0	**0.0000**	**0.0000**
0.0022	0.6363	0.6361	0.6019	**0.6248**	**0.0198**
0.0044	1.0765	1.0996	1.1106	**1.0956**	**0.0174**
0.0088	1.1364	1.1165	1.1149	**1.1226**	**0.0120**
0.0175	1.3357	1.2849	1.2758	**1.2988**	**0.0323**
0.035	1.4881	1.4228	1.2273	**1.3794**	**0.1357**
0.07	1.5455	1.6069	1.4129	**1.5218**	**0.0992**

**pH7**					
**sNPC1-C (mM**	rep1	rep2	rep3	**average**	**error**
0	0	0	0	**0.0000**	**0.0000**
0.0022	0.0259	0.025	0.0387	**0.0299**	**0.0077**
0.0044	0.3496	0.3495	0.3575	**0.3522**	**0.0046**
0.0088	0.4101	0.4099	0.4288	**0.4163**	**0.0109**
0.0175	0.5393	0.5546	0.5312	**0.5417**	**0.0119**
0.035	0.6782	0.6761	0.677	**0.6771**	**0.0011**
0.07	0.846	0.8508	0.8487	**0.8485**	**0.0024**

**pH6**					
**sNPC1-C (mM)**	rep1	rep2	rep3	**average**	**error**
0	0	0	0	**0.0000**	**0.0000**
0.0022	0.502	0.5298	0.5427	**0.5248**	**0.0208**
0.0044	0.9048	0.9035	0.9064	**0.9049**	**0.0015**
0.0088	1.0264	1.0503	1.0145	**1.0304**	**0.0182**
0.0175	1.0377	1.0258	1.0268	**1.0301**	**0.0066**
0.035	1.1324	1.1333	1.0314	**1.0990**	**0.0586**
0.07	1.0792	1.0779	1.0857	**1.0809**	**0.0042**

**pH8**					
**sNPC1-C (mM**	rep1	rep2	rep3	**average**	**error**
0	0	0	0	**0.0000**	**0.0000**
0.0022	0.0251	0.0114	0.0294	**0.0220**	**0.0094**
0.0044	0.2388	0.2611	0.2465	**0.2488**	**0.0113**
0.0088	0.3113	0.272	0.3037	**0.2957**	**0.0208**
0.0175	0.3985	0.3387	0.3544	**0.3639**	**0.0310**
0.035	0.3824	0.3869	0.3891	**0.3861**	**0.0034**
0.07	0.5063	0.5255	0.4679	**0.4999**	**0.0293**

S9 FIG Data. FRET histograms and FRET state occupancy data. Columns are colored as in S9 Fig. Data taken in the absence of EDTA are repeated from Fig 3.

GPΔmuc + 0.5 mM Ca2+ + 10 mM EDTA

FRET histograms

bins	pH7	error	pH4.5	error
-0.1	0.0014	0.0007	0.0013	0.0009
-0.06	0.0007	0.0002	0.0011	0.0006
-0.02	0.0017	0.0007	0.003	0.0008
0.02	0.0036	0.0002	0.0093	0.0032
0.06	0.0088	0.001	0.0181	0.0044
0.1	0.0115	0.0026	0.0343	0.0049
0.14	0.0179	0.0011	0.0517	0.0045
0.18	0.0231	0.0024	0.0577	0.0047
0.22	0.0251	0.0024	0.0579	0.0038
0.26	0.0309	0.003	0.0555	0.0002
0.3	0.0263	0.0025	0.0496	0.0023
0.34	0.0283	0.003	0.0444	0.0019
0.38	0.0256	0.0009	0.0385	0.0009
0.42	0.0266	0.003	0.0369	0.0004
0.46	0.0245	0.002	0.0313	0.0026
0.5	0.0258	0.0024	0.0276	0.0012
0.54	0.028	0.0008	0.0239	0.0026
0.58	0.026	0.0002	0.0203	0.0033
0.62	0.0258	0.001	0.0209	0.0025
0.66	0.026	0.0016	0.0185	0.0034
0.7	0.0255	0.0005	0.0196	0.0015
0.74	0.0293	0.002	0.021	0.0017
0.78	0.0308	0.0028	0.0253	0.0032
0.82	0.0321	0.0052	0.0323	0.0018
0.86	0.0349	0.0058	0.0387	0.0001
0.9	0.0496	0.0115	0.0471	0.0035
0.94	0.0725	0.0126	0.0596	0.007
0.98	0.1227	0.0098	0.0689	0.0088
1.02	0.1242	0.0131	0.0492	0.0052
1.06	0.0539	0.0042	0.0194	0.0031
1.1	0.0213	0.0021	0.0092	0.0024
1.14	0.0078	0.0003	0.0045	0.0019
1.18	0.0077	0.0011	0.0033	0.0015

FRET state occupancies: GPΔmuc

		High FRET	error	Int. FRET	error	Low FRET	error
**pH7**	No Ca2+	0.6821	0.021	0.2047	0.0139	0.1132	0.0137
**pH7**	0.5 mM Ca2+	0.5031	0.0276	0.2361	0.0176	0.2609	0.0188
**pH7**	10 mM EDTA	0.7058	0.02	0.1834	0.02	0.1108	0.02
**pH4.5**	No Ca2+	0.3912	0.0341	0.2575	0.0185	0.3513	0.0267
**pH4.5**	0.5 mM Ca2+	0.2901	0.0288	0.2709	0.018	0.439	0.0239
**pH4.5**	10 mM EDTA	0.3838	0.02	0.2387	0.02	0.3775	0.02

GP_CL + 0.5 mM Ca2+ + 10 mM EDTA

FRET histograms

bins	pH7	error	pH4.5	error
-0.1	0.0021	0.0017	0.0014	0.0004
-0.06	0.0014	0.0003	0.0006	0.0001
-0.02	0.0021	0.0003	0.0019	0.001
0.02	0.0042	0.0009	0.0078	0.0011
0.06	0.009	0.0011	0.0138	0.0016
0.1	0.0151	0.0028	0.0279	0.0047
0.14	0.0269	0.0042	0.0357	0.0047
0.18	0.0299	0.0059	0.0365	0.0036
0.22	0.0349	0.0075	0.0377	0.0029
0.26	0.0372	0.0113	0.0444	0.0067
0.3	0.033	0.0106	0.0425	0.0059
0.34	0.0379	0.0131	0.0439	0.0049
0.38	0.0363	0.0088	0.0418	0.0038
0.42	0.0318	0.0088	0.0444	0.0059
0.46	0.0346	0.0074	0.0436	0.0041
0.5	0.0264	0.0051	0.0352	0.0015
0.54	0.0287	0.0052	0.036	0.0018
0.58	0.0285	0.0037	0.0377	0.0035
0.62	0.0254	0.0051	0.0333	0.0025
0.66	0.0297	0.0054	0.0365	0.0023
0.7	0.0302	0.0037	0.0334	0.0064
0.74	0.0325	0.005	0.033	0.0027
0.78	0.0325	0.0047	0.0346	0.0073
0.82	0.0313	0.0023	0.0366	0.0044
0.86	0.0417	0.0038	0.0393	0.0041
0.9	0.0485	0.0074	0.0428	0.0073
0.94	0.0676	0.0175	0.0491	0.0098
0.98	0.0957	0.022	0.0564	0.0129
1.02	0.0855	0.0123	0.0366	0.008
1.06	0.0292	0.0031	0.0178	0.004
1.1	0.0132	0.0017	0.0111	0.0038
1.14	0.0068	0.0037	0.0035	0.0013
1.18	0.0099	0.0044	0.0033	0.0008

FRET state occupancies: GP_CL

		High FRET	error	Int. FRET	error	Low FRET	error
**pH7**	No Ca2+	0.6265	0.021	0.2181	0.0139	0.1554	0.0137
**pH7**	0.5 mM Ca2+	0.3631	0.0276	0.2361	0.0176	0.4009	0.0188
**pH7**	10 mM EDTA	0.5656	0.02	0.1761	0.02	0.1973	0.02
**pH4.5**	No Ca2+	0.2959	0.0335	0.2759	0.0201	0.4282	0.0252
**pH4.5**	0.5 mM Ca2+	0.1739	0.0282	0.217	0.0165	0.6091	0.0238
**pH4.5**	10 mM EDTA	0.3723	0.02	0.3554	0.02	0.2723	0.02
