# Supplementary material for: Conformational changes in the Ebola virus membrane fusion machine induced by pH, Ca2+, and receptor binding
Source: PLoS Biol. 2020 Feb 10;18(2):e3000626. doi: 10.1371/journal.pbio.3000626 (PMC7034923; doi:10.1371/journal.pbio.3000626)
Supplement: Attachment — Submitted filename: GP-activation_plos-bio_Response.docx [file pbio.3000626.s012.docx]

**Reviewer #1:**
    
Fusion stage of the entry of Ebola virus is a complex and still poorly understood process. Activation of viral fusogens in virus-carrying endosome depends on a number of factors, including cathepsin-mediated cleavage of envelope glycoprotein GP and Niemann-Pick C1 (NPC1) binding. It has also been suggested to depend on acidification and changes in the calcium concentration associated with maturation of endosomes. This study analyses conformational changes in fusogenic GP2 domain of GP using an elegant approach they have developed in their earlier work on flu hemagglutinin. The loss of initial conformation of GP2 is detected at a single molecule level by a single-molecule Förster resonance energy transfer (smFRET) imaging assay as a decrease in resonance energy transfer reflecting an increase in the distance between two fluorophores placed at positions that are close to each other in the initial conformation. Based on the smFRET data and a bulk assay that detects lipid mixing between DiO-labeled GP-pseudoviruses and liposomes as dequenching of DiO, the authors conclude that low pH and Ca^2+^ dependent reversible shifts of GP2 conformations to intermediate state(s) prime the protein for NPC1 binding dependent irreversible conformational change associated with fusion. While the results are mostly convincing, definitely novel and significant, and will be of importance for researchers working on entry stages of Ebola virus, and on fusion mediated by other protein fusogens, I do have a number of questions/suggestions. While the smFRET part of the work is very well designed, my questions are mostly related to a virus-liposome lipid mixing assay. I consider two of the major comments below as essential to support the current conclusions. 

**We are grateful to the reviewer for his/her supportive comments. We have diligently tried to address those comments below.**

Major comments:

1) The DiO dequenching data are presented as the fold increase over initial fluorescence (F/F0). Liposome-based fluorescence dequenching fusion assays conventionally require application of a detergent at the end of the experiment to normalize the extent of the dequenching to the maximum dequenching with infinite dilution of the probe. Without it, we do not know the extent of DiO quenching in the viral particles prior to fusion and cannot evaluate the efficiency of the lipid mixing. Also, it appears that the extent of DiO dequenching is very very low (the largest increase is ~1.6 fold) comparing with what they had in the Cell paper for HA (Ref 35), where DiO fluorescence went from ~0 to 2000 in 150 s. Does it mean that GP2-mediated virus/liposome lipid mixing is much much less efficient than for flu? If this is the case, it is essential to verify that dequenching cannot be explained by fusion-unrelated lipid probe transfer. 

**The reviewer makes an excellent point, which we were remiss in not including in the initial draft of the manuscript (although we had performed this control). In the revised manuscript the fluorescence observed upon lipid mixing is now presented as a percentage of the maximally dequenched fluorescence observed in the presence of 1% triton.**

**The addition of 1% triton leads to an approximately 10-fold increase in DiO fluorescence, which is roughly a 5-fold greater fluorescence dequenching than occurs due to pseudovirus-liposome lipid mixing. However, we feel this cannot be interpreted as indication that GP-mediated pseudovirus-liposome lipid mixing is only 20% efficient. For each pseudovirus-liposome fusion event DiO is only diluted by approximately 2.8-fold (150nm virion fusing to a 200nm liposome), which may not lead to the same level of dequenching that occurs upon “infinite dilution” due to addition of detergent. Nonetheless, knowing the level of dequenching that occurs upon addition of detergent provides a standard by which to compare data from different experiments. Therefore, as the reviewer points out, this is worth including. Finally, 20% dequenching observed here is in line with previous measurements of lipid mixing mediated by other viral glycoproteins (20-50% for influenza HA [Gui and Lee, Methods Mol Biol 2018; Wessels et al. Biophys J 2007], and 25% for Lassa GP [Thomas et al., PLoS One 2012]).**

**In our previous publication, HA-mediated lipid mixing data was presented as background-subtracted raw fluorescence intensity. Since raw fluorescence intensity values will depend on numerous parameters, including excitation intensity, slit widths in the fluorimeter monochromators, and sample concentration, we felt that a better presentation was to report fold increase in fluorescence (which has now been replaced by the percentage of dequenching seen with 1% triton). This will make the signal more easily comparable to future experiments. As the reviewer correctly points out, our HA-mediated lipid mixing experiment generated an increase in fluorescence intensity of about 2000 intensity units over 150 seconds. Expressed as fold-increase over background, this equates to just over a 2-fold increase. Thus, GP-mediated lipid mixing is modestly less efficient that HA. Notably, a physiological concentration of Ca^2+^ (0.1-0.5 mM) was essential to achieving this level of dequenching.**

2) The dependence of the GP2 restructuring on Ca is one of the most intriguing features of the process. The effects of TPC inhibitors on Ebola virus infection and lipid mixing inhibition by 1 mM and higher concentrations of Ca on the lipid mixing suggests that fusogenic restructuring of GP2 depends on decrease rather than increase in Ca. In this scenario millimolar concentrations of Ca in the extracellular environment act as one of the blocks for premature restructuring and inactivation of the protein. Have you checked the reversibility of low pH and Ca effects in the context of virions and functional experiments as an inactivation? Do you observe an irreversible or reversible loss of fusogenic activity of the virus for higher concentrations of Ca (S2B Fig.), especially, prior to NPC1? 

**This is an interesting and thoughtful comment, which we had not fully considered. At present, we can say that 0.1-0.5mM Ca^2+^ yields optimal lipid mixing in our assay. This result correlates with promotion of conformational changes in GP, which likely relate to its mechanism of fusion. Upon further consideration, in part prompted by the comments of this reviewer, we acknowledge that the effect of elevated Ca^2+^ is presently unclear. Three possible explanations for the loss of lipid mixing in the presence of elevated Ca^2+^ are apparent: (1) elevated Ca^2+^ leads to inactivation by premature triggering of GP; (2) elevated Ca^2+^ inhibits GP conformational changes necessary for fusion; or, (3) elevated Ca^2+^ leads to transition to an off-pathway GP conformation that is not competent to mediate fusion. At present, based on the results shown in this manuscript, we cannot distinguish between these possibilities. In the revised manuscript we have acknowledged all 3 possibilities and removed any claims as to a preferred explanation.**

**The reviewer also makes the excellent suggestion to consider the ability of GP to mediate lipid mixing after transient exposure to acidic pH and Ca^2+^. We therefore incubated pseudovirions with uncleaved GPΔmuc for 10 minutes at pH 4.5, followed by proteolytic removal of the glycan cap. Under these circumstances GP mediated efficient lipid mixing with liposomes coated with sNPC1-C. However, if GPΔmuc was cleaved (generating GP^CL^) prior to incubation at pH 4.5, a substantial loss of lipid mixing was observed. The same essential result was obtained in the presence of 0.5mM Ca^2+^. This result nicely supports our conclusion that the presence of the glycan cap prevents conversion to an irreversible GP conformation, which is no longer capable of mediating lipid mixing. These data are now presented in S2D Fig.**

**We are already initiating future studies aimed at better understanding the mechanism by which Ca^2+^ affects GP conformation. These studies will include verification of the putative Ca^2+^ binding sites, and determination of the effect of elevated Ca^2+^ on GP conformation. Specifically, we will seek to determine whether elevated Ca^2+^ is leading to premature triggering of GP, transition to an off-pathway conformation, or inhibition of triggering. At this stage we prefer to hold off on further investigations of the effects of elevated Ca^2+^ on lipid mixing until we have a better understanding of the effects on GP conformation, and will thus be more able to interpret the results. As already noted above, we have removed from the manuscript any preferred explanation of the effects of elevated Ca^2+^ and leave it as an open question for future studies to distinguish between the above noted possibilities. We appreciate that the reviewer finds this topic of particular importance and hope he/she will find our future work of similar interest.**

Minor comments:

1) I am confused by what appears to be a discrepancy between the main text and the legend to S1 Fig. The text states "Pseudovirions labeled with DiO maintained approximately 75% infectivity as compared to unlabeled pseudovirions" and the legend to the S1 Fig. states "Infectivity of GFP-expressing VSV particles pseudotyped with GP or GP*, or labeled with the lipophilic fluorophore DiD, which is spectrally distinct from GFP and chemically similar to the DiO fluorophore used in the hemifusion assay.". Have the authors examined the infectivity for virions labeled with DiO (as in functional experiments) or with DiD?

**The reviewer correctly points out that this was not clearly written. The in-vitro lipid mixing assay was done with DiO. But DiO emission overlaps with GFP emission used to report on infection. So the infectivity assays were performed with virus labeled with DiD. Both DiO and DiD are carbocyanine fluorophores, with similar chemical structures, including identical fatty acid moieties, which target to the viral membrane. So we are confident that the infectivity of DiD-labeled virus is representative of DiO-labeled virus used in the in-vitro lipid mixing assay. We have clarified this in the Fig. S1 legend, and the Materials and Methods.**

2) I suggest the authors to refer to their DiO dequenching assay as lipid mixing assay rather than hemifusion assay. In the absence of other assays, we cannot distinguish hemifusion from complete fusion here because complete fusion would also result in lipid mixing and DiO dequenching.

**Agreed. The reviewer is absolutely correct. We have made this change throughout the manuscript.**

3) Line 411: "virus and liposomes were co-incubated at room temperature for 5 minutes to enable virus-liposome binding at pH 7.5." What binding mechanism you have in mind?

**Indeed this statement in the manuscript was not clear. Only in the case of pseudovirions containing GP^CL^ and liposomes containing sNPC1-C to we envision virus-liposome binding by way of GP^CL^-NPC1 interaction. With GPΔmuc and/or liposomes lacking sNPC1-C, we envision no binding between pseudovirions and liposomes. This text has now been clarified.**

4) I am unclear on whether the authors used 1-oleoyl-2-palmitoyl-snglycero-3-phosphocholine as stated in line 403 (Avanti # 850457) or POPC (line 403) (1-palmitoyl-2-oleoyl-glycero-3-phosphocholine) (Avanti #850475).

**Thanks to the reviewer for catching this error. We used 1-palmitoyl-2-oleoyl-glycero-3-phosphocholine (POPC). This has been corrected in the text.**

**Reviewer #2:** 
    
    Das, D. K. et al present a thorough study of the role of pH, Ca2+, and target receptor binding for the Ebola virus glycoprotein spike. These authors use a smFRET approach to estimate the conformational dynamics of a domain in the spike under differing ligated conditions for the aforementioned species. The results demonstrate that these ions and the sNPC1 receptor fragment are required to yield a low FRET state, a conditions the authors speculate is the post-fusion state based upon atomic structures of the two states. This elegant and mechanistically insightful study should certainly be published after addressing this reviewer's concerns.
    
Major points:
    
1) This reviewer envisions the sequence of events for GP encounter between the relevant players discussed in this study to occur in this order: Lower pH, lower Ca2+, additional pH reduction, and then a final encounter between the NPC1 luminal domain in a late endosome/lysosome (similar to the mechanistic model cartoon). The author's experimental setup, however, appears to create a steady state with the addition of sNPC1 to GP (and variants) and then observe the change in smFRET relative to pH modulation or steady-state Ca2+ addition. Have the authors attempted to perform a time series smFRET measurement by perfusing in sNPC1 under acidic conditions and 0.5 mM Ca2+? This would help to rectify the proposed linear cartoon model, suggesting that low pH and Ca2+ are required a priori to initiate NPC1 receptor engagement. If this experiment were performed and the resulting occupancy distributions were nearly the same, this would suggest that the mechanism does not require a specific order of events, simply all of these ingredients are required.

**The reviewer raises an outstanding point, which we would love to be able to address. Indeed, we are studying GP conformation under quasi-equilibrium (steady-state) conditions, and determining how different variables affect that equilibrium. At present, we are limited to such steady-state measurements. In the future we hope to be able to conduct pre-steady state experiments where variables such as the presence of NPC1 are presented to GP during imaging, such that we are able to visualize the trajectory in real time. At present, the fluorophore lifetime does not permit such measurements. As shown in Figures 3 and 4, fluorescence only lasts for about 3 seconds before photobleaching. In contrast, Figure 1 shows that lipid mixing plateaus after about 200 seconds. Therefore we do not currently have a sufficient window of observation in our smFRET experiments to visualize the pre-steady state trajectory of GP conformation that promotes lipid mixing. This remains a goal for future study.**

**One point is worth clarifying: during our smFRET experiments all components (pH, Ca^2+^, and NPC1) are presented at the same time to GP. We then evaluate the effect of that combination on GP conformation. The proposed sequence of events depicted in Figure 5 stems from the fact that pH and Ca^2+^ alone promote transition to the reversible low-FRET state. Only after glycan cap cleavage and NPC1 binding do we achieve the irreversible low-FRET state. Our proposition is that the reversible state is achieved prior to the irreversible state.**

2) The data in Figure 1 should be fit to a model and quantitative estimates should be extracted to report relative changes in rates of fusion compared with a standard condition (e.g. sNPC1 + Ca2+).

**This is a great suggestion. The dequenching curves in Figure 1 have now been fit to exponential functions (y = A(1-exp(-kt)), and the fits are specified in the figure legend. The fits were not well determined in the cases lacking Ca^2+^, as the signal did not saturate over the observation window, and thus fits are not indicated for these data. Also, the dequenching data recorded in the absence of NPC1 could not be fit, as they are essentially flat lines.**
    
3) This reviewer wonders if the MD simulation of probes on GP can be used to extract the κ2 value for the dye pair orientation as a function of time to estimate a more accurate, albeit a simulated, explicit distance measurement. Does the distribution of dipolar angles resemble the often abused 2/3 random orientation value for κ2. It is hard to tell from the centroid dots how the dipoles are oriented in the cartoon. If the authors were to extract these values and estimate the mean κ2, would the FRET efficiency convert to distances that resemble those speculated in the paper?

**This is an interesting idea that we have considered, but never followed through on. At the reviewer’s suggestion, we have used the MD simulation of the fluorophore-labeled GP to calculate the orientation parameter of the fluorophores. This required calculation of the transition dipole for both fluorophores, which we carried out in Gaussian. The resulting time-averaged κ^2^ was 0.645 ± 0.320 (mean ± standard deviation). This value is in excellent agreement with randomized fluorophore orientations (κ^2^ = 2/3). Notably, the large standard deviation makes clear that the relative orientation of the fluorophores varies widely over the simulation. However, the simulation provides only a 50ns trajectory, and our experimental time resolution is 40ms per frame. So our experimental measurements are not sensitive to the fluorophore dynamics predicted by the simulation. Therefore we feel that the calculated κ^2^ provides a reasonable estimate, and supports the idea that the observed high-FRET state results from proximal dye positions. Details of the procedure are now provided in the legend to Figure S6 and the Materials and methods section.**

Minor points:
    
1) For all figures displaying the fractional occupancy of a state via smFRET, it should be explained in the methods how the error in occupancy is calculated. Since this parameter is a fractional value and the fit to a multimodal distribution should have only one error, it is not clear how the error is propagated to these plots.
    
2) Quantitative color maps should be includes for all smFRET probability distributions. It is difficult to interpret the gradient changes in the time-lapse measurements.

**Agreed. Colormaps are now included in all relevant figures.**
    
3) Lines 125-126, has anyone measured the luminal concentrations of Ca2+ in late endosomes/lysosomes (the site of entry for EboV)? If so, what is the average molarity? Does this compare well with the 0.5 mM used in the study?

**Like pH, the Ca^2+^ concentration changes during passage through the endocytic pathway. The Ca^2+^ concentration is relatively high in the extracellular space (~mM), but the concentration drops to ≤10µM in early endosomes (Gerasimenko JV et al., Curr Biol 1998) due to the presence of pH dependent Ca^2+^ channels (Saito et al., J Biol Chem 2007). The Ca^2+^ concentration then begins to increase to ~0.4-0.5mM in the late endosome/lysosome (Christensen et al., J Cell Sci  2002). Since Ebola enters through the late endosome, and because the extent of lipid mixing was maximal in the range of 0.1-0.5mM, we performed smFRET experiments 0.5mM Ca^2+^ concentration. In the Discussion section of the revised manuscript we have described this evolution of the Ca^2+^ concentration in the endosomal pathway and made reference to these works.**

4) The authors should add a reference to line 152 to link the audience to what is known about the epitope of the KZ52 antibody.

**We now include reference at this point to Lee et al., Nature 2008, as this work explicitly defines the KZ52 epitope.**

**Reviewer #3:** 
    
The Das et al. manuscript studies Ebola GP fusion protein mediated membrane fusion using pseudovirons. They use a liposome fusion fluorescence dequenching method to reveal membrane hemifusion and single molecule FRET approach to reveal Ebola GP fusion protein conformational changes, as they previously did with HIV and influenza. The authors use the liposome assay to conclude that that low pH, receptor binding, and calcium exposure act in concert to induce the most efficient fusion activity, as is generally believed for Ebola from previous studies. They use the single molecule FRET method to reveal transitions from a prefusion configuration with high FRET to a low FRET state, which is considered to reflect an on-pathway fusogenic state. Correlations among hemifusion lipid mixing signals and single molecule FRET signals under various experimental conditions are used to support the interpretation of these FRET states as fusion-related conformations. The authors map the populations of events with the different FRET signals under several conditions including the removal of the glycan cap, receptor presence as well as pH and calcium exposure. Controls with different divalent ions, inactivating antibodies and drugs used to inhibit Ebola are included, strengthening the arguments. The most novel finding in the work is identification of reversibility in the low pH + calcium induced conformational change (transition to low FRET) that is made permanent in the presence of the cognate receptor. Finally, there is a general discussion of a model for Ebola membrane fusion in the context of existing models for influenza and HIV. 
    
The manuscript is clear and the experiments are expertly performed. The work represents a high level of technical sophistication. The topic of Ebola membrane fusion is important and interesting to a wide array of scientists. This approach to mechanistic studies of virus phenomena is exciting and likely to be enduring in the field. The model of several synergistic interactions regulating virus fusion and entry into cells is innovative. Nevertheless, there are a few concerns with the work that are essential to address.
    
**We are grateful to the reviewer for his/her supportive comments. We have addressed each point specifically below.**
    
    1 It would be useful to confirm the observation of reversibility of the transition from prefusion configuration to fusogentic state (in the absence of receptor) in the liposome assay, if possible. Perhaps experiments with various sequences of pre-exposure to changes in pH/calcium with or without receptor present, and then assay for hemifusion competence in the liposome assay would be useful. Such experiments could be highlighted as supporting the existence of this reversible state that is on pathway supporting membrane fusion.

**As also noted above in the response to Reviewer 1, this is an excellent suggestion, which has now been addressed in the revised manuscript. We incubated pseudovirions with uncleaved GPΔmuc for 10 minutes at pH 4.5, followed by proteolytic removal of the glycan cap. Under these circumstances GP mediated efficient lipid mixing with liposomes coated with sNPC1-C. However, if GPΔmuc was cleaved (generating GP^CL^) prior to incubation at pH 4.5, a substantial loss of lipid mixing was observed. The same essential result was obtained in the presence of 0.5mM Ca^2+^. We ask the reviewer to note that this experiment cannot meaningfully be done with incubation of GP^CL^-containing pseudovirions in the presence of sNPC1-C, as sNPC1-C binding will inhibit attachment to the liposomes during the dequenching assay irrespective of any effect on GP conformation. Nonetheless, these results nicely support our conclusion that the presence of the glycan cap prevents conversion to an irreversible GP conformation, which is no longer capable of mediating lipid mixing. These data are now presented in S2D Fig.**

    2 What is the labeling efficiency of the proteins in the single molecule FRET studies? Maybe this is hard to determine, but it is important for the infectivity and fusion validation work (Figure S1). Lines 151-155 describe measurements of labeled sample infectivity and fusion efficiency as ranging 60%-90% of unlabeled or wild-type. Labeling efficiency could correlate with these effects, suggesting the proteins lacking dyes could account for the active fraction of the population.

**The reviewer raises a fair point. Usually in these circumstances, labeling efficiency is determined by measuring the concentration of protein and the concentration of fluorophore of the labeled sample. The is generally readily accomplished by measuring the absorbance at the relevant wavelengths and estimated extinction coefficients. At best, this procedure provides an estimate of labeling efficiency that is likely correct to within a factor of 2, given the uncertainties associated with absorbance measurements.**

**Unfortunately, in the present case where the sample is not a soluble protein but rather a labeled virion, precise determination of labeling efficiency is not readily accomplished. The standard method described above does not apply since there is no way to convert the absorbance of a virion to the concentration of the envelope glycoprotein specifically. We could in principle determine the number of virions per unit volume using electron microscopy, but we will still not know the number of glycoproteins per virion, which is likely a distribution rather than a specific number.**

**Alternatively, one could envision using the fluorescence intensity of labeled virions imaged with TIRF microscopy (as in our smFRET experiments) to determine the number of fluorophores present on each particle. However, assuming each virion contains at least 10 GP trimers, we would need to resolve at least 30 fluorophores per virion to get an accurate measure labeling. This would provide an absolute measure of labeling efficiency. Unfortunately, we lack the dynamic range to resolve any more than 5 or 6 fluorophores per virion, making this measurement impossible.**

**The best we can say is that the reported efficiencies of copper-free click labeling to the TCO* amino acid are in the range of 90% using 1.5μM fluorophore-tetrazine incubated for 10min at 37C (Sakin et al., Cell Chem Biol 2017; Nikic et al., Nat Prot 2015; Nikic et al., Angew Chem 2014). In our labeling reactions, since we were not limited by the toxicity of the fluorophore, virions containing GP* were labeled using 2mM fluorophore-tetrazine for 10min at 37C. We are therefore confident that our labeling efficiency is at least as high as previously reported numbers; no labeling was observed in the absence of GP*.**

**We are hopeful that the reviewer can appreciate the technical challenges that we face here.**
    
    3 These functionality controls (Figure S1) are also difficult to interpret given that lines 436-440 mention that the virus particles here had 50 fold excess of wild-type GP over the labeling mutant GP*. Does this mean each particle has more wild-type fusion proteins than labeled fusion proteins? Note line 158 mentions pseudovirions were formed with a single GP* protomer among the native distribution of wild-type GP, and line 341 in methods mentions full-length GP0* was translated at approximately 5-10% efficiency as compared to wild-type GP. These statements suggest this is the correct interpretation, which then questions the relevance of the controls – an excess of wild-type, unlabeled GP protein will mask any failure of the FRET reporting GP* in the event that the FRET modifications rendered the GP* or GP*+fluorophore protein not functional for fusion or infection. This information is important to the paper, but as presented is confusing and concerning.

**We thank the reviewer for highlighting that we had not described clearly how the infectivity control measurements were performed. The reviewer is absolutely correct that diluting GP* with wild-type GP would make the functional evaluation of GP* meaningless. Our fusion and infectivity measurements were performed with pseudovirions containing either 100% wild-type GP, 100% GP*, or 100% GP*-Cy3/Cy5 (although, see above). That is, *for the purposes of functional evaluation, GP* and GP*-Cy3/Cy5 were not diluted with wild-type GP.* In the revised manuscript, we have made this clearer in the Materials and methods and in the legend to S1 Figure.**

    4 The authors also observe a FRET event near 0.52 that is not claimed to be directly related to the pre-activation or fusogenic states.  This state does not change under most conditions: line 170: "intermediate-FRET state (0.52 ± 0.09), which did not respond to changes in pH" or in figure 3 the intermediate-FRET state does not change with calcium either.  That FRET 0.52 state is similar in all conditions and all mutants in figure 3 (20-30% populations). The authors only comment once to address possible ways to interpret it, saying near line 254 – "The intermediate-FRET state may reflect a more localized rearrangement of the fusion loop that precedes its large-scale displacement". Is it reasonable that this FRET 0.52 state is related to anything functional if this state does not change with pH or calcium? The authors should try to understand this state more completely.

**The existence of the intermediate-FRET state has been one of the more myserious aspects of these data. Indeed, we have struggled to know how to interpret it because of its short lifetime, which is not responsive to the variables that we have considered. Our intention was only to make a simple suggestion as to what this state might represent in structural terms. But as the reviewer correctly implies, any such statement is purely speculation. We agree with the reviewer that we cannot rule out the possibility that the intermediate FRET state has no functional relevance. In fact, given the lack of response to the biological variables considered here, this might be the simplest explanation. We now expand upon this discussion, and include the notion that the state may not be functionally relevant.**

**Reviewer #4:** 
    
The authors have created a construct of the Ebola GP protein that allows Cy3 to be attached to one site, and Cy5 to another. If there are changes in the distance between these two dyes, their dipolar orientation, the dielectric constant of the medium between them, or other changes to the electronic configuration of either dye, there will be a change in the FRET signal. They measure the both donor and acceptor fluorescence from single molecules as a function of time after changing pH (before they bleach) when the proteins are expressed on pseudoviruses and added to liposomes bearing receptor. When the pH is changed, as modulated by Ca2+, proteolytic cleavage, or addition of soluble receptor, the average FRET can change. These changes are categorized into three states for which gaussian distributions are reported, normalized to these three states. In addition, lipid dequenching is measured as a function of time with these same viron under conditions varied as above, as a measure of fusion or hemifusion.
    
There is much good that this paper contains, with a lot of very good and careful work. I cannot say the same about the writing or even the final conclusion. Instead of leaving the interpretation of data for the Discussion, they intersperse it into the Results, making a very complicated first paragraph with not much interpretation of results, or a summary of the main experimental findings. They can certainly propose a model, but first they need to discuss their results. They would be well advised to move all interpretation into the Discussion.

**We thank the reviewer for his/her careful consideration of our manuscript. We have worked diligently to improve the writing of the manuscript, including moving interpretations to the Discussion section.**

End of Abstract and lines 270-276: It is not clear that the data here suggests protection of internalized Ebola in early entry until a "more permissive environment" is reached – presumably maturing of early endosomes. The one paper I found (Albrecht et al. Cell Calcium 57:263-274, 2015) with good endosomal targeting and genetic-encoded sensor detection of endosomal Ca++ suggests the opposite, since endosomal Ca++ starts below 5 µM and falls even lower with maturation. Thus lines 270 – 276 seem speculative as there are no experiments in this paper showing the actual Ca++ in the endosome is actually protective, and is represented within the ranges of the entire biphasic response to Ca++. The cited (Fan et al.) paper on endosomal calcium and SERM and Ebola indicates that chelating Ca blocks fusion, but even the SERM which also block Ebola only double Ca++ release, but they have to somehow bring endosomal Ca++ from 2 µM to over 1 mM??? So the biphasic nature of the Ca response is not sufficiently fleshed out in this paper to end the first paragraph of the Discussion with this statement. Furthermore, it seems that cholesterol is a better candidate for SERM action.

**There appears to be some disagreement in the literature over the concentration of Ca^2+^ along the endosomal pathway. What is clear is that the extracellular Ca^2+^ concentration is relatively high (1-2mM), and as a result cells internalize Ca^2+^ by endocytosis. The Ca^2+^ concentration then drops to ~10μM in the early endosome due to the action of pH dependent Ca^2+^ channels (Gerasimenko et al., Curr Biol 1998). Estimates of the Ca^2+^ concentration further along the endocytic pathway vary widely (3 to 600μM; Albrecht et al., Cell Calcium 2015; Christensen et al., J Cell Sci 2002; Gerasimenko et al., Curr Biol 1998), and could conceivably be cell-type dependent. Our data indicate that optimal lipid mixing in the context of an in vitro assay occurs in the range of 0.1-0.5mM. We have therefore focused our interpretations on our observables and removed any claims or insinuations as to the endosomal environment in which Ebola fuses. We have also made sure to more completely cite the literature regarding endosomal Ca^2+^.**

Lines 300-302. The last sentence of the paper is very weak. These are not novel ideas and come across as obvious given the involvement of many receptor/fusogen binding reactions that potentiate conformational changes downstream of binding. Surely with such great stuff they can do better. They make it clear in the introduction that far too little is known, and after reading this manuscript I am sure that their hypothesis in line 180-182 is very likely a reasonable and testable hypothesis. This is a solid step forward: "According to this interpretation, acidification promotes a conformational change in GP, which releases the fusion loop from the hydrophobic cleft in the neighboring protomer, perhaps facilitating its interaction with the target membrane." It can be an important concluding model, as fleshed out with their reversibility data vis-à-vis the other intermediates.

**We thank the reviewer for the helpful critique. We have reworked the end of the paper in hopes of ending on a stronger and more focused note.**
    
Line 246. Holistic is going a bit too far for my taste. It is not clear what is the effect of other factors. A new assay is devised for protein conformational change and lipid mixing is measured in permutations of Ca, pH, for two constructs with and without soluble receptor. Not exactly all the interacting factors are integrated into one model. Nor is it particularly surprising that pH causes a conformational change in a viral fusion protein. Moreover, terms appear like 'physiological' which indicate native structures, but in this report the work is done with engineered constructs, usually derivatized both genetically and chemically, and synthetic liposomes.

**Here again, we appreciate the helpful critiques, and acknowledge that “holistic” is too strong of a word. We have toned down these aspects of the discussion.**
    
Other issues:
    
    1.    DiO dequenching is a lipid-mixing assay that occurs in both hemifusion and fusion. For determination of hemifusion or fusion a content mixing assay is also required. Also, lipid dequenching assays for lipid mixing are usually followed by a detergent step to determine maximal dequenching. Then the % dequenching is plotted. I think that gives a better sense of the efficiency or robustness. Here it is relative, so it is not clear what fraction of the viron give lipid mixing at 1.6 F/F0. I could be 1 % or 100%.

**Agreed. As noted above in response to Reviewer 1, we were remiss in not presenting the extent of dequenching in the presence of detergent. All lipid mixing data is now presented as the percentage of dequenching observed upon the addition of 1% triton.**
    
    2.    Lines 129-131- "...cannot rule out...does not proceed..." Double negative, does it mean 'we rule out that it does proceed'? I think this indicates the above problem: hemifusion is the merger of membranes without fusion pore formation, so what is measured here is lipid mixing. It is an aspect of both hemifusion and fusion. It should be called what it is, and it should not be called either hemifusion or fusion, as neither is shown at this point. Usually, the interpretation of the result is left for the discussion section. Changing the standard definition of hemifusion leads to awkward sentences like this one. By calling the observable 'lipid mixing', the authors can use an understandable sentence like:

"At present, we cannot determine what fraction of the lipid mixing corresponds to full fusion or hemifusion."

**Also agreed. Throughout the revised manuscript we now refer to “lipid mixing” instead of hemifusion. In particular, we have revised the problematic sentence highlighted by the reviewer.**
    
    3.    Line 476. There are at least 3 SPARTAN software packages on the web, but the first 40 or so references to SPARTAN software are to another package. I see the correct citation as requested but I do not see on online access to the custom-written scripts. As this is not open sourced but licensed I suggest that the url be provided in parentheses after the SPARTAN (<https://urldefense.proofpoint.com/v2/url?u=https-3A__www.scottcblanchardlab.com_software&d=DwIGaQ&c=WJBj9sUF1mbpVIAf3biu3CPHX4MeRjY_w4DerPlOmhQ&r=_cVppyAm8rjPu_3ASlGWtRyoPe0gAf724D6fnOHq6tQ&m=QidsREJWLPq3ROOI9BL5CFtpNLpfmwQI-7j37d6qMrM&s=6MBpbYSxA7HLtNv7v3zQc_0oyiA-eatj7n_aUMFWuDM&e=>) and the custom written scripts be available to readers.

**The SPARTAN software used here is indeed open source and freely available for download. To eliminate confusion, we now include a URL to the website of Scott Blanchard’s laboratory, from which the relevant SPARTAN can be downloaded.**
    
    4.    Line 752 - There is no such journal 'Sci Rep-uk'. The citation is 'Scientific Reports, 7:41226' However PLOS wants to abbreviate or format it.

**Thank you. This has been fixed.**
    
    5.    Figure 1. 'Efficiencies are different'. It is not clear, since the red signal continues to grow, if lipid mixing efficiency is actually different, one would have to wait. Clearly the rate of lipid mixing is different, but that is what you get when you compare two time points, not the extent of lipid mixing (I assume you mean extent by 'efficiency'.

**Indeed “efficiency” is the wrong word here. “Extent” would have been better; we have revised accordingly.**
    
    6.    Figure 3, 4, etc. There is something odd about the plots. By definition, FRET goes from 0 to 1. Yet this FRET goes above 1. How can that be?

**The reviewer is correct that FRET is only defined on the interval [0,1]. However, in the presence of noise the underlying fluorescence values fluctuate stochastically. These fluctuations do not reflect changes in inter-fluorophore distance, but rather arise due to camera shot noise and photon emission statistics. So the observed FRET states are always distributions. When the FRET states are near the limits of the [0,1] interval, the tails of the distributions will extend beyond that interval. We prefer to use the interval [-0.1,1.1] in our plots so that the entire FRET distribution can be seen.**
    
    7.    Figure 4. Maybe the phrasing "not predominantly high..." means "not all the FRET came back to the high FRET state". You probably want to state in the figure legend that 'time' in the figure (in seconds) refers to the time after the stop flow step and not the incubation times.

**Agreed. We have made this addition.**
    
    8.    Fig. 5: I have a hard time understanding this cartoon until I saw it was a model. I thought the authors were summarizing their results (not a bad idea, because there are lots of great results). The experiments first mixed the liposomes and viruses and Ca, and only later added low pH. The npc1 was added to the liposome virus mixture. So all the data just what happens in seconds after adding acid, whereas the cartoon in Fig. 5 shows H+ and Ca++ added before the NPC1.

**We have now clarified that Figure 5 was intended to be a model that is consistent with our observations. Based on the smFRET data, we propose that pH and Ca^2+^ shift the conformational equilibrium of GP in favor of an intermediate conformation, which is reversible upon neutralization or removal of Ca^2+^. Only after cleavage and interaction with NPC1 does GP proceed to undergo irreversible conformational changes required for membrane fusion. We have clarified our intentions in the legend of Figure 5 and in the discussion.**
    
    9.    Line 406. What cation in the phosphate buffer for making liposomes?

**We have now indicated that potassium phosphate was used.**
    
    10.    Line 409. What excess of soluble receptor? Was it washed off before liposomes were used? How do we know it was the receptor on the liposome that made for lipid mixing and not the excess?

**Line 409 was not written correctly. What we meant to indicate was that the Ni-NTA DGS in the liposomes was present in 100-fold molar excess over polyhistidine-tagged sNPC1-C, such that the amount of sNPC1-C not bound to the liposomes is insignificant. This has been clarified.**
